# Trends in inequalities in disability in Europe between 2002 and 2017

**DOI:** 10.1136/jech-2020-216141

**Published:** 2021-03-05

**Authors:** Jose R Rubio Valverde, Johan P Mackenbach, Wilma J Nusselder

**Affiliations:** Department of Public Health, Erasmus University Medical Center, Rotterdam, The Netherlands

**Keywords:** disability, health inequalities, education

## Abstract

**Background:**

Monitoring socioeconomic inequalities in population health is important in order to reduce them. We aim to determine if educational inequalities in Global Activity Limitation Indicator (GALI) disability have changed between 2002 and 2017 in Europe (26 countries).

**Methods:**

We used logistic regression to quantify the annual change in disability prevalence by education, as well as the annual change in prevalence difference and ratio, both for the pooled sample and each country, as reported in the European Union Statistics on Income and Living Conditions (EU-SILC) and the European Social Survey (ESS) for individuals aged 30–79 years.

**Results:**

In EU-SILC, disability prevalence tended to decrease among the high educated. As a result, both the prevalence difference and the prevalence ratio between the low and high educated increased over time. There were no discernible trends in the ESS. However, there was substantial heterogeneity between countries in the magnitude and direction of these changes, but without clear geographical patterns and without consistency between surveys.

**Conclusions:**

Socioeconomic inequalities in disability appear to have increased over time in Europe between 2002 and 2017 as per EU-SILC, and have persisted as measured by the ESS. Efforts to further harmonise disability instruments in international surveys are important, and so are studies to better understand international differences in disability trends and inequalities.

## Introduction

Describing changes in disability and its inequalities between socioeconomic groups over time is an important task of population health monitoring with potentially important social security implications. The Global Activity Limitation Indicator (GALI) is a disability indicator used since 2000 for population health monitoring in Europe.^1 2^ It has been collected in national and European surveys and used for international comparisons of socioeconomic inequalities in disability^3^ and disability-free life expectancy (healthy life years) in the European Union (EU).^4–6^ GALI has been found to be reasonably reliable,^7 8^ making it appropriate to track inequalities in disability and assess progress towards reducing them.

Studies about trends in socioeconomic inequalities in disability have mostly focused on the elderly, among whom disability is often measured in terms of (Instrumental) Activities of Daily Living. In the USA, most studies have found decreasing disability trends, though not at similar speed across all socioeconomic groups, indicating increasing disability inequality.^9–12^ Evidence from European countries is varied. More adverse old age disability trends for lower socioeconomic status (SES) groups relative to higher SES groups have been observed in England^13 14^ and Barcelona,^15^ while trends are similar across education groups in Finland^16^ and Norway,^17^ indicating widening to persisting inequalities. There is more heterogeneity between countries in old age inequality trends for non-European countries.^18–20^ There are no studies that include younger individuals from European countries, though there is a study investigating inequality trends including individuals aged over 40 in the USA that found socioeconomic inequalities in disability narrowed between 2001 and 2011.^21^

We use individual-level data from the annual European Union Statistics on Income and Living Conditions (EU-SILC) and the biennial European Social Survey (ESS) to determine if educational inequalities in disability have changed between 2002 and 2017 in Europe for persons aged 30–79. It adds to the existing literature by using the GALI indicator to explore trends in educational inequalities in the overall European population from two separate surveys, spanning a period of more than a decade and by correcting for changes in the phrasing of the GALI question in EU-SILC. Unlike studies looking at old age disability trends, we include younger individuals in our analyses.

## Methods

### Data

The EU-SILC survey contains annual data on variables related to poverty, income, social exclusion, health and living conditions.^22^ It started in 2004 and covers private households and their members living in the 28 states of the EU. We used the cross-sectional data for all years between 2005 and 2017. We excluded 2004 since fewer countries were included in this year. We excluded countries with more than 3 missing years, leaving 26 countries (online supplemental resource 1). Although EU-SILC is governed by EU regulations, member states can choose to some extent methodology (collection mode, use of proxies) and wording of questions, which can impact disability reporting.^23^

10.1136/jech-2020-216141.supp1 Supplementary data

The ESS is a biennial cross-national survey starting in 2002. It surveys beliefs, health, attitudes and behaviour of individuals of more than 30 countries. The samples are representative of all individuals over 15 years old living in private households.^24^ We included rounds 1–8 of ESS, between 2002 and 2016. We excluded non-EU countries and countries with two or less measurements over the period of study. This left 24 countries included (Romania and Latvia are included in EU-SILC and not in ESS). For both surveys, we included individuals between 30 and 79 years and used individual-level data.

#### Disability

We used the (GALI) question to assess disability. Its standard form is: *‘For at least the past six months, to what extent have you been limited in activities people usually do?’*. We dichotomised ‘Yes, a lot’ and ‘Yes, to some extent’ into one category.

The ESS includes the GALI question but without reference to the 6-month time frame. For EU-SILC, countries varied how they ask the question. Countries like Belgium, France and Ireland have used the standard question for all years, for example. Finland, Norway and the Netherlands changed the question over time.

#### Measure of SES

Both surveys provided International Standard Classification of Education 1997 (ISCED-97) educational attainment. We combined the ISCED categories to form three levels of education: low (0–2), medium (3–4) and high (5–6).^25^

### Statistical analyses

#### Prevalence

For description, we calculated age-standardised prevalence of disability by gender, education, year, country and survey using the 2013 European Standard Population. We also calculated the European average age-standardised GALI disability prevalence by education for each survey by gender, education and year (see online supplemental resource 2 for information on weighting).

We pooled data across countries stratified by survey and gender. We used logistic regression with the GALI indicator as dependent variable to obtain the overall annual change in disability prevalence by education. These regression models (referred to as model 1) included age, age squared, education (low, medium, high), year of the survey, an interaction between education and year, and country as independent variables. In addition, to determine whether the estimates for annual change in prevalence differ significantly between surveys, we conducted t-tests using survey and gender-stratified models.

We conducted a second set of analyses stratified by country.

#### GALI comparability

To account for the heterogeneity in GALI question between countries and across time in EU-SILC, we collected data on GALI comparability for the years 2005–2017 (online supplemental resource 3). We extended model 1 for the pooled models to include a three-level comparability variable (model 2) for EU-SILC.

#### Educational inequalities

From models 1 and 2 we estimated the annual change in prevalence difference and prevalence ratio between the high and low educated using the predicted probabilities from the models derived with the *margins* command in Stata V.15. We also estimated these inequality measures for each individual country.

#### Robustness tests

Because changes in the distribution of education can affect changes in inequalities in disability over time (see online supplemental resource 4), we conducted additional analyses with the relative index of inequality (RII) and slope index of inequality (SII).^26 27^ We also determined whether adding to our models the proportion of each education group in the total population (as measured in each survey) as control variable changed the estimates.

Additional robustness analyses included using only probability weights in the regressions, and for EU-SILC using data after 2008 and removing Latvia and Romania.

## Results

### Prevalence

**Figure 1:**
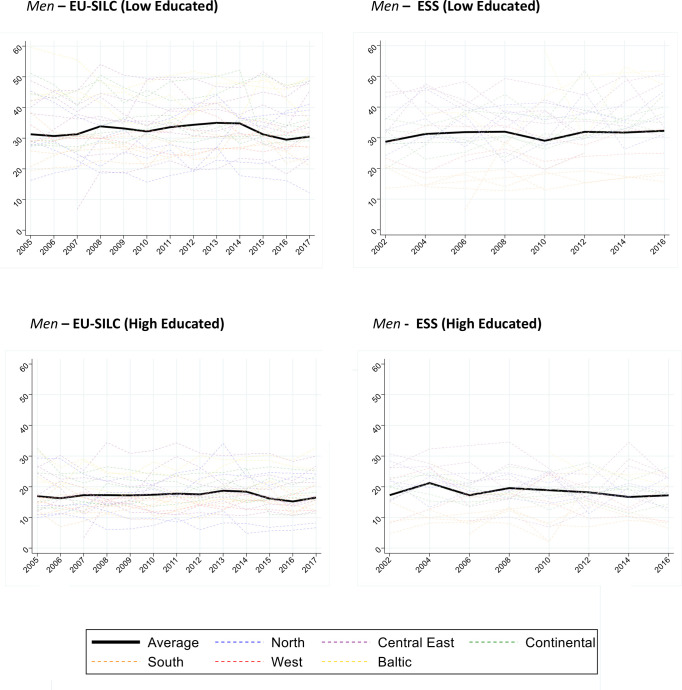
Men. Age-standardised prevalence of Global Activity Limitation Indicator (GALI) disability (ages 30–79) for 26 countries and pooled sample, by gender, education and survey (European Union Statistics on Income and Living Conditions (EU-SILC) 2005–2017; European Social Survey (ESS) 2002–2016). The age-standardised prevalences were estimated through direct standardisation using 2013 European Standard Population. The estimates for ‘Average’ correspond to the population weighted average of the prevalence of all countries. ESS uses the product of the post-stratification weights and the population weights, and country normalised weights are used in EU-SILC. European regions are colour coded (Blue/North: Finland, Sweden, Norway, Denmark; Red/West: UK, Ireland; Green/Continental: Netherlands, Belgium, Germany, Austria, France; Orange/South: Portugal, Spain, Italy, Greece, Cyprus; Purple/Central East: Czechia, Slovenia, Slovakia, Hungary, Poland, Bulgaria, Romania; Yellow/Baltic: Lithuania, Latvia, Estonia).

**Figure 2:**
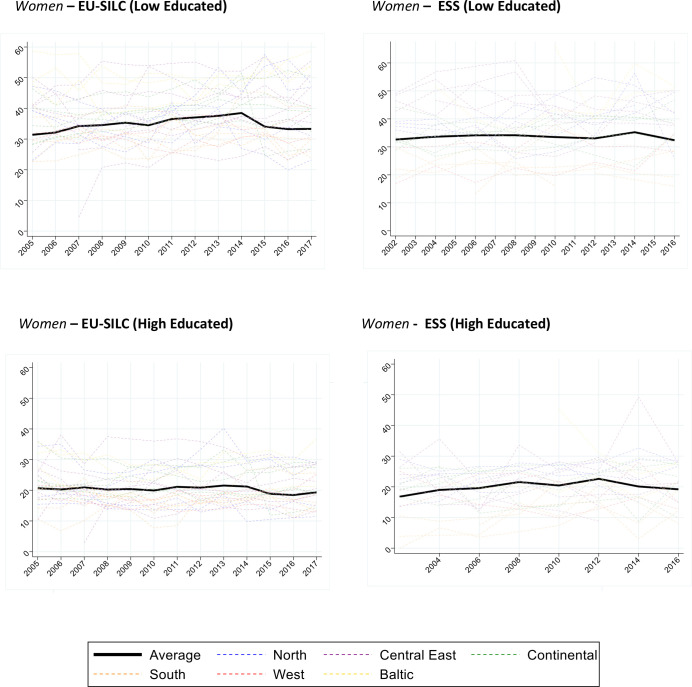
Women. Age-standardised prevalence of Global Activity Limitation Indicator (GALI) disability (ages 30–79) for 26 countries and pooled sample, by gender, education and survey (European Union Statistics on Income and Living Conditions (EU-SILC) 2005–2017; European Social Survey (ESS) 2002–2016). The age-standardised prevalences were estimated through direct standardisation using 2013 European Standard Population. The estimates for ‘Average’ correspond to the population weighted average of the prevalence of all countries. ESS uses the product of the post-stratification weights and the population weights, and country normalised weights are used in EU-SILC. European regions are colour coded (Blue/North: Finland, Sweden, Norway, Denmark; Red/West: UK, Ireland; Green/Continental: Netherlands, Belgium, Germany, Austria, France; Orange/South: Portugal, Spain, Italy, Greece, Cyprus; Purple/Central East: Czechia, Slovenia, Slovakia, Hungary, Poland, Bulgaria, Romania; Yellow/Baltic: Lithuania, Latvia, Estonia).

Figures 1 and 2 show the age-standardised disability prevalence (ages 30–79) by survey (left panel for EU-SILC and right panel for ESS), gender, country and education over time (online supplemental resources 5 and 6).

Figure 1 shows a slightly increasing prevalence for low-educated men and a flat to slightly decreasing trend for high-educated men. Figure 2 shows a slightly upward trend for low-educated women and a slightly downward trend for high-educated women. In ESS, average levels and trends for Europe as a whole were largely similar. In both surveys, there was huge variability between countries, which often had somewhat erratic patterns over time.

Table 1 shows the average annual change in disability prevalence by education for the country-pooled models, stratified by survey and gender. For men in EU-SILC, model 1 estimates that low educated experienced an average increase of 1.3% points in prevalence per decade (0.13%×10), whereas high educated experienced a decrease of 1.5% points per decade. In model 2, correcting for changes in GALI phrasing, the change for the low educated is reduced to a non-statistically significant increase of 0.4% points decennially, while the high educated decreased by 0.7% points. Among women in EU-SILC, changes in disability prevalence were also more unfavourable among low than high educated. For an overview of the effect of the inclusion of the GALI disability correction variable on prevalence, see online supplemental resource 7.

**Table 1:** Global Activity Limitation Indicator (GALI) prevalence change (% point) in 1 year (ages 30–79) for the pooled sample of 26 European countries, by education, gender and survey (European Union Statistics on Income and Living Conditions 2005–2017; European Social Survey 2002–2016) and t-tests of differences between the surveys

Men	EU-SILC (2005–2017)* n=1 809 402			ESS (2002–2016)† n=110 603		Difference between surveys (2)−(1)¶
Education level	(1)	(2)‡	Average GALI prevalence§	(1)	Average GALI prevalence§	
Low (95% CI)	**0.13** (0.08 to 0.18)	0.04 (−0.01 to 0.10)	30.48 (30.06 to 30.89)	−0.02 (−0.19 to 0.16)	27.54 (26.21 to 28.87)	0.06 (−0.17 to 0.28)
Medium (95% CI)	**−0.06** (−0.09 to −0.02)	−0.02 (−0.06 to 0.02)	26.04 (25.72 to 26.36)	0.11 (−0.01 to 0.23)	27.19 (26.00 to 29.74)	−0.12 (−0.29 to 0.06)
High (95% CI)	**−0.15** (−0.19 to −0.11)	**−0.07** (−0.12 to −0.02)	18.42 (18.01 to 18.83)	0.01 (−0.14 to 0.15)	18.78 (17.17 to 23.93)	−0.08 (−0.27 to 0.11)

Women	EU-SILC (2005–2017)* n=2 064 508			ESS (2002–2016)† n=128 401		Difference between surveys (2)−(1)¶
Education level	(1)	(2)‡	Average GALI prevalence§	(1)	Average GALI prevalence§	
Low (95% CI)	**0.10** (0.06 to 0.14)	0.00 (−0.05 to 0.05)	34.74 (34.33 to 35.15)	0.02 (−0.13 to 0.17)	31.07 (29.83 to 32.31)	−0.02 (−0.23 to 0.17)
Medium (95% CI)	−**0.13** (−0.17 to −0.09)	−**0.09** (−0.14 to −0.05)	28.31 (27.99 to 28.63)	**0.17** (0.05 to 0.29)	28.03 (26.80 to 29.27)	−**0.25** (−0.43 to −0.07)
High (95% CI)	−**0.16** (−0.21 to −0.10)	−**0.13** (−0.18 to −0.07)	22.21 (21.75 to 22.66)	−0.00 (−0.18 to 0.18)	21.95 (19.98 to 23.93)	−0.13 (−0.35 to 0.11)

Significant at the 95% level in bold.

Estimates are obtained from gender-stratified logistic models using microdata with the dichotomous GALI indicator as dependent variable:

(1) logit(GALI)=b0+b1(age)+b2(age)(age)+b3(education)+b4(year)+b5(year*education)+b6(country).

(2) logit(GALI)=b0+b1(age)+b2(age)(age)+b3(education)+b4(year)+b5(year*education)+b6(country)+b7(GALI comparability).

The annual change in prevalence by education is estimated by fitting the logistic regressions with all countries pooled (using the product of the population and survey weights in the regression), and after estimation, the command *margins edu_3cat, dydx(year)* gives the average marginal (partial) effects. This means that the effects are calculated for each observation in the data and then averaged. The annual change in prevalence difference is estimated by subtracting the marginal (partial) effects of the low and high educated.

The annual average change in prevalence ratio is estimated by predicting the prevalence of disability by education and year after fitting the logistic models using the *margins* command and the prevalence ratios, and then calculating the average change over the period of study.

*European Union Statistics on Income and Living Conditions (EU-SILC) annual microdata between 2005 and 2017. Member countries in EU-SILC use variants of the GALI question over time.

†European Social Survey (ESS) biannual microdata between 2002 and 2016. ESS uses the same version of the GALI question for all countries and years. This version omits the 6-month time frame of the standard GALI question.

‡GALI comparability estimates include baseline model plus a three-level categorical variable related to phrasing (comparable, partially comparable, not comparable) for EU-SILC only.

§Average age-standardised GALI prevalence over the corresponding period for each survey using the 2013 European Standard Population for all countries included in the sample.

¶Two-sample t-test between the EU-SILC and ESS coefficients.

The ESS finds no statistically significant trends for both genders and education levels. The t-tests in table 1 indicate that the Europeans of average annual change in disability prevalence do not differ statistically between surveys.

The results of the country-stratified models for the annual change in disability prevalence (online supplemental resources 8 and 9) show substantial heterogeneity between countries within a survey and for the same country between surveys.

### Educational inequalities

**Figure 3:**
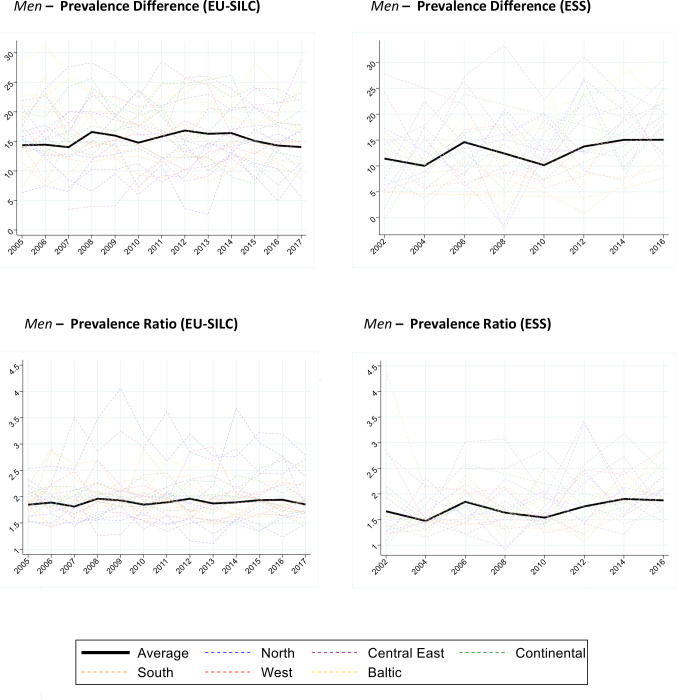
Men. Prevalence difference (low−high) and prevalence ratio (low/high) of Global Activity Limitation Indicator (GALI) disability (ages 30–79) for 26 countries and pooled sample, by gender, education and survey (European Union Statistics on Income and Living Conditions (EU-SILC) 2005–2017; European Social Survey (ESS) 2002–2016). The prevalence difference is estimated as the simple difference of the age-standardised (using 2013 European Standard Population) prevalences (low−high educated). The prevalence ratio is estimated as the ratio of the prevalences (low/high educated). The ‘Average’ corresponds to the population weighted average of the prevalence difference and ratio of all countries. European regions are colour coded (Blue/North: Finland, Sweden, Norway, Denmark; Red/West: UK, Ireland; Green/Continental: Netherlands, Belgium, Germany, Austria, France; Orange/South: Portugal, Spain, Italy, Greece, Cyprus; Purple/Central East: Czechia, Slovenia, Slovakia, Hungary, Poland, Bulgaria, Romania; Yellow/Baltic: Lithuania, Latvia, Estonia).

**Figure 4:**
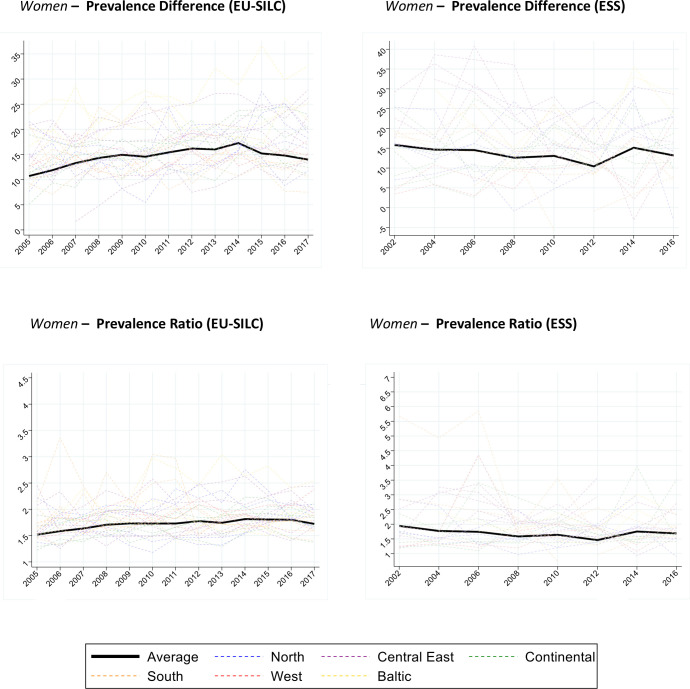
Women. Prevalence difference (low−high) and prevalence ratio (low/high) of Global Activity Limitation Indicator (GALI) disability (ages 30–79) for 26 countries and pooled sample, by gender, education and survey (European Union Statistics on Income and Living Conditions (EU-SILC) 2005–2017; European Social Survey (ESS) 2002–2016). The prevalence difference is estimated as the simple difference of the age-standardised (using 2013 European Standard Population) prevalences (low−high educated). The prevalence ratio is estimated as the ratio of the prevalences (low/high educated). The ‘Average’ corresponds to the population weighted average of the prevalence difference and ratio of all countries. European regions are colour coded (Blue/North: Finland, Sweden, Norway, Denmark; Red/West: UK, Ireland; Green/Continental: Netherlands, Belgium, Germany, Austria, France; Orange/South: Portugal, Spain, Italy, Greece, Cyprus; Purple/Central East: Czechia, Slovenia, Slovakia, Hungary, Poland, Bulgaria, Romania; Yellow/Baltic: Lithuania, Latvia, Estonia).

Figures 3 and 4 show the trend in prevalence difference (low educated−high educated) and prevalence ratio (low educated/high educated) by gender, survey and country. The average prevalence difference in EU-SILC is around 12%, and 9%–10% in ESS. The average prevalence ratio is around 1.6–1.7 in EU-SILC and 1.4–1.5 in ESS. Among men in EU-SILC, the average prevalence difference and ratio appear virtually constant during the study period. Among women in EU-SILC, the average prevalence difference and ratio show a slight upward slope. In ESS, the trends for the prevalence difference and ratio appear constant for both genders. Here again, we see substantial heterogeneity between countries and sometimes erratic patterns in single countries.

Table 2 presents the regression-based results for the change in prevalence difference and prevalence ratio over time. For men, EU-SILC model 1 estimates a statistically significant increase of the average prevalence difference of 2.8% points decennially. After the GALI comparability correction, model 2 estimates a statistically significant increase of 1.1% points decennially. The corresponding estimate from ESS is a non-statistically significant decrease of 0.3% points per decade. The average prevalence ratios have also increased over time according to EU-SILC. The differences between the two surveys are not statistically significant for the difference and the ratio.

**Table 2:** Global Activity Limitation Indicator (GALI) educational inequalities (prevalence difference in % points and prevalence ratio) change in 1 year (ages 30–79) for the pooled sample of 26 European countries, by education, gender and survey (European Union Statistics on Income and Living Conditions 2005–2017; European Social Survey 2002–2016) and t-tests between survey estimates

Men	EU- SILC (2005–2017)* n=1 885 712			ESS (2002–2016)† n=110 603		Difference between surveys (2)−(1)‡
Δ inequality in 1 year	(1)	(2)§	Average inequality¶	(1)	Average inequality¶	
Prevalence difference (95% CI)	**0.28** (0.21 to 0.34)	**0.11** (0.03 to 0.17)	12.05 (11.26 to 12.84)	−0.03 (−0.27 to 0.21)	9.92 (9.25 to 11.95)	0.14 (−0.14 to 0.41)
Prevalence ratio (95% CI)	**0.026** (0.017 to 0.028)	**0.010** (0.004 to 0.015)	1.650 (1.591 to 1.710)	−0.003 (−0.015 to 0.004)	1.532 (1.421 to 1.701)	−0.013 (−0.030 to 0.004)

Women	EU- SILC (2005–2017)* n=2 064 508			ESS (2002–2016)† n=128 401		Difference between surveys (2)−(1)‡
Δ inequality in 1 year	(1)	(2)§	Average inequality¶	(1)	Average inequality¶	
Prevalence difference (95% CI)	**0.26** (0.19 to 0.32)	**0.12** (0.04 to 0.18)	12.53 (11.76 to 13.36)	0.02 (−0.21 to 0.23)	9.19 (7.13 to 11.05)	0.10 (−0.19 to 0.38)
Prevalence ratio (95% CI)	**0.016** (0.011 to 0.019)	**0.009** (0.004 to 0.012)	1.563 (1.522 to 1.612)	0.001 (−0.013 to 0.015)	1.421 (1.30 to 1.54)	0.008 (−0.010 to 0.025)

Significant at the 95% level in bold.

Estimates are obtained from gender-stratified logistic models using microdata with the dichotomous GALI indicator as dependent variable:

(1) logit(GALI)=b0+b1(age)+b2(age)(age)+b3(education)+b4(year)+b5(year*education)+b6(country).

(2) logit(GALI)=b0+b1(age)+b2(age)(age)+b3(education)+b4(year)+b5(year*education)+b6(country)+b7(GALI comparability).

The annual change in prevalence by education is estimated by fitting the logistic regressions with all countries pooled (using the product of the population and survey weights in the regression), and after estimation, the command *margins edu_3cat, dydx(year)* gives the average marginal (partial) effects. This means that the effects are calculated for each observation in the data and then averaged. The annual change in prevalence difference is estimated by subtracting the marginal (partial) effects of the low and high educated.

The annual average change in prevalence ratio is estimated by predicting the prevalence of disability by education and year after fitting the logistic models using the *margins* command and the prevalence ratios, and then calculating the average change over the period of study.

*European Union Statistics on Income and Living Conditions (EU-SILC) annual microdata between 2005 and 2017. Member countries in EU-SILC use variants of the GALI question over time.

†European Social Survey (ESS) biannual microdata between 2002 and 2016. ESS uses the same version of the GALI question for all countries and years. This version omits the 6-month time frame of the standard GALI question.

‡Two-sample t-test between the EU-SILC and ESS coefficients.

§GALI comparability estimates include baseline model plus a three-level categorical variable related to phrasing (comparable, partially comparable, not comparable) for EU-SILC only.

¶Average age-standardised GALI prevalence over the corresponding period for each survey using the 2013 European Standard Population for all countries included in the sample.

For women in EU-SILC, model 2 estimates a statistically significant increase of the average prevalence difference of 1.2% points decennially. The corresponding estimate from ESS is a non-statistically significant increase of 0.2% points decennially. The difference between the two surveys is not statistically significant for the prevalence difference and ratio.

The results for the SII and RII trends (online supplemental resource 10) are similar to those for the prevalence difference and ratio, with statistically significant increases in EU-SILC and non-statistically significant increases in ESS.

### Robustness

The results are robust to several changes (online supplemental resource 11). The inclusion of the proportion of education as a covariate or using only probability weights in the analysis does not change the conclusions of the main analyses. For EU-SILC, the trends are attenuated if analysed only after 2008, yet they remain statistically significant. The results are also robust to excluding Romania and Latvia.

## Discussion

### Summary of results

There was a clear educational gradient in disability prevalence for individuals between 30 and 80 years old. In EU-SILC, disability prevalence tended to decrease among the high educated and remained constant for the low educated. Both the prevalence difference and the prevalence ratio between low and high educated increased over time. The increase in prevalence difference amounts to more than a 1% point in 10 years. There were no discernible trends in the ESS, though trends were not significantly different between surveys. There was substantial heterogeneity between countries in the magnitude and direction of these changes in prevalence and inequalities, but without clear geographical patterns and without consistency between surveys.

### Comparison to other studies

Directly comparing our results to other studies is complicated by differences in study populations, the instrument used to measure disability and the period of study. Furthermore, there are no studies to our knowledge that look at the overall European population trends in disability by education. Most studies in Europe and the USA include only older individuals. Our estimates for Europe are in line with most of the literature of old age disability that finds individuals with a lower socioeconomic position have higher levels of disability relative to individuals with a higher position, and that these differences are persistent and in most cases are not narrowing over time.^11–13 16 17 28^ A study from California in the USA between 2001 and 2011 including individuals over age 45 finds that while racial inequalities in disability widened, the gap between the most and least socioeconomically advantaged appeared to have narrowed.^21^ Our study suggests that some European countries have experienced more favourable trends than others, but differences between countries may be due to differences in measurement (eg, sampling procedures, survey administration) and reporting. Our results are consistent with prior studies on trends in educational inequalities of self-assessed health in European countries.^29 30^

Differences in disability prevalence between socioeconomic strata are likely due to differences in the presence of disabling health conditions, and their interaction with environmental and personal factors.^31^ An important risk factor for health conditions and disability is high body weight, which has been linked to the development of musculoskeletal disease^32^ and mental health afflictions.^33^ A study looking at educational differences in trends in obesity in several European countries between 1990 and 2010 finds that obesity increased for most countries, and the low educated were more adversely affected.^34^ Widening inequalities in obesity could explain part of the widening of inequalities in disability that we observe in this study. Other important explanations include widening socioeconomic inequalities in smoking^35^ as well as in material conditions.^36^

While there is less agreement in disability trends by education between the unadjusted EU-SILC estimates and the ESS estimates, the inclusion of the GALI phrasing correction brings the EU-SILC estimates closer to those of ESS, with a less sharp increase in prevalence for low educated and a decreasing trend for the high educated. The adjusted EU-SILC and ESS estimates do not differ significantly, even for women, with less similar trends between surveys than for men. The impact of the correction is not as strong on the change in absolute and relative inequalities, since the change in phrasing seems to impact low and high educated in a similar way. The only significant difference between surveys for the inequality trends occurs for medium-educated women. Since there is no gold standard survey, it is hard to say whether EU-SILC or ESS is closer to the underlying population trend in prevalence by education and their absolute and relative inequalities, though we can conclude from both that inequalities are not narrowing for either genders.

Changes in the distribution of education could also drive trends in disability prevalence and inequalities. After accounting for this explicitly by including the per cent of the sample for each education level in the model, the results were not significantly changed. In addition, the results for the SII and RII trends are similar to those based on prevalence ratio and difference.

Although we focused on estimating the overall trends for Europe, it is important to note that there is substantial heterogeneity in both levels and trends of disability by education between individual countries, and in many cases they do not agree between surveys. For example, according to our EU-SILC estimates for men, the UK showed a statistically significant decrease in absolute inequality (6.2% points decennially), yet the trend is flat according to ESS (−0.2% points decennially).

### Strengths and limitations

A strength of our study is using the GALI indicator. GALI refers implicitly to social participation in several settings like work and leisure.^37^ It is relevant throughout the life course and was developed to be easily included in international surveys.^38^ This allowed us to study disability in Europe, including younger age groups and using two European surveys, each with its strength and weaknesses.

Advantages of using EU-SILC are the large sample sizes and that it provides yearly data for most countries. However, its output-harmonised nature introduces heterogeneity in sampling and data collection. For example, while most countries use household surveys, a few countries (Finland, the Netherlands, Norway, Slovenia, Sweden) use administrative registers supplemented with interviews. Moreover, some countries (Spain, Ireland) allow for replacement of non-respondents, and one country (Germany) uses a combination of quota and random sampling. Weights are provided in EU-SILC to adjust for the probability of selection into the sample; however, it is unclear for which countries these weights also correct for non-response.^39^ Advantages of ESS include consistency in mode and sampling between countries. ESS uses strict random probability methods. Disadvantages of ESS are its smaller sample sizes, that it is biennial and that not all countries are included in all survey rounds (ie, Italy, Lithuania, Bulgaria, Cyprus). Finally, there are imbalances across age groups and countries in how well the ESS represents the target populations. This is generally reduced by using poststratification weights, but not for all countries.^40^ It is hard to state that one survey is preferable to the other given their merits and limitations. Further research into how variation in sampling, data collection and phrasing impact disability estimates would provide insight into comparability issues.

A limitation of this study is that GALI prevalence can be impacted by changes in phrasing of the question.^41 42^ Whereas in ESS there were no such changes, they were present in EU-SILC. We added a three-level comparability variable to our model for EU-SILC. A limitation of this correction is heterogeneity in the type of questions that are included in the partially comparable and not comparable categories. Our study did not distinguish between levels of severity of disability. Studying how trends vary by levels of severity of disability would provide additional information on disability progression.^43^ Furthermore, disability is self-reported and is subject to heterogeneity in tendency to report health problems.^44^ Further research looking at trends in inequalities using other disability instruments, including performance-based measures, would provide more insight into socioeconomic differences in disability in Europe.

## Conclusion and implications

Socioeconomic inequalities in disability appeared to have increased or at least persisted over time in Europe. This is detrimental to equity and may have important implications for the sustainability of the social security and pension system. Since the low educated are more likely to exit the labour market early due to disability, failure to reduce inequalities in disability impacts disability pension expenditure and may hamper current efforts of many countries to increase the retirement age, in response to sustainability problems of the pension system. These individual and society consequences call for further action to close these gaps. Furthermore, efforts to harmonise disability instruments in international surveys are important, allowing for more meaningful international comparisons of disability and to track progress in reducing them.

**What is already known on this subject**

- Evidence of trends in socioeconomic inequalities in disability is limited and the existing evidence is varied. Different countries, age groups and indicators used to measure disability limit comparability between studies. We use a standardised disability measurement and include two international surveys to investigate trends in educational inequalities in disability in Europe.

**What this study adds**

- There is no evidence of a reduction in the educational inequalities in disability in Europe, with one survey indicating an increase and the other no significant change over time. Harmonisation of disability measurements and the collection of internationally comparable data is important for tracking population health inequalities.

## Data Availability

Data may be obtained from a third party and are not publicly available. Availability of data and materials: the following data from the European Social Survey were used: ESS Round 1: European Social Survey Round 1 Data (2002), data file edition 6.6; ESS Round 2: European Social Survey Round 2 Data (2004), data file edition 3.6; ESS Round 3: European Social Survey Round 3 Data (2006), data file edition 3.7; ESS Round 4: European Social Survey Round 4 Data (2008), data file edition 4.5; ESS Round 5: European Social Survey Round 5 Data (2010), data file edition 3.4; ESS Round 6: European Social Survey Round 6 Data (2012), data file edition 2.4; ESS Round 7: European Social Survey Round 7 Data (2014), data file edition 2.2; ESS Round 8: European Social Survey Round 8 Data (2016), data file edition 2.1; Norwegian Centre for Research Data (NSD), Norway–data archive and distributor of ESS data for ESS ERIC. This study is based on data from Eurostat, European Union Statistics on Income and Living Conditions survey, reference years 2005–2017.
