# Supplementary material for: Trends in inequalities in disability in Europe between 2002 and 2017
Source: J Epidemiol Community Health. 2021 Mar 5;75(8):712–20. doi: 10.1136/jech-2020-216141 (PMC8292565; doi:10.1136/jech-2020-216141)
Supplement: Supplementary data [file jech-2020-216141supp001.pdf]

Journal of Epidemiology and Community Health

Trends in inequalities in disability in Europe between 2002-2017

Supplementary Files

Online Resource 1

Table S1.1 Sample Size by country (26 countries), year and survey (ages 30-79) - European Union Statistics on Income and Living Conditions (EU-SILC)

Country	2005	2006	2007	2008	2009	2010	2011	2012	2013	2014	2015	2016	2017	Total
Austria	8424	9795	10307	8507	8587	8950	8957	8975	8591	8440	8578	8517	8588	115216
Belgium	7220	8418	8450	8887	8927	8912	8616	8419	8848	8646	8658	8163	8293	110457
Bulgaria			7696	7862	10054	11061	11748	10010	8542	8469	8316	12355	12379	108492
Cyprus	6606	6451	5999	5683	5365	6401	6702	7853	7914	7345	7345	7048	7241	87953
Czech R.	6356	11124	13903	15916	13642	12104	11050	11001	9834	9793	9341	9782	9766	143612
Germany	20520	21650	21416	19492	19278	19048	19624	19262	18420	18434	19569	20011	20060	256784
Denmark	4899	4718	4666	4691	4747	4740	4281	4361	4408	4659	4866	5068	4809	60913
Estonia	7089	9488	8235	7533	7840	7830	7916	8461	8926	9099	8836	9310	9151	109714
Greece	9567	9832	9122	10467	11256	11094	9521	8853	11611	13935	22938	29557	36160	193913
Spain	22610	21041	20319	21380	21973	22118	20967	20465	19436	20261	20804	23690	22701	277765
Finland	8749	7659	7394	8085	7764	8415	7155	7735	8596	8602	8097	8338	7976	104565
France	14908	15350	14612	15032	15130	15757	16050	17013	15660	16104	15986	16109	15582	203293
Hungary	11675	13135	14033	14140	15454	15624	18615	18063	16169	14584	12154	12459	12532	188637
Ireland	9522	9101	8041	7529	7307	6566	6169	6570	7077	8027	8065	7820	7502	99296
Italy	38811	38129	33584	33508	32652	30715	29917	29982	28299	30471	28086	31255	31845	417254
Lithuania	7532	8035	8276	7748	8263	8720	8375	8602	8067	8168	7493	7356	7448	104083
Latvia	6194	7123	6903	8075	8923	9528	9974	9695	9277	8947	8848	8867	8554	110908
Netherlands	7888	7752	8565	8662	8141	8493	8692	8412	8422	8479	8247	10262	10601	112616
Norway	4692	4544	4526	4123	4079	3963	3481	4610	4603	5591	4886	5191	4695	58984
Poland	27998	26240	24049	23322	21884	21422	21251	21822	21263	21458	19918	19142	20478	290247
Portugal	6832	6609	6390	6831	7661	8048	8815	9551	10224	12480	15862	19199	21832	140334
Romania			13045	12710	12585	12568	12155	12076	12424	11943	12500	12587	12550	137143
Sweden	4681	5057	5382	5600	5716	5447	5129	5020	4715	4413	4483	4473	4617	64733
Slovenia	6429	7492	6613	6885	7198	7303	7243	7201	7069	7214	6860	6771	6987	91265
Slovakia	9161	9105	8793	9555	9426	9746	9329	9557	9484	9696	10135	10432	10370	124789
UK	15168	14115	12910	11926	10603	10381	10956	12757	12801	13616	12279	13492	10200	161204
Total	273531	281963	293229	294149	294455	294954	292688	296326	290680	298874	303150	327254	332917	3874170

Rubio Valverde JR, *et al. J Epidemiol Community Health* 2021; 75:712–720. doi: 10.1136/jech-2020-216141

Table S1.2 Sample Size by country (26 countries), year and survey (ages 30-79) - European Social Survey (ESS)

Country	2002	2004	2006	2008	2010	2012	2014	2016	Total
Austria	1762	1607	1709				1417	1605	8100
Belgium	1308	1305	1334	1294	1253	1363	1276	1289	10422
Bulgaria			1092	1841	2001	1950			6884
Cyprus			728	883	776	839			3226
Czech R.	1055	2271		1576	1831	1582	1699	1784	11798
Germany	2285	2180	2199	2189	2305	2247	2385	2211	18001
Denmark	1143	1151	1202	1284	1214	1241	1120		8355
Estonia		1442	1097	1206	1331	1769	1574	1546	9965
Greece	1987	1904		1605	2053				7549
Spain	1303	1162	1320	1893	1404	1490	1480	1532	11584
Finland	1457	1524	1404	1636	1393	1694	1601	1487	12196
France	1139	1434	1586	1600	1348	1582	1482	1626	11797
Hungary	1220	1140	1178	1146	1197	1533	1352	1252	10018
Ireland	1517	1731	1202	1397	1882	2041	1861	2205	13836
Italy	916					682		1943	3541
Lithuania					1231	1620	1771	1674	6296
Latvia				1512					1512
Netherlands	1963	1555	1516	1431	1509	1482	1522	1324	12302
Norway	1576	1351	1289	1187	1152	1216	1063	1127	9961
Poland	1405	1143	1166	1111	1186	1356	1187	1274	9828
Portugal	1154	1540	1717	1825	1693	1716	1007	1027	11679
Romania				1558					1558
Sweden	1491	1431	1416	1350	1133	1369	1311	1200	10701
Slovenia	1080	995	1060	927	1038	940	937	997	7974
Slovakia		952	1150	1459	1502	1531			6594
UK	1615	1353	1796	1801	1793	1707	1763	1499	13327
Total	27376	29171	27161	33711	32225	32950	27808	28602	239004

Online Resource 2

Table S2.1 Average population weight by country (26 countries), year and survey- European Union Statistics on Income and Living Conditions (EU-SILC 2005-2017) and European Social Survey (ESS 2002-2016)

Country	EU-SILC*	ESS**
Austria	0,43	0,32
Belgium	0,56	0,50
Bulgaria	0,39	0,31
Cyprus	0,06	0,06
Czech R.	0,54	0,41
Denmark	0,29	0,29
Estonia	0,07	0,06
Finland	0,28	0,22
France	3,35	2,73
Germany	4,24	2,42
Greece	0,57	0,38
Hungary	0,52	0,51
Ireland	0,23	0,15
Italy	3,06	3,20
Latvia	0,11	0,10
Lithuania	0,16	0,13
Netherlands	0,86	0,72
Norway	0,25	0,23
Poland	1,97	1,85
Portugal	0,54	0,46
Romania	1,06	0,85
Slovakia	0,28	0,26
Slovenia	0,11	0,13
Spain	2,35	1,96
Sweden	0,49	0,43
UK	3,24	2,31

*Population weights are constructed using the average of the total populations of the 26 countries as base (1) by year and country. Data were obtained from the World Development Indicators Database from the World Bank.

**Population weights are provided by the ESS and their used is encouraged when pooling data from several European countries, with careful consideration of their use.

We used the product of survey normalized probability weights and population weights for all calculations involving the average for Europe. The population weights for EU-SILC were calculated using the total population of the 26 countries over the period of study obtained from the World Development Indicators [25]. Unlike EU-SILC, the ESS provides their own population weight

Online Resource 3

Table S3.1 Global Activity Limitation Indicator (GALI) Comparability to standard question, by country (27 countries) and year – European Union Statistics for Income and Living Conditions (EU-SILC 2005-2017)

Country	Year												
	2005	2006	2007	2008	2009	2010	2011	2012	2013	2014	2015	2016	2017
Austria	PC	PC	PC	C	C	C	C	C	C	C	C	C	C
**Belgium**	C	C	C	C	C	C	C	C	C	C	C	C	C
Bulgaria			NC	C	C	C	C	C	C	C	C	C	C
Switzerland				C	C	C	C	C	C		C	C	
Cyprus	NC	PC	PC	PC	PC	PC	PC	PC	PC	C	C	C	C
Czechia	NC	NC	NC	C	C	C	C	C	C	C	C	C	C
Germany	NC	NC	NC	NC	NC	NC	NC	NC	NC	NC	PC	PC	PC
Denmark	NC	NC	NC	C	C	C	C	C	C	C	C	C	C
Estonia	NC	PC	PC	C	C	C	C	C	C	C	C	C	C
Greece	PC	PC	PC	PC	PC	PC	PC	C	C	C	C	C	C
Spain	PC	PC	PC	C	C	C	C	C	C	C	C	C	C
Finland	NC	PC	PC	C	C	C	C	C	C	C	C	C	C
**France**	C	C	C	C	C	C	C	C	C	C	C	C	C
Hungary	PC	PC	PC	NC	NC	NC	NC	NC	PC	PC	PC	PC	PC
**Ireland**	C	C	C	C	C	C	C	C	C	C	C	C	C
Italy	PC	PC	C	C	C	C	C	C	C	C	C	C	C
Lithuania	PC	PC	PC	PC	PC	PC	PC	C	C	C	C	C	C
Latvia	NC	PC	PC	PC	PC	PC	PC	PC	PC	PC	PC	PC	PC
Netherlands	PC	PC	PC	PC	PC	PC	PC	PC	PC	PC	C	C	C
Norway	PC	PC	PC	PC	PC	PC	PC	PC	PC	PC	PC	PC	PC
Poland	PC	PC	PC	PC	C	C	C	C	C	C	C	C	C
Portugal	PC	PC	PC	PC	PC	C	C	C	PC	PC	C	C	C
Romania			PC	PC	PC	C	C	C	C	C	C	C	C
Sweden	NC	NC	NC	C	C	C	C	C	C	NC	NC	NC	NC
Slovenia	NC	NC	NC	NC	NC	C	C	C	C	C	C	C	C
Slovakia	NC	PC	PC	PC	PC	PC	PC	PC	PC	PC	PC	PC	PC
United Kingdom	NC	NC	NC	NC	NC	NC	NC	PC	PC	PC	PC	PC	PC
Legend	C	Comparable			PC	Partially comparable				NC	Not comparable		

GALI comparability for 2005-2012: obtained from the EUROSTAT *document "Overview of the implementation of the GALI question in EU-SILC"*
https://circabc.europa.eu/webdav/CircaBC/ESTAT/health/Library/working_group_2012/documents/Item%209.1%20HLY%20annex%202%20-%20overview%20tables%20and%20notes.pdf
From year 2012-2016: comparability was obtained by extending the assessment done by the prior document if the question was not changed. This is based three sources of information. The first is the document from the European Health and Life Expectancy Information System (EHLEIS) *"EU-SILC Health questions 2014-2016 in national languages and back translations by country experts"* that can be obtained from:
http://www.eurohex.eu/pdf/Reports_2018/2018_TR4%206_SILC%20Questions_Backtranslation.pdf
The second is the document "Health questions from the Minimum European Health Module used in EU-SILC in the 27 countries", and can be obtained from:
http://www.eurohex.eu/pdf/Reports_2014/2014_TR4%205_Health%20Questions.pdf
From year 2015-2017: when additional information was necessary, country specific questionnaires were consulted from the GESIS Microdata Lab documentation, available at:
https://www.gesis.org/en/missy/materials/EU-SILC/documents/questionnaires
Comparability was assessed based on the 4 GALI question criteria : 1)being limited, 2) in activities people do , 3) because of a health problem, 4) for the last 6 months. Countries that had 4/4 were deemed comparable, while 3 partially comparable and below not comparable. If a question fulfilled 4 criteria, but was separated in filters (like the UK in last years) it was also set to partially comparable. This last criterion did not change the assessment for any country.

Table S3.2. Global Activity Limitation Indicator (GALI) change in question relative to prior year, by country and year - European Union Statistics for Income and Living Conditions (EU-SILC 2005-2017)

Country	Year												
	2005	2006	2007	2008	2009	2010	2011	2012	2013	2014	2015	2016	2017
Austria	0	0	0	1	0	0	0	0	0	1	0	0	0
**Belgium**	1	0	0	0	0	0	0	0	0	0	0	0	0
Bulgaria			0	1	0	0	0	0	0	0	0	0	0
Switzerland				1	0	0	1	0	1		0	0	
Cyprus		1	0	1	0	0	0	0	0	1	0	0	0
Czech Republic		0	1	1	0	0	0	0	0	0	0	0	0
Germany		1	0	1	0	0	0	0	0	0	1	0	0
Denmark	0	0	0	1	0	0	0	0	0	0	0	0	0
Estonia	0	1	0	1	0	0	0	0	0	0	0	0	0
Greece	0	0	0	1	0	0	0	0	0	0	0	0	0
Spain	0	0	0	1	0	0	0	0	0	0	0	0	0
Finland	0	0	1	1	0	0	0	0	0	0	0	1	1
**France**	0	0	0	0	0	0	0	0	0	0	0	0	0
Hungary		0	1	1	0	0	0	1	1	0	0	0	0
**Ireland**	0	0	0	0	0	0	0	0	0	0	0	0	0
Italy	0	1	1	0	0	0	0	0	0	0	0	0	0
Lithuania	0	1	1	0	0	0	0	1	0	0	0	0	0
Latvia		1	0	0	0	0	0	0	0	0	0	0	0
Netherlands		0	0	1	0	0	0	0	0	0	1	0	0
Norway	0	0	0	1	0	0	0	0	0	0	0	0	0
Poland		1	0	0	1	0	0	0	0	0	0	0	0
Portugal	1	0	0	1	0	1	0	1	0	0	1	0	0
Romania				0	0	1	0	0	0	0	0	0	0
Sweden	0	1	0	1	0	0	0	0	0	1	0	0	0
Slovenia		0	0	0	0	1	0	0	0	0	0	0	0
Slovakia		1	0	1	0	0	0	0	0	0	1	0	0
United Kingdom		0	0	0	0	0	0	1	0	0	0	0	0
Legend	0	No change			1	Changes from prior year							

GALI comparability for 2005-2012: obtained from the EUROSTAT *document "Overview of the implementation of the GALI question in EU-SILC"*
https://circabc.europa.eu/webdav/CircaBC/ESTAT/health/Library/working_group_2012/documents/Item%209.1%20HLY%20annex%202%20-%20overview%20tables%20and%20notes.pdf

From year 2012-2016: comparability was obtained by extending the assessment done by the prior document if the question was not changed. This is based three sources of information. The first is the document from the European Health and Life Expectancy Information System (EHLEIS) *"EU-SILC Health questions 2014-2016 in national languages and back translations by country experts"* that can be obtained from:
http://www.eurohex.eu/pdf/Reports_2018/2018_TR4%206_SILC%20Questions_Backtranslation.pdf
The second is the document "Health questions from the Minimum European Health Module used in EU-SILC in the 27 countries", and can be obtained from:
http://www.eurohex.eu/pdf/Reports_2014/2014_TR4%205_Health%20Questions.pdf

From year 2015-2017: when additional information was necessary, country specific questionnaires were consulted from the GESIS Microdata Lab documentation, available at:
https://www.gesis.org/en/missy/materials/EU-SILC/documents/questionnaires

Deviations from the standard GALI question phrasing in EU-SILC vary by country and by year, and they vary between omitting dimensions of the question (i.e , 6 month duration, activities people do), using a filter question (i.e the UK between 2005-11, Portugal 2005-07) and changing wording of the question.

Online Resource 4

Figure S4.1 Distribution of sample by education over time (ages 30-79) for 26 countries and for pooled sample - by sex and survey (European Union Statistics on Income and Living Conditions 2005-2017; European Social Survey 2002-2016)

Males **– EU-SILC**

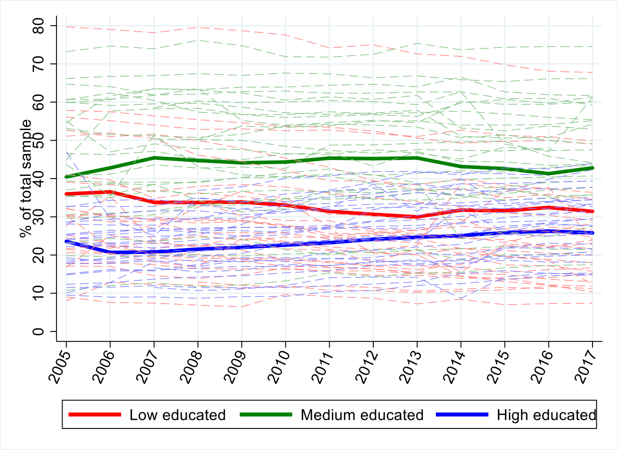

Males **– ESS**

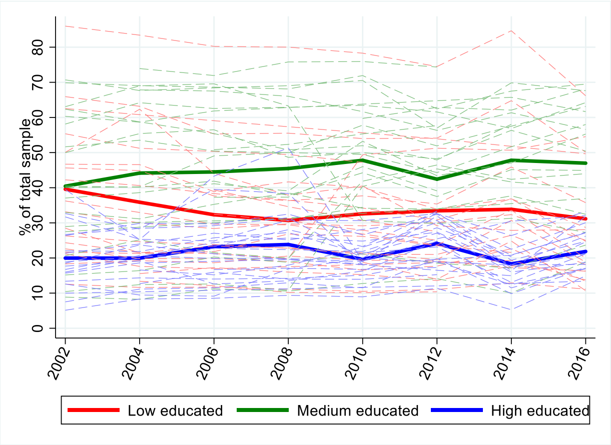

Females **– EU-SILC**

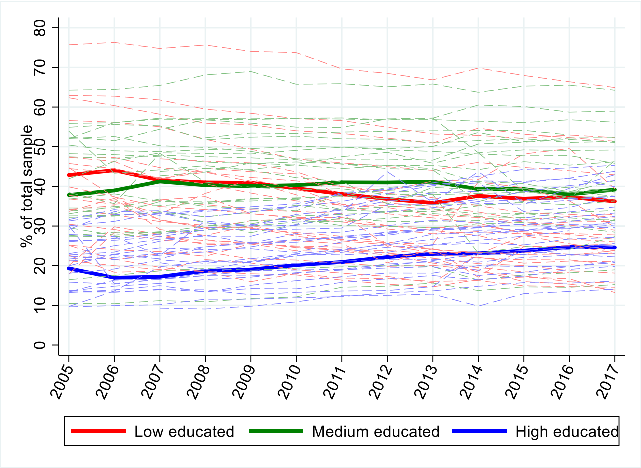

Females **– ESS**

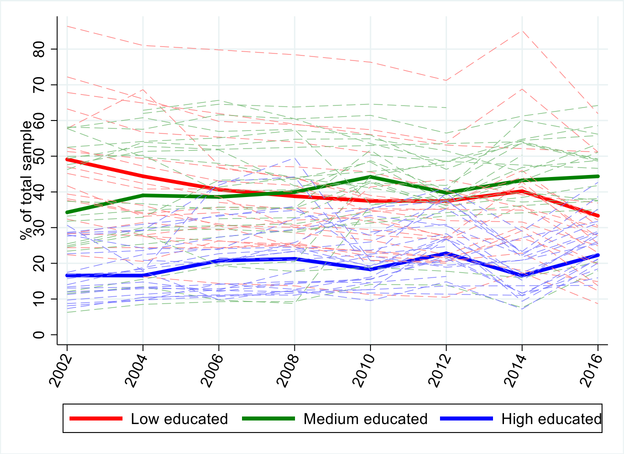

Online Resource 5

Figure S5.1 Age standardized prevalence of GALI (Global Activity Limitation Indicator) Disability (ages 30-79) for medium educated, for 26 countries and pooled sample, sex and survey (European Union Statistics on Income and Living Conditions 2005-2017; European Social Survey 2002-2016

Males **– EU-SILC (Medium Educated)**

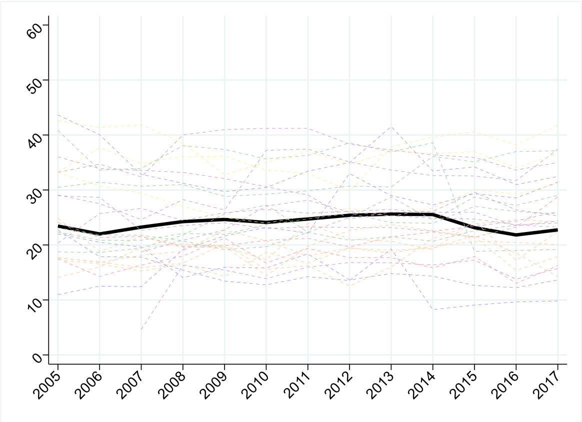

Males **- ESS (Medium Educated)**

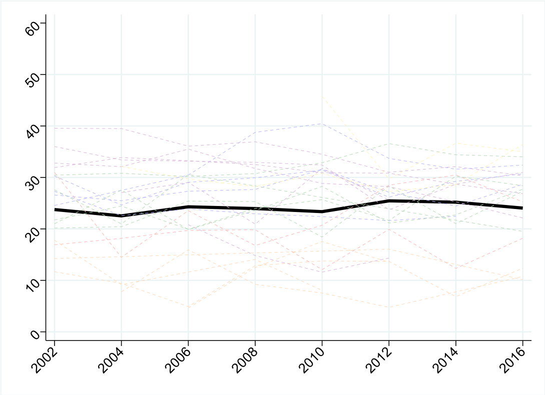

Females **– EU-SILC (Medium Educated)**

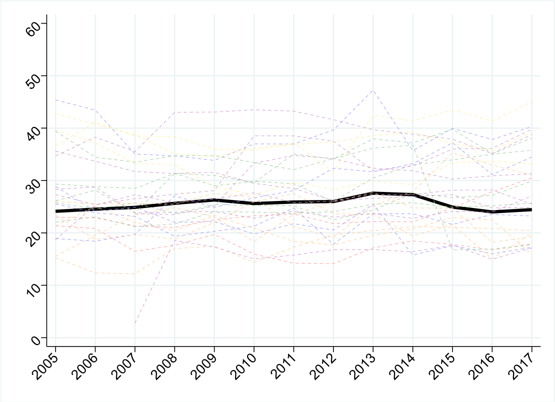

Males **- ESS (Medium Educated)**

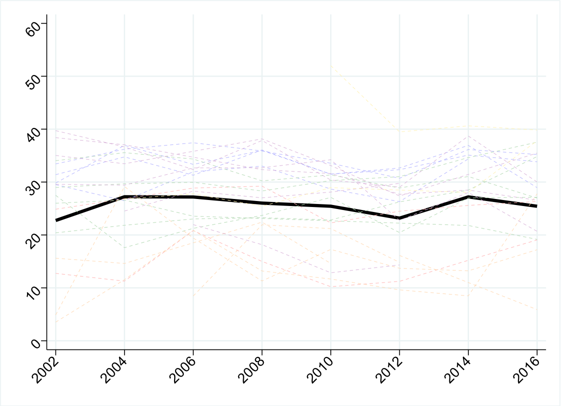

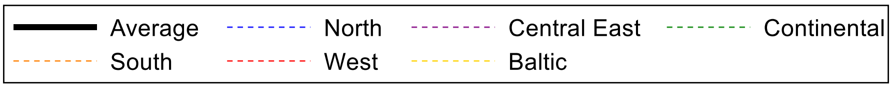

The age standardized prevalences were estimated through direct standardization using 2013 European Standard Population. The estimates for "All countries " correspond to the population weighted average of the prevalence of all countries. ESS uses the product of the post-stratification weights and the population weight, and country normalized weights are used in EU-SILC

European regions are color-coded (**North/Blue**: Finland, Sweden, Norway, Denmark; **Red/West**: UK, Ireland; **Green/Continental**: Netherlands, Belgium, Germany, Austria, France; **Orange/South**: Portugal, Spain, Italy, Greece, Cyprus; **Purple/Central East:** Czechia, Slovenia, Slovakia, Hungary, Poland, Bulgaria, Romania ; **Yellow/Baltic:** Lithuania, Latvia, Estonia

Rubio Valverde JR, *et al. J Epidemiol Community Health* 2021; 75:712–720. doi: 10.1136/jech-2020-216141

Figure S5.2 Age standardized prevalence of GALI (Global Activity Limitation Indicator) Disability (ages 30-79) for low and high educated and their inequalities, for 26 countries and pooled sample, sex and survey (European Union Statistics on Income and Living Conditions 2005-2017; European Social Survey 2002-2016 – by region

<u>Northern Europe</u>

Men

Low Educated – EU-SILC

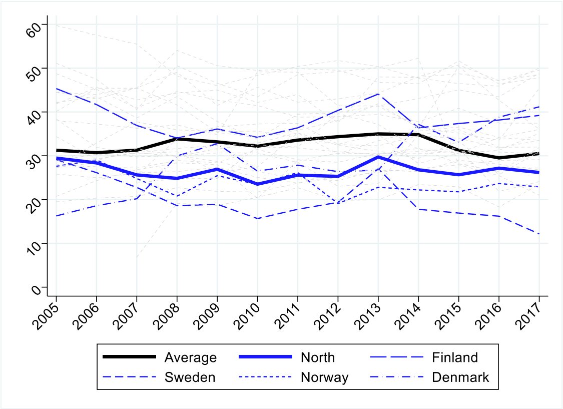

Low Educated - ESS

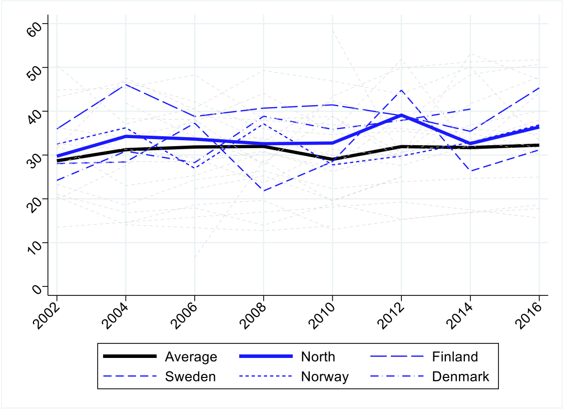

High Educated – EU-SILC

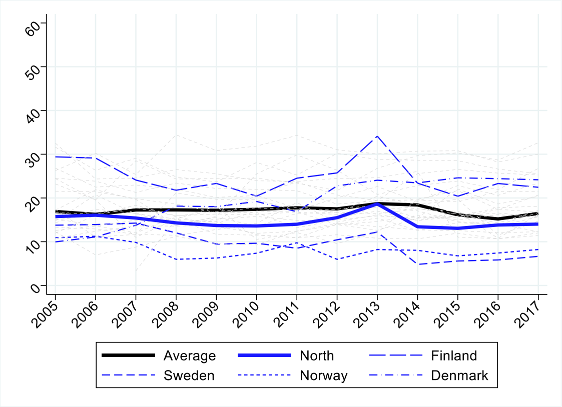

High Educated - ESS

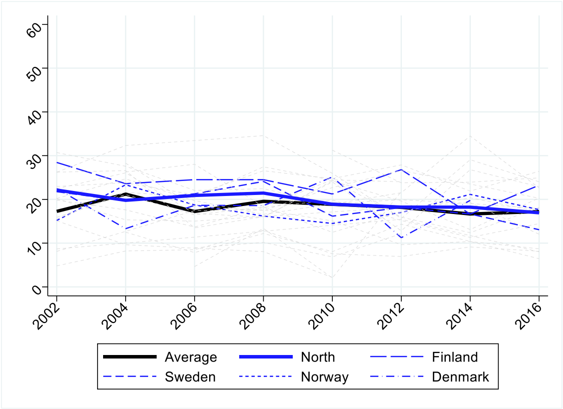

Prevalence Difference –EU-SILC

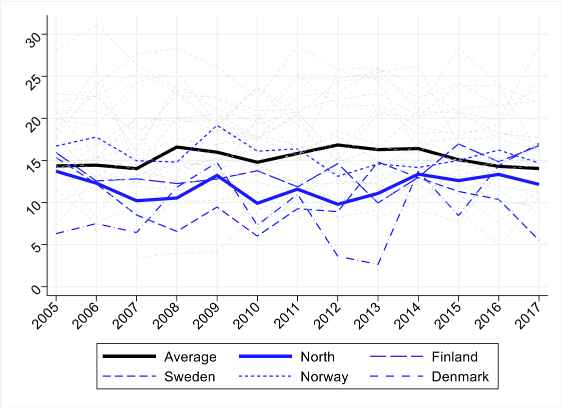

Prevalence Difference - ESS

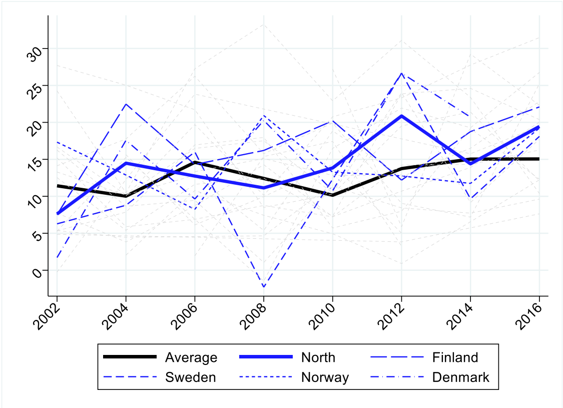

Prevalence Ratio – EU-SILC

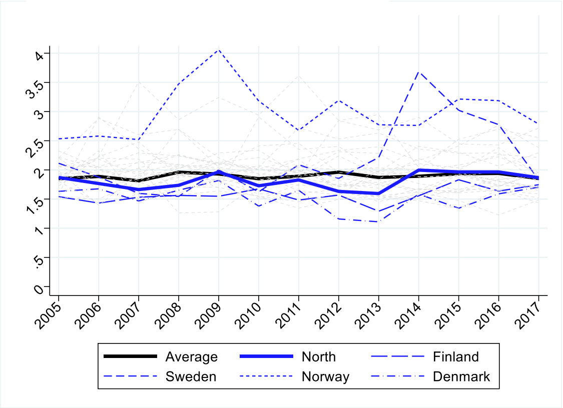

Prevalence Ratio - ESS

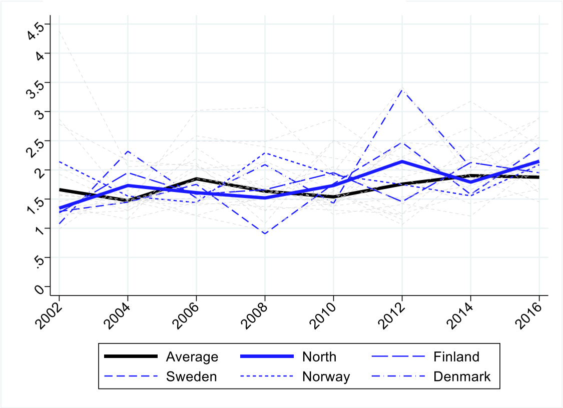

Women

Low Educated – EU-SILC

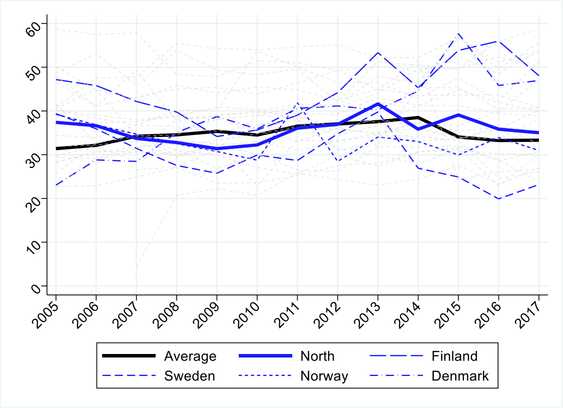

Low Educated - ESS

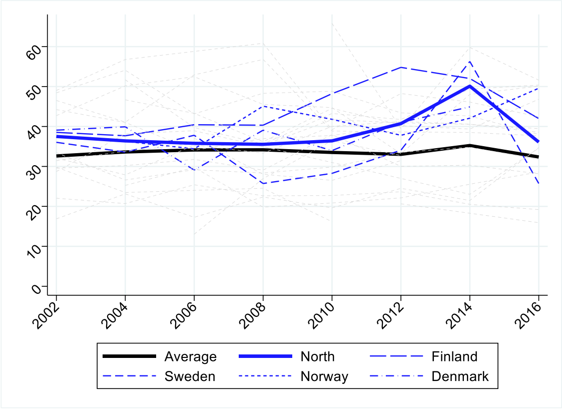

High Educated – EU-SILC

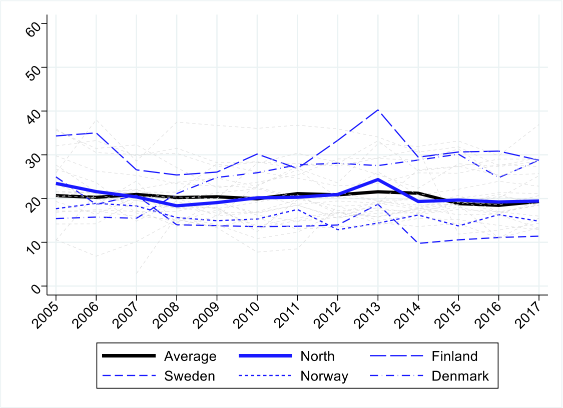

High Educated - ESS

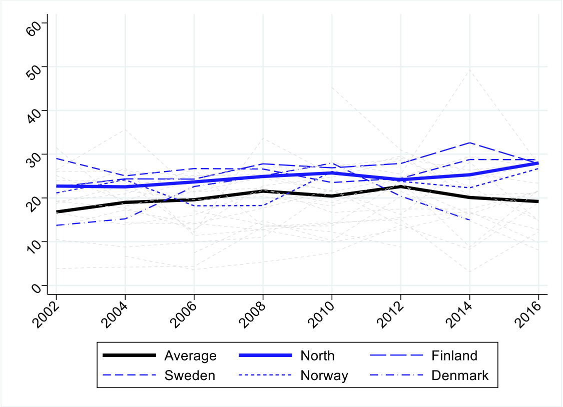

Prevalence Difference – EU-SILC

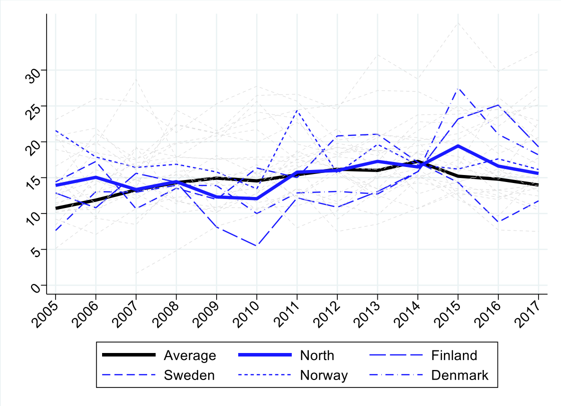

Prevalence Difference - ESS

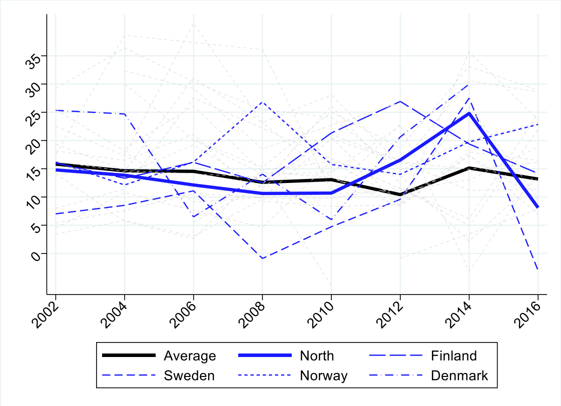

Prevalence Ratio – EU-SILC

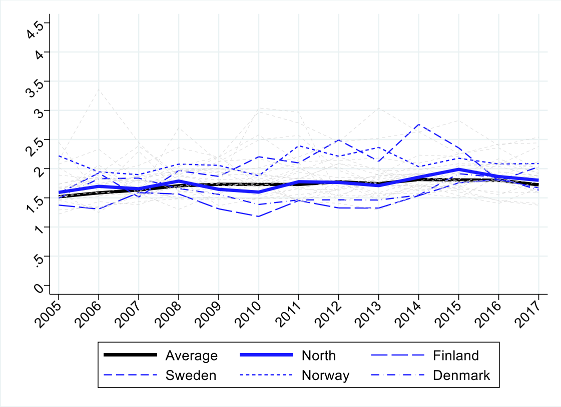

Prevalence Ratio - ESS

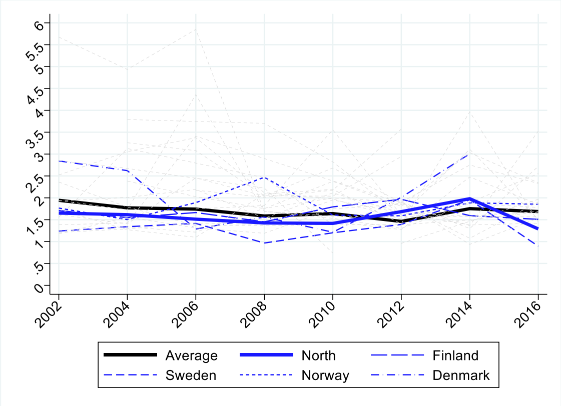

West and Baltic

Men

Low Educated – EU-SILC

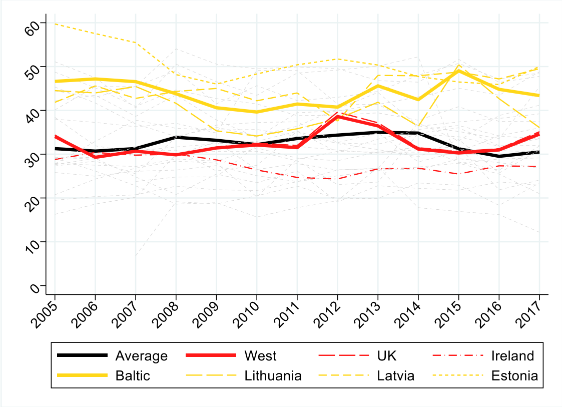

Low Educated - ESS

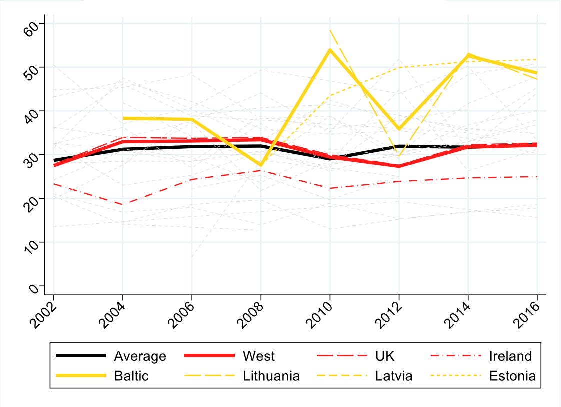

High Educated – EU-SILC

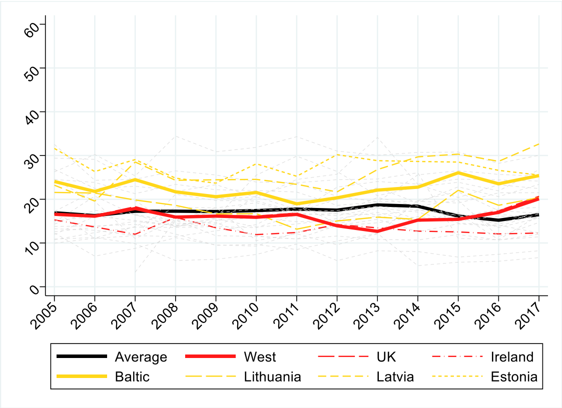

High Educated - ESS

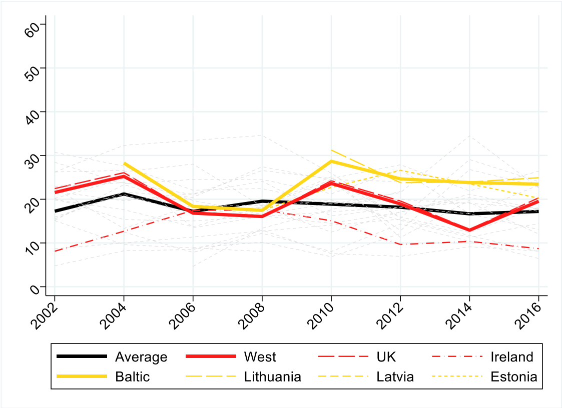

Prevalence Difference – EU-SILC

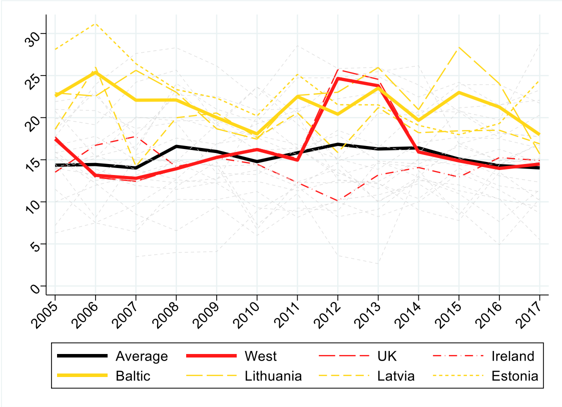

Prevalence Difference - ESS

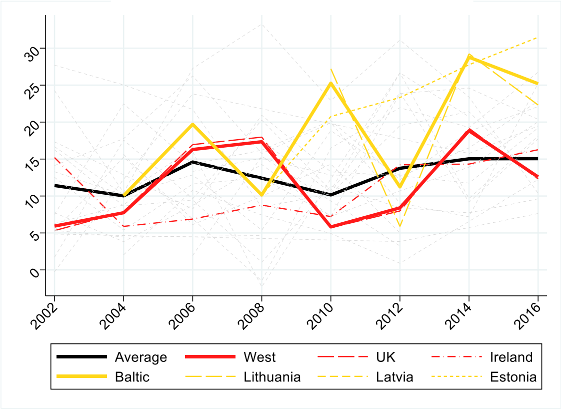

Prevalence Ratio – EU-SILC

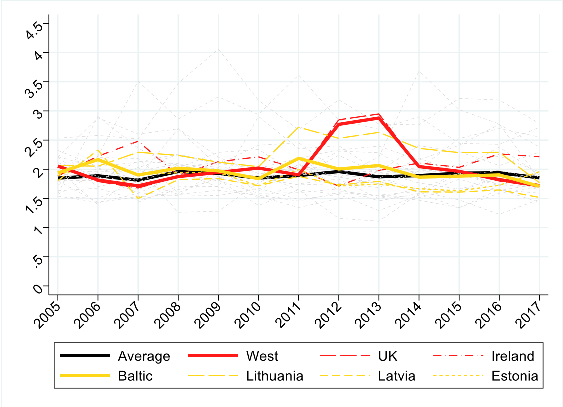

Prevalence Ratio - ESS

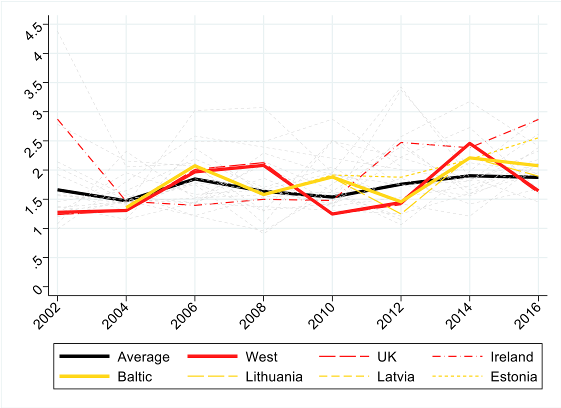

Women

Low Educated – EU-SILC

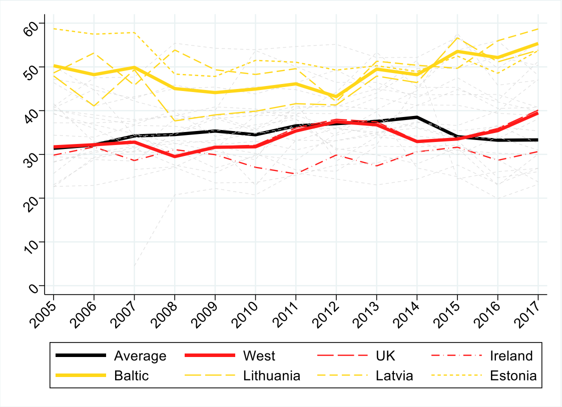

Low Educated - ESS

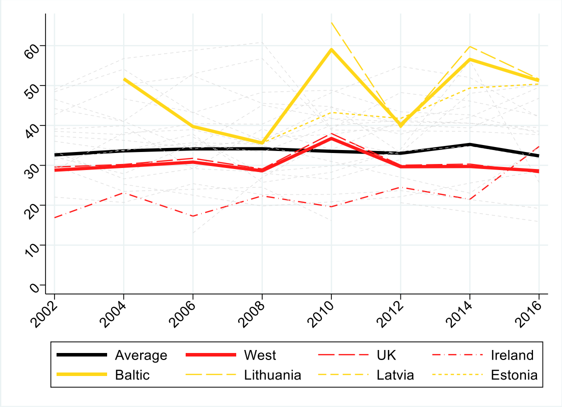

High Educated – EU-SILC

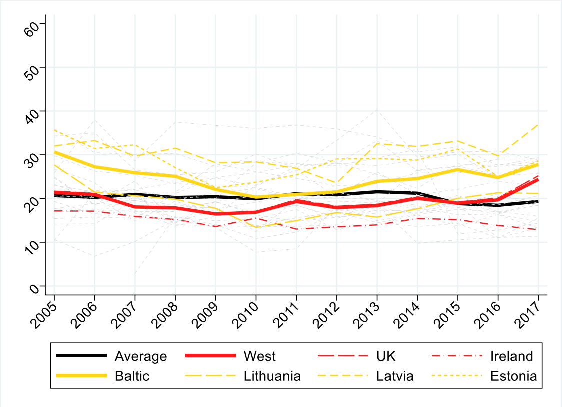

High Educated - ESS

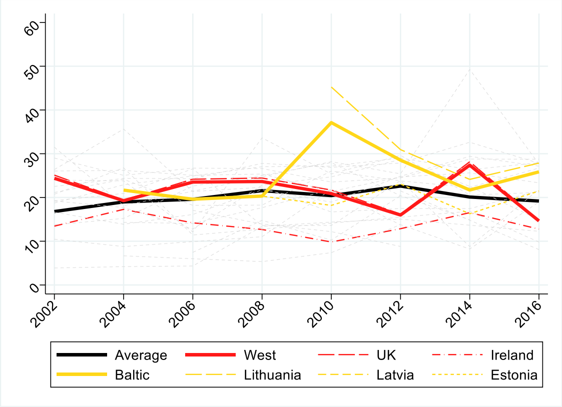

Prevalence Difference – EU-SILC

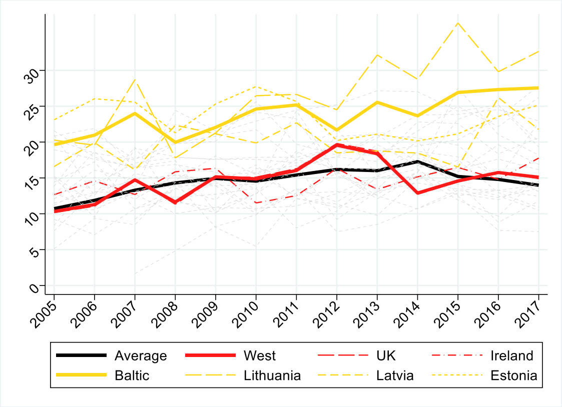

Prevalence Difference - ESS

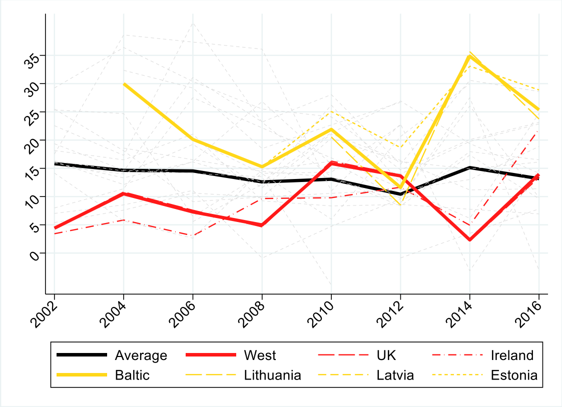

Prevalence Ratio – EU-SILC

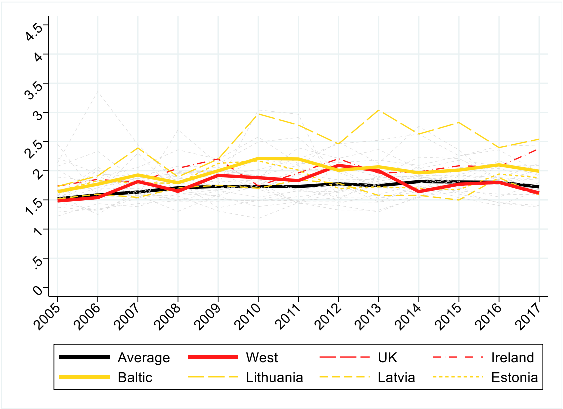

Prevalence Ratio - ESS

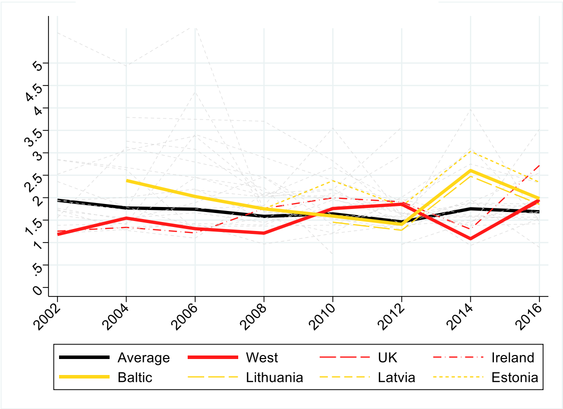

Continental

Men

Low Educated – EU-SILC

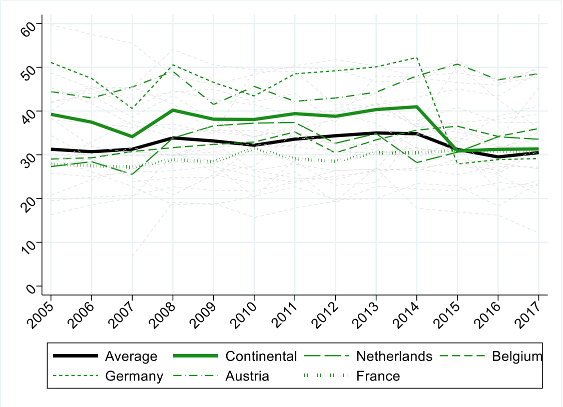

Low Educated - ESS

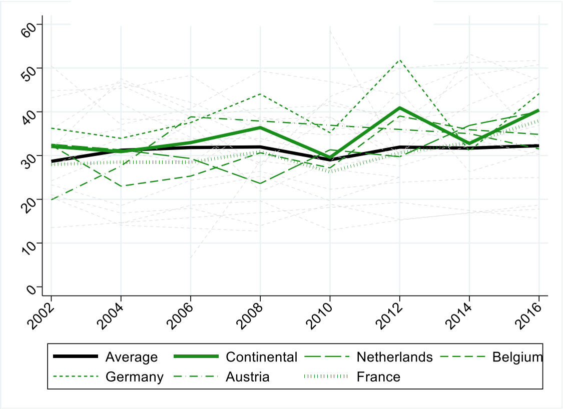

High Educated – EU-SILC

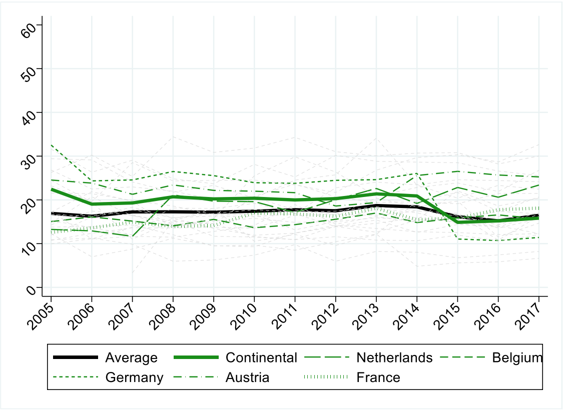

High Educated - ESS

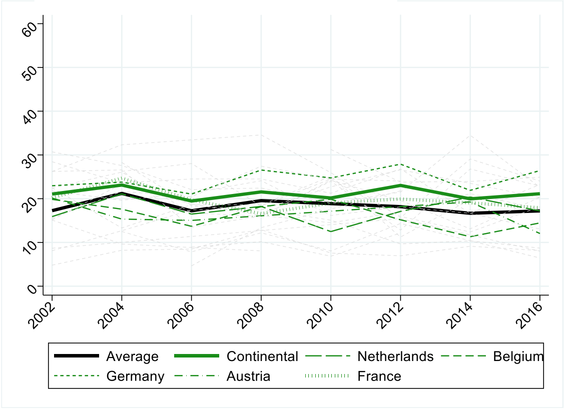

Prevalence Difference – EU-SILC

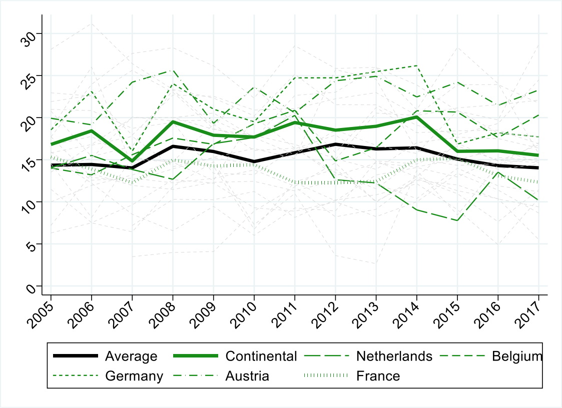

Prevalence Difference - ESS

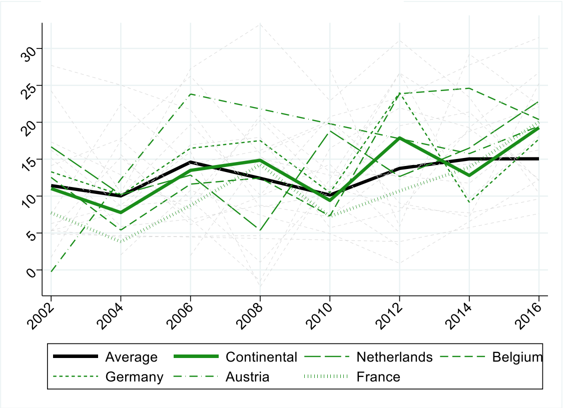

Prevalence Ratio – EU-SILC

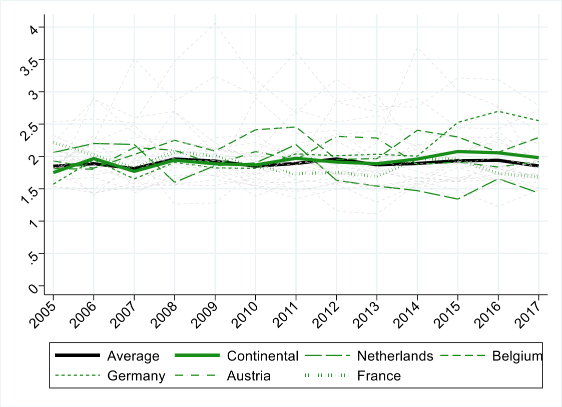

Prevalence Ratio - ESS

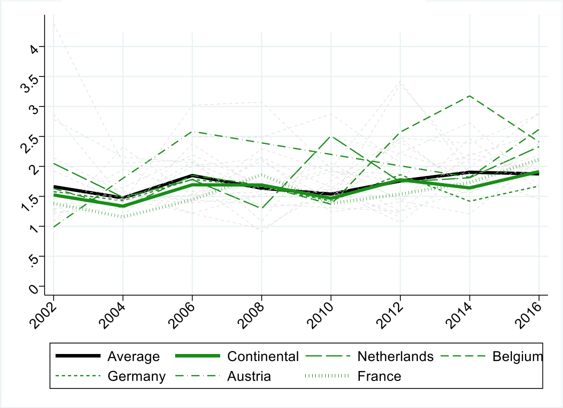

Women

Low Educated – EU-SILC

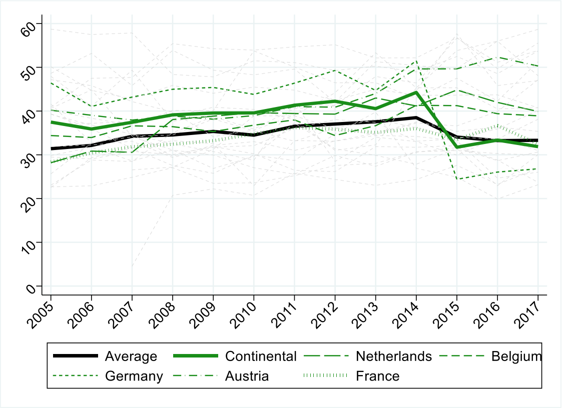

Low Educated - ESS

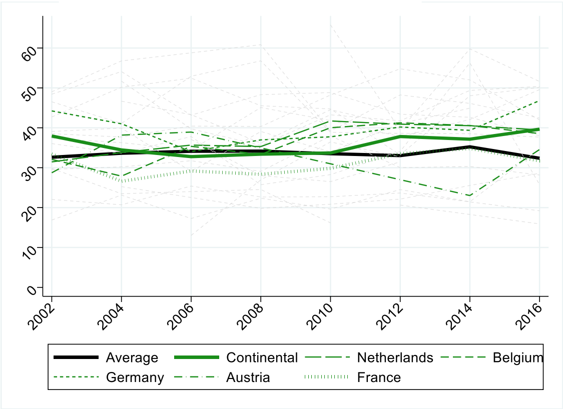

High Educated – EU-SILC

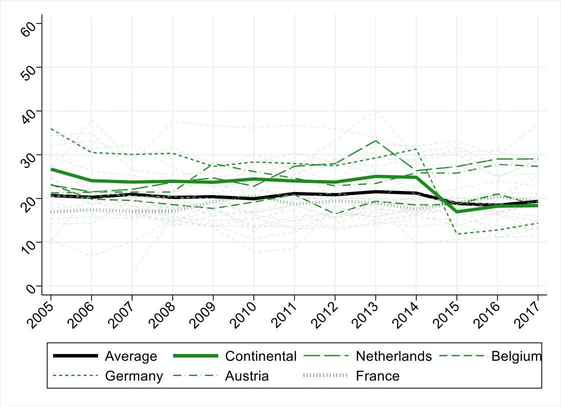

High Educated - ESS

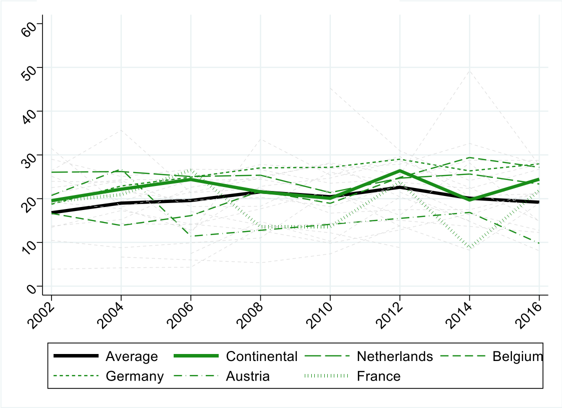

Prevalence Difference – EU-SILC

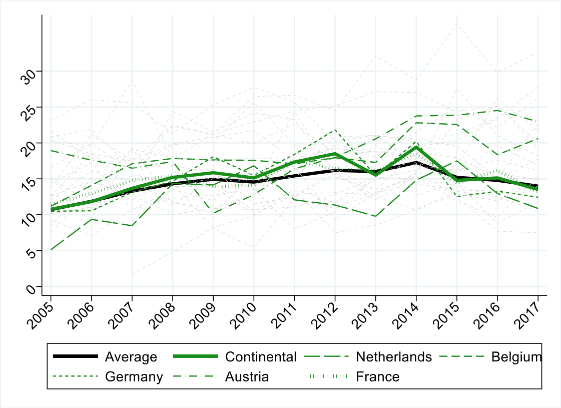

High Educated - ESS

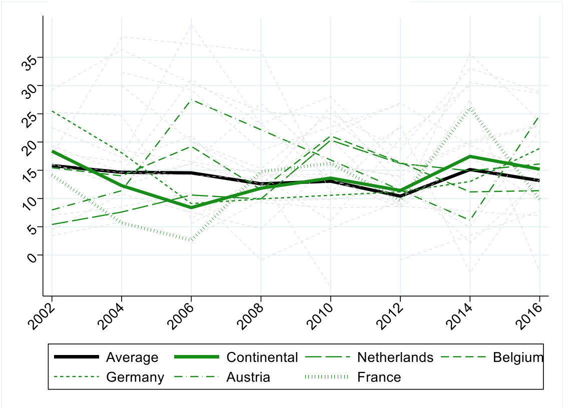

Prevalence Ratio – EU SILC

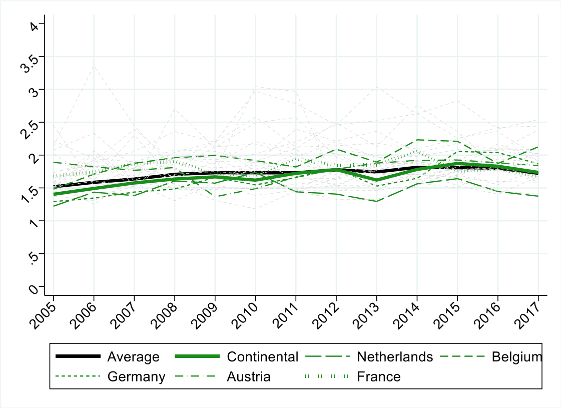

Prevalence Ratio - ESS

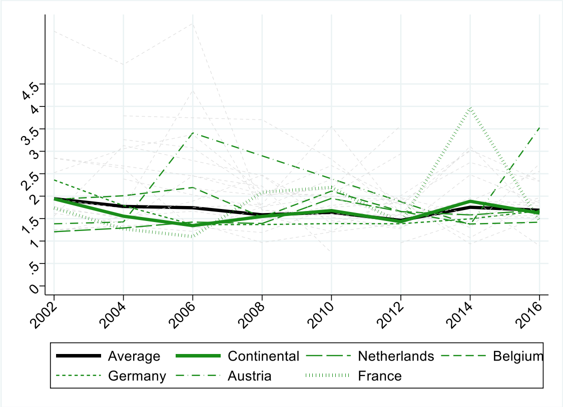

Rubio Valverde JR, *et al. J Epidemiol Community Health* 2021; 75:712–720. doi: 10.1136/jech-2020-216141

Men

Low Educated – EU-SILC

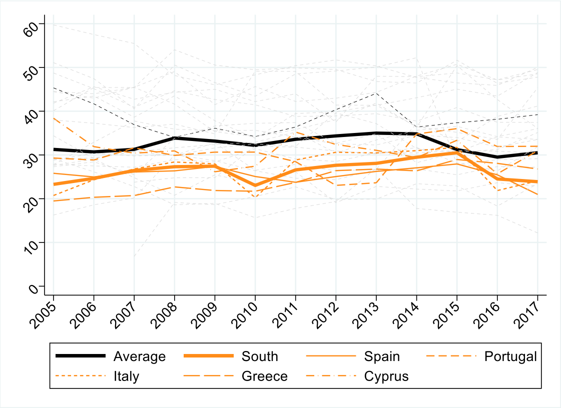

Low Educated - ESS

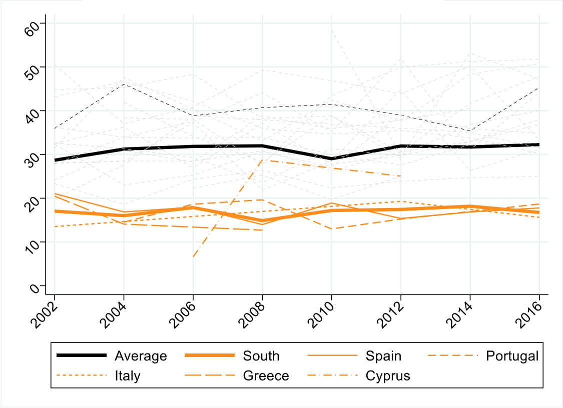

High Educated – EU-SILC

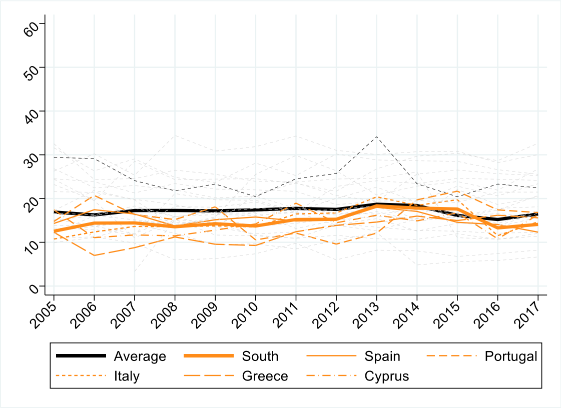

High Educated - ESS

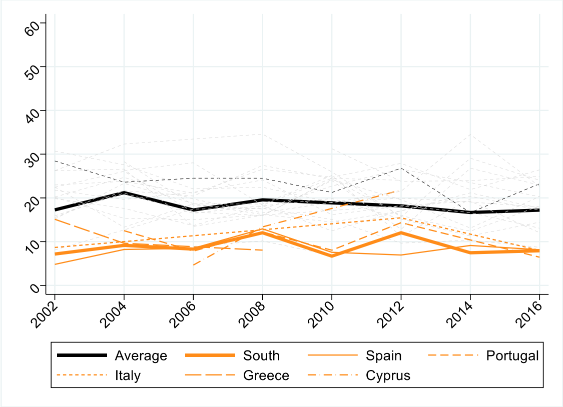

Prevalence Difference – EU-SILC

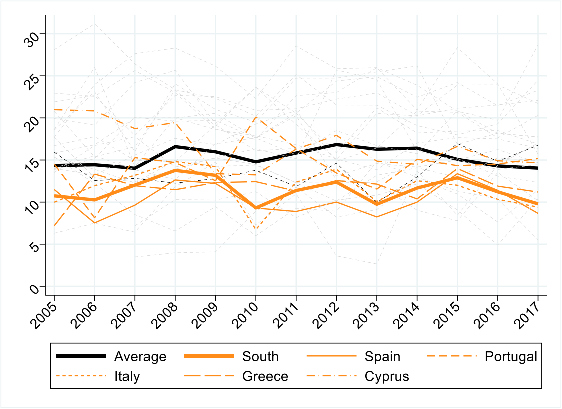

Prevalence Difference - ESS

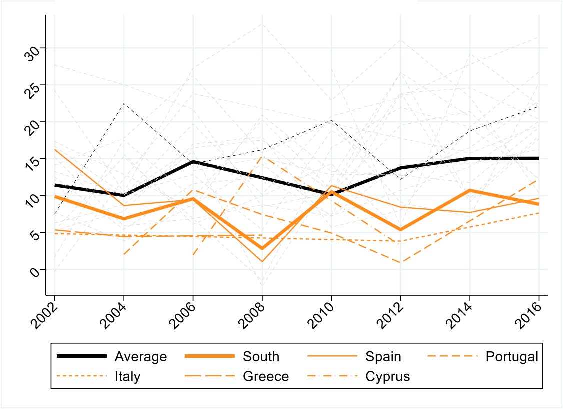

Prevalence Ratio – EU-SILC

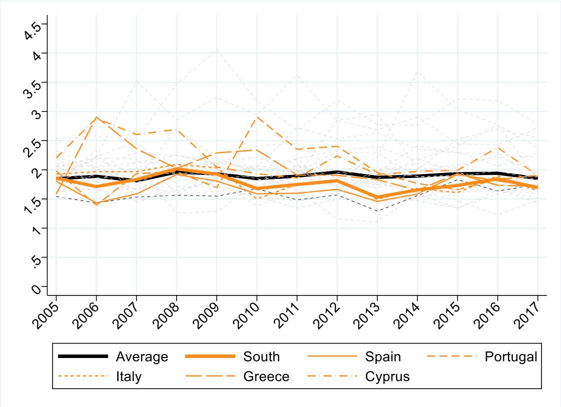

Prevalence Ratio - ESS

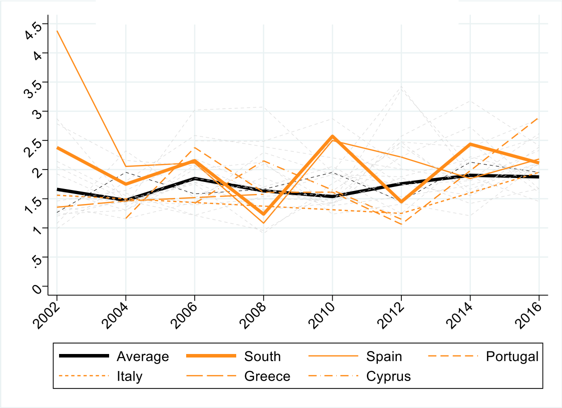

Women

Low Educated – EU-SILC

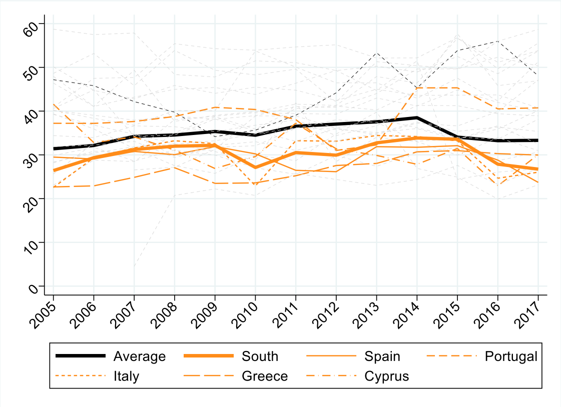

Low Educated - ESS

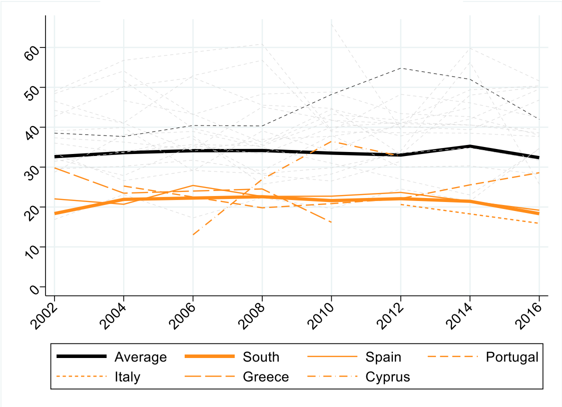

High Educated – EU-SILC

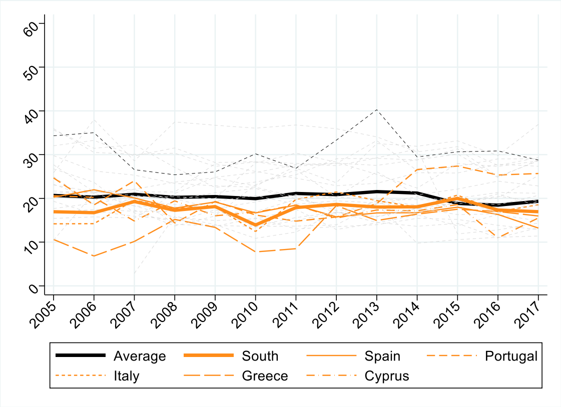

High Educated - ESS

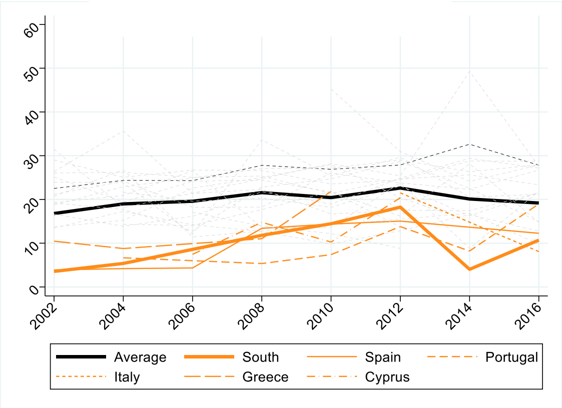

Prevalence Difference – EU-SILC

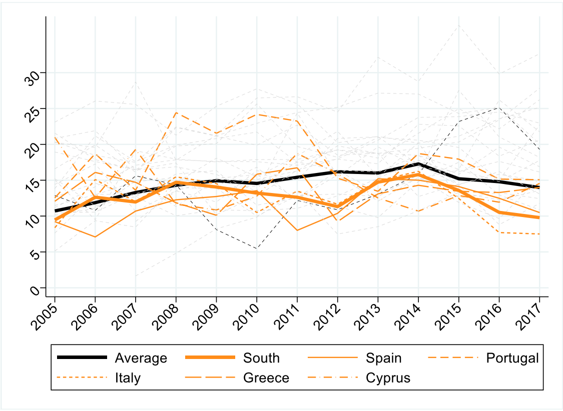

Prevalence Difference - ESS

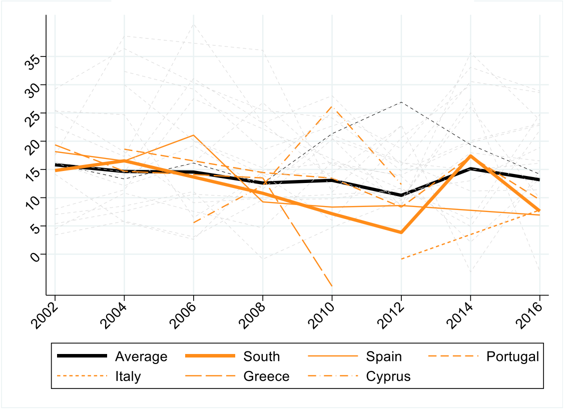

Prevalence Ratio – EU-SILC

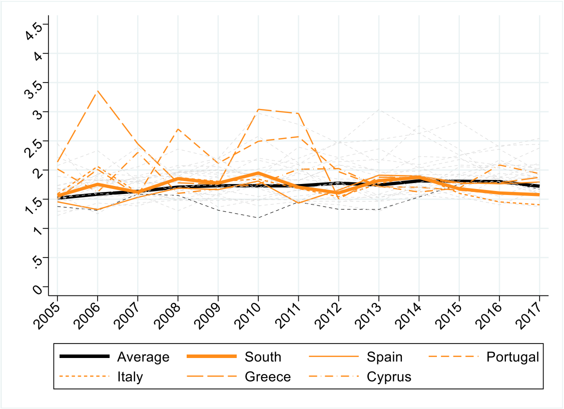

Prevalence Ratio - ESS

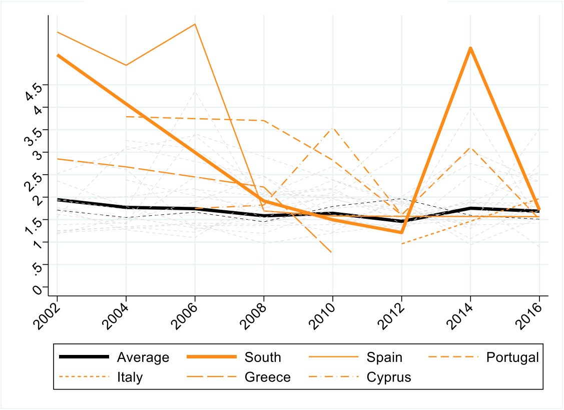

Central East

Men

Low Educated – EU-SILC

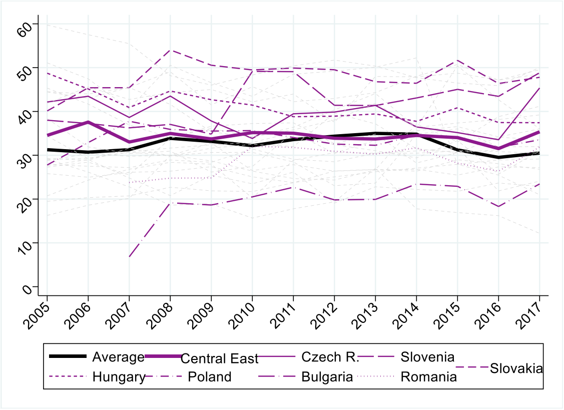

Low Educated - ESS

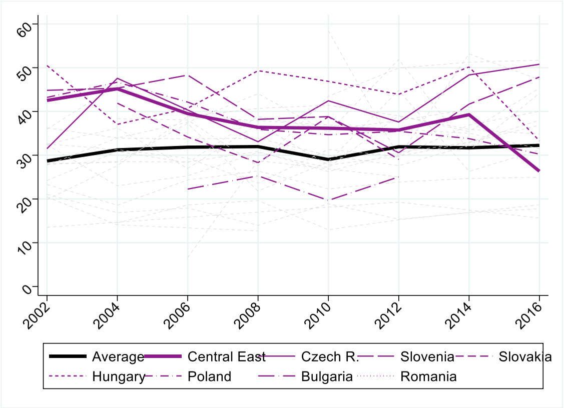

High Educated – EU-SILC

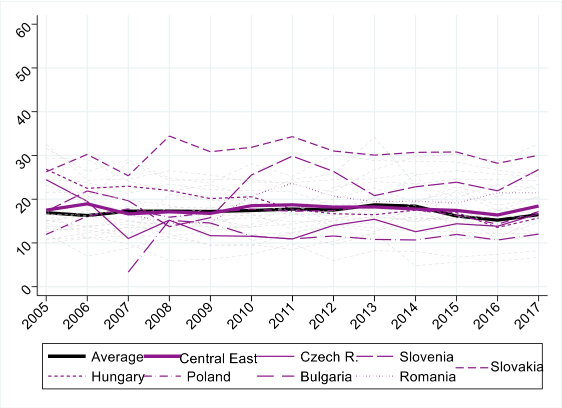

High Educated - ESS

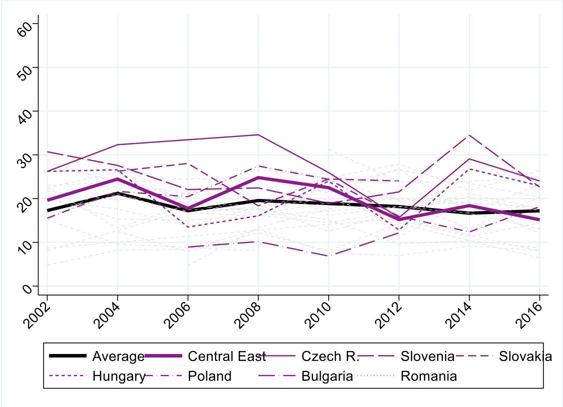

Prevalence Difference – EU-SILC

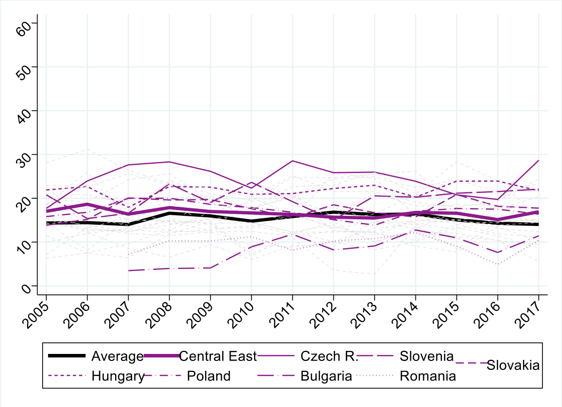

Prevalence Difference - ESS

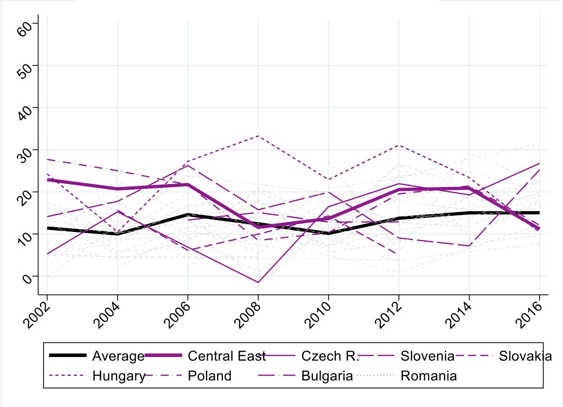

Prevalence Ratio – EU-SILC

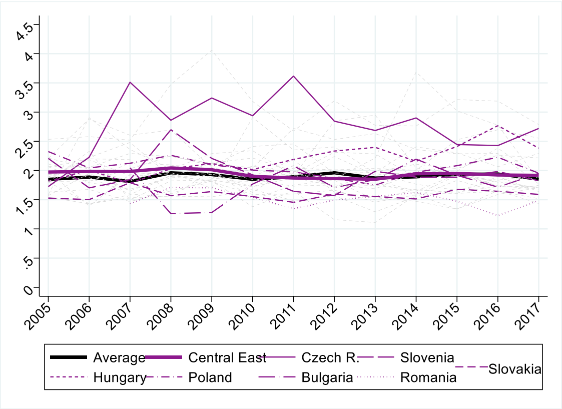

Prevalence Ratio - ESS

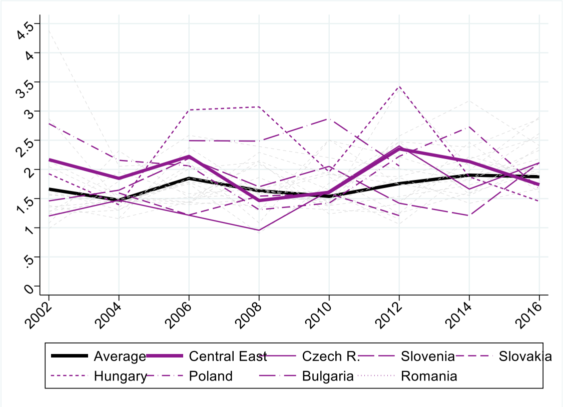

Women

Low Educated – EU-SILC

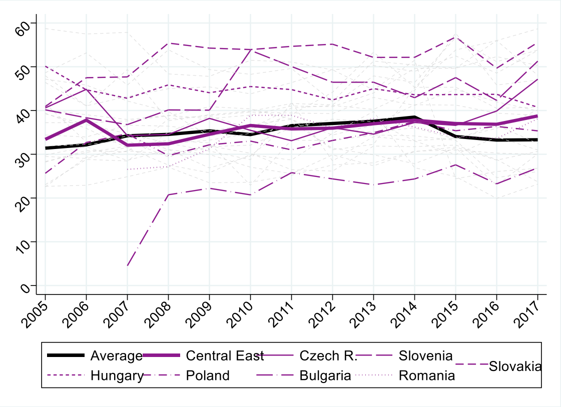

Low Educated - ESS

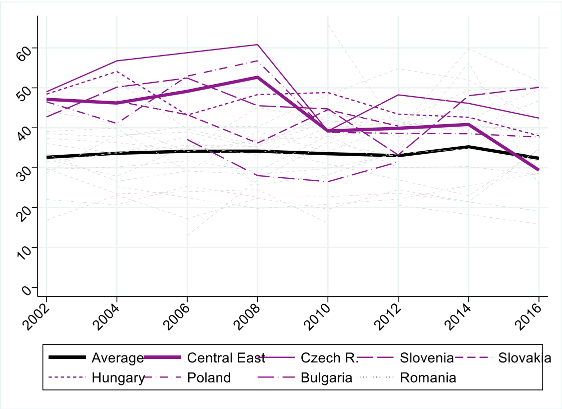

High Educated – EU-SILC

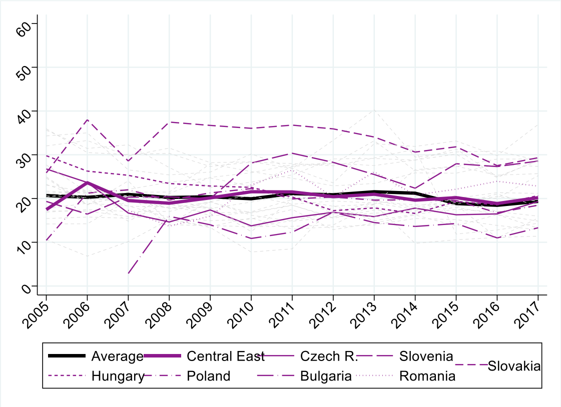

High Educated - ESS

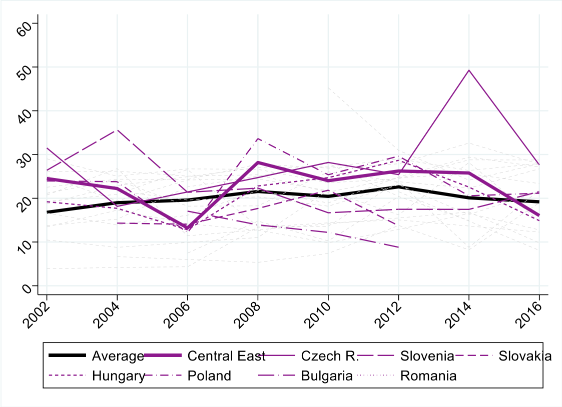

Prevalence Difference – EU-SILC

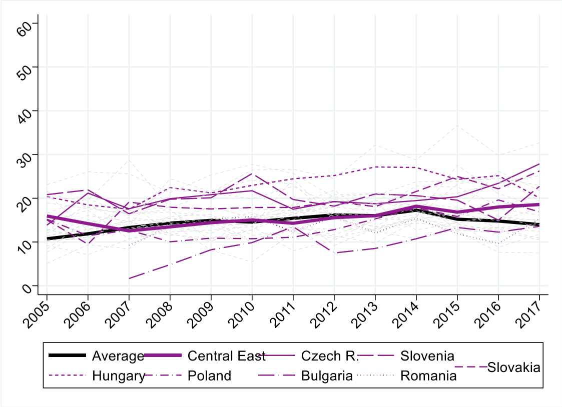

Prevalence Difference - ESS

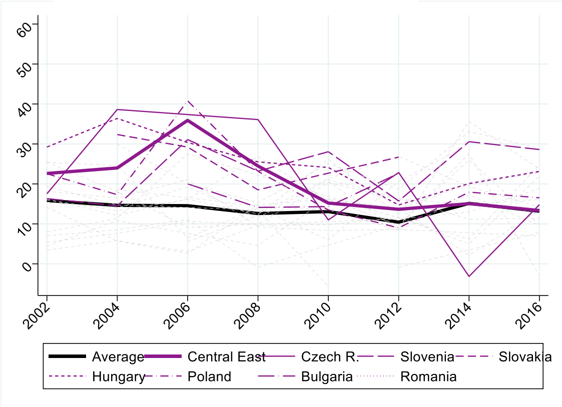

Prevalence Ratio – EU-SILC

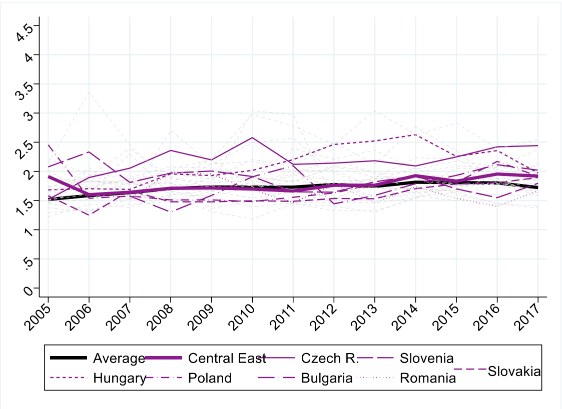

Prevalence Ratio - ESS

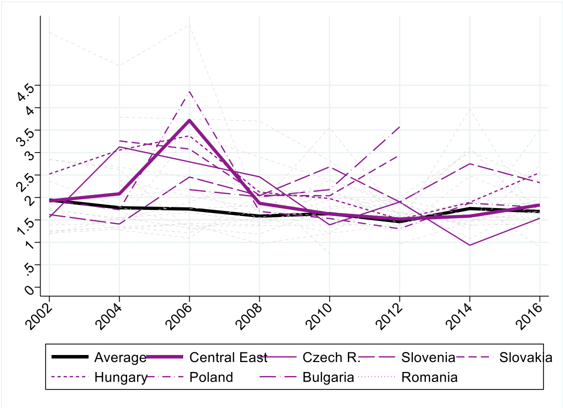

Online Resource 6

Table S6.1 Age standardized prevalence of GALI (Global Activity Limitation Indicator) disability (ages 30-79), prevalence difference(low-high) and prevalence ratio (low/high) for 26 countries and pooled sample, sex and survey (European Union Statistics on Income and Living Conditions 2005-2017; European Social Survey 2002-2016)

EU Statistics on Income and Living Conditions (EU-SILC)

Males

		Age-standardized GALI Prevalence %				Inequality	
Country	Year	Low	Medium	High	Total	Prevalence Difference	Prevalence Ratio
All countries	2005	31.27	23.43	16.92	24.11	14.35	1.82
All countries	2006	30.70	22.00	16.26	23.55	14.44	1.81
All countries	2007	31.28	23.24	17.26	24.77	14.02	1.86
All countries	2008	33.86	24.25	17.27	25.76	16.59	1.99
All countries	2009	33.16	24.63	17.19	25.71	15.97	1.97
All countries	2010	32.19	24.10	17.41	24.93	14.78	1.88
All countries	2011	33.58	24.72	17.75	25.64	15.83	1.92
All countries	2012	34.35	25.40	17.52	26.01	16.84	2.00
All countries	2013	34.99	25.60	18.71	26.27	16.28	1.93
All countries	2014	34.81	25.54	18.40	26.25	16.41	1.94
All countries	2015	31.22	23.14	16.15	23.61	15.07	2.01
All countries	2016	29.51	21.82	15.21	22.08	14.30	2.03
All countries	2017	30.51	22.74	16.47	22.37	14.04	1.91
Austria	2005	44.45	30.48	24.53	31.55	19.92	1.81
Austria	2006	42.99	31.41	23.84	31.70	19.15	1.80
Austria	2007	45.49	30.69	21.28	30.76	24.21	2.14
Austria	2008	49.07	30.98	23.41	31.94	25.65	2.10
Austria	2009	41.50	28.84	22.17	29.28	19.33	1.87
Austria	2010	45.63	29.23	21.98	30.31	23.64	2.08
Austria	2011	42.22	29.85	21.65	29.80	20.57	1.95
Austria	2012	42.98	30.71	18.60	29.44	24.38	2.31
Austria	2013	44.30	30.55	19.39	29.89	24.91	2.28
Austria	2014	48.04	36.11	25.58	34.25	22.46	1.88
Austria	2015	50.73	35.11	26.52	34.25	24.21	1.91
Austria	2016	47.14	36.98	25.68	34.34	21.46	1.84
Austria	2017	48.54	37.12	25.25	34.17	23.28	1.92
Belgium	2005	29.04	23.10	15.04	23.50	14.00	1.93
Belgium	2006	29.31	21.85	16.11	22.74	13.20	1.82
Belgium	2007	30.75	21.77	15.15	22.94	15.60	2.03
Belgium	2008	31.64	20.74	14.06	23.02	17.57	2.25
Belgium	2009	32.38	19.14	15.55	23.03	16.83	2.08
Belgium	2010	32.92	19.67	13.65	22.22	19.27	2.41
Belgium	2011	35.22	22.28	14.35	23.42	20.87	2.45
Belgium	2012	30.43	20.85	15.57	22.05	14.86	1.95
Belgium	2013	33.43	21.49	17.00	22.95	16.43	1.97
Belgium	2014	35.63	20.68	14.82	22.64	20.81	2.40

Country	Year	Low	Medium	High	Total	Prevalence Difference	Prevalence Ratio
Belgium	2015	36.54	22.59	15.88	22.99	20.66	2.30
Belgium	2016	34.19	23.61	16.56	24.20	17.63	2.06
Belgium	2017	36.01	23.24	15.70	23.51	20.31	2.29
Bulgaria	2007	6.81	4.61	3.33	5.21	3.48	2.05
Bulgaria	2008	19.15	16.27	15.17	16.89	3.98	1.26
Bulgaria	2009	18.64	15.43	14.56	16.35	4.09	1.28
Bulgaria	2010	20.50	13.86	11.60	15.81	8.91	1.77
Bulgaria	2011	22.69	15.97	10.91	17.40	11.78	2.08
Bulgaria	2012	19.81	16.80	11.59	17.24	8.22	1.71
Bulgaria	2013	19.92	16.75	10.80	16.93	9.12	1.84
Bulgaria	2014	23.43	16.36	10.68	17.25	12.75	2.19
Bulgaria	2015	22.91	17.24	11.95	17.76	10.96	1.92
Bulgaria	2016	18.31	13.78	10.67	14.50	7.64	1.72
Bulgaria	2017	23.45	15.69	12.02	16.88	11.43	1.95
Croatia	2010	45.52	30.29	20.27	33.05	25.24	2.25
Croatia	2011	40.90	27.88	16.56	28.63	24.34	2.47
Croatia	2012	36.66	22.50	13.04	23.89	23.61	2.81
Croatia	2013	41.30	31.59	23.41	32.31	17.90	1.76
Croatia	2014	41.62	29.28	20.95	30.81	20.67	1.99
Croatia	2015	51.00	36.22	23.57	37.48	27.43	2.16
Croatia	2016	46.76	33.70	21.74	34.26	25.02	2.15
Croatia	2017	47.59	32.43	21.76	33.26	25.83	2.19
Cyprus	2005	38.39	24.86	17.41	28.57	20.99	2.21
Cyprus	2006	31.90	18.81	11.07	23.04	20.84	2.88
Cyprus	2007	30.41	21.53	11.68	23.34	18.73	2.60
Cyprus	2008	30.93	19.57	11.48	23.19	19.44	2.69
Cyprus	2009	26.16	19.62	12.78	21.31	13.38	2.05
Cyprus	2010	27.44	17.32	14.20	20.47	13.24	1.93
Cyprus	2011	35.24	23.92	18.94	27.69	16.30	1.86
Cyprus	2012	32.40	21.06	14.47	23.73	17.93	2.24
Cyprus	2013	31.06	20.65	16.19	23.81	14.86	1.92
Cyprus	2014	29.39	19.14	14.90	21.99	14.49	1.97
Cyprus	2015	33.36	24.36	16.74	24.88	16.62	1.99
Cyprus	2016	25.70	17.18	10.79	18.31	14.91	2.38
Cyprus	2017	30.96	22.46	16.29	22.90	14.67	1.90
Czech R.	2005	42.15	28.94	24.44	29.29	17.71	1.72
Czech R.	2006	43.47	28.67	19.52	28.44	23.95	2.23
Czech R.	2007	38.63	22.66	11.00	22.32	27.63	3.51
Czech R.	2008	43.52	23.53	15.21	23.95	28.31	2.86
Czech R.	2009	37.82	24.03	11.67	23.38	26.15	3.24
Czech R.	2010	33.83	22.99	11.52	22.09	22.31	2.94
Czech R.	2011	39.47	23.29	10.91	22.13	28.55	3.62
Czech R.	2012	39.85	23.16	14.01	22.59	25.84	2.84
Czech R.	2013	41.38	23.16	15.41	22.74	25.97	2.69
Czech R.	2014	36.46	22.49	12.57	21.37	23.89	2.90
Czech R.	2015	35.17	23.38	14.38	22.55	20.79	2.45
Czech R.	2016	33.54	24.36	13.82	23.18	19.72	2.43
Czech R.	2017	45.36	26.07	16.68	25.17	28.68	2.72
Denmark	2005	16.27	10.96	9.97	12.31	6.30	1.63
Denmark	2006	18.60	12.50	11.10	14.14	7.50	1.68

Country	Year	Low	Medium	High	Total	Prevalence Difference	Prevalence Ratio
Denmark	2007	20.19	12.40	13.76	14.99	6.43	1.47
Denmark	2008	29.95	19.16	18.14	22.14	11.81	1.65
Denmark	2009	32.77	21.63	18.02	23.76	14.75	1.82
Denmark	2010	26.49	23.21	19.20	23.12	7.29	1.38
Denmark	2011	27.85	22.16	16.88	22.18	10.97	1.65
Denmark	2012	26.37	33.00	22.76	28.39	3.61	1.16
Denmark	2013	26.71	28.97	24.06	26.68	2.65	1.11
Denmark	2014	37.17	27.28	23.46	27.94	13.72	1.58
Denmark	2015	33.04	29.34	24.61	28.52	8.44	1.34
Denmark	2016	38.81	28.55	24.42	28.55	14.39	1.59
Denmark	2017	41.17	31.43	24.16	29.99	17.01	1.70
Estonia	2005	59.72	42.64	31.61	42.75	28.11	1.89
Estonia	2006	57.52	41.36	26.33	40.38	31.19	2.18
Estonia	2007	55.48	41.84	29.07	40.27	26.41	1.91
Estonia	2008	48.17	38.51	24.82	36.03	23.34	1.94
Estonia	2009	46.01	32.77	23.68	32.03	22.33	1.94
Estonia	2010	48.31	35.06	28.11	34.39	20.21	1.72
Estonia	2011	50.40	37.00	25.25	34.83	25.15	2.00
Estonia	2012	51.73	34.67	30.19	35.46	21.54	1.71
Estonia	2013	50.34	37.04	28.82	36.38	21.51	1.75
Estonia	2014	47.72	36.62	28.64	36.48	19.09	1.67
Estonia	2015	46.48	36.97	28.50	36.25	17.98	1.63
Estonia	2016	45.90	34.08	26.60	34.01	19.30	1.73
Estonia	2017	50.02	36.53	25.54	34.37	24.48	1.96
Finland	2005	45.33	43.65	29.40	40.00	15.93	1.54
Finland	2006	41.69	40.06	29.12	37.21	12.56	1.43
Finland	2007	36.91	32.83	24.10	30.87	12.81	1.53
Finland	2008	34.03	31.21	21.77	29.52	12.25	1.56
Finland	2009	36.12	29.72	23.33	29.68	12.79	1.55
Finland	2010	34.20	30.44	20.42	28.71	13.78	1.67
Finland	2011	36.38	33.42	24.52	30.69	11.85	1.48
Finland	2012	40.35	34.97	25.73	32.61	14.63	1.57
Finland	2013	44.08	41.53	34.13	39.84	9.95	1.29
Finland	2014	36.38	33.46	23.38	30.65	13.00	1.56
Finland	2015	37.36	34.19	20.41	30.20	16.95	1.83
Finland	2016	38.15	30.97	23.31	29.77	14.84	1.64
Finland	2017	39.20	32.46	22.44	30.52	16.76	1.75
France	2005	27.89	22.34	12.58	23.01	15.31	2.22
France	2006	27.49	20.86	13.59	22.39	13.89	2.02
France	2007	27.19	20.86	14.92	22.87	12.27	1.82
France	2008	28.92	22.10	13.95	23.21	14.96	2.07
France	2009	28.40	21.91	14.15	22.79	14.25	2.01
France	2010	31.43	23.69	17.02	24.76	14.40	1.85
France	2011	29.09	24.75	16.79	24.04	12.30	1.73
France	2012	28.51	25.76	16.25	24.59	12.26	1.75
France	2013	30.49	24.07	18.07	24.27	12.41	1.69
France	2014	30.46	23.94	15.47	23.44	14.99	1.97
France	2015	31.02	27.01	15.84	24.85	15.17	1.96
France	2016	30.84	26.08	17.76	24.67	13.09	1.74
France	2017	30.45	25.68	18.11	24.98	12.33	1.68

**Country**	**Year**	**Low**	**Medium**	**High**	**Total**	**Prevalence Difference**	**Prevalence Ratio**
Germany	2005	51.09	40.83	32.54	37.65	18.56	1.57
Germany	2006	47.45	33.61	24.34	32.49	23.11	1.95
Germany	2007	40.58	33.93	24.56	32.03	16.01	1.65
Germany	2008	50.55	38.05	26.49	35.87	24.06	1.91
Germany	2009	46.55	37.36	25.54	34.48	21.00	1.82
Germany	2010	43.44	35.63	23.93	32.92	19.51	1.82
Germany	2011	48.51	36.29	23.78	33.55	24.73	2.04
Germany	2012	49.20	38.57	24.47	35.20	24.73	2.01
Germany	2013	50.10	36.89	24.63	34.32	25.47	2.03
Germany	2014	52.23	38.62	26.06	36.16	26.17	2.00
Germany	2015	27.91	18.82	11.05	17.46	16.86	2.53
Germany	2016	28.90	19.02	10.71	17.57	18.19	2.70
Germany	2017	29.14	19.17	11.41	17.66	17.73	2.55
Greece	2005	19.48	17.31	12.29	18.10	7.19	1.59
Greece	2006	20.33	16.53	7.00	17.48	13.33	2.91
Greece	2007	20.72	15.27	8.78	17.96	11.94	2.36
Greece	2008	22.69	16.48	11.22	19.11	11.47	2.02
Greece	2009	21.87	14.30	9.55	17.52	12.32	2.29
Greece	2010	21.71	18.67	9.28	18.20	12.43	2.34
Greece	2011	23.72	16.69	12.42	18.87	11.30	1.91
Greece	2012	26.41	19.46	13.86	21.38	12.55	1.91
Greece	2013	26.77	18.51	14.63	21.61	12.14	1.83
Greece	2014	26.37	19.80	15.99	22.05	10.38	1.65
Greece	2015	28.96	20.62	14.97	22.39	13.99	1.93
Greece	2016	28.08	20.46	16.18	22.14	11.90	1.74
Greece	2017	26.79	20.07	15.59	21.33	11.20	1.72
Hungary	2005	48.73	36.03	26.83	37.99	21.90	1.82
Hungary	2006	45.14	33.89	22.48	34.45	22.66	2.01
Hungary	2007	40.90	33.57	22.97	33.53	17.93	1.78
Hungary	2008	44.68	33.19	21.99	33.80	22.68	2.03
Hungary	2009	42.67	32.05	20.14	32.14	22.53	2.12
Hungary	2010	41.46	30.59	20.55	31.28	20.90	2.02
Hungary	2011	38.80	29.09	17.72	28.95	21.08	2.19
Hungary	2012	38.92	24.69	16.68	25.90	22.24	2.33
Hungary	2013	39.43	25.98	16.45	26.83	22.97	2.40
Hungary	2014	37.74	26.10	17.49	26.65	20.25	2.16
Hungary	2015	40.85	29.57	16.94	28.48	23.91	2.41
Hungary	2016	37.46	26.39	13.53	25.48	23.93	2.77
Hungary	2017	37.43	25.28	15.71	25.16	21.72	2.38
Ireland	2005	28.80	17.49	15.27	24.03	13.52	1.89
Ireland	2006	30.38	14.26	13.66	23.04	16.72	2.22
Ireland	2007	29.77	16.33	12.00	22.67	17.77	2.48
Ireland	2008	30.02	17.98	15.85	23.31	14.17	1.89
Ireland	2009	28.71	21.25	13.49	23.34	15.22	2.13
Ireland	2010	26.39	16.73	11.92	20.33	14.47	2.21
Ireland	2011	24.68	19.37	12.39	19.81	12.29	1.99
Ireland	2012	24.34	17.70	14.22	19.43	10.12	1.71
Ireland	2013	26.65	17.70	13.46	19.87	13.19	1.98
Ireland	2014	26.79	15.82	12.71	19.14	14.08	2.11
Ireland	2015	25.47	17.91	12.53	18.93	12.94	2.03

Rubio Valverde JR, *et al. J Epidemiol Community Health* 2021; 75:712–720. doi: 10.1136/jech-2020-216141

Country	Year	Low	Medium	High	Total	Prevalence Difference	Prevalence Ratio
Ireland	2016	27.34	13.02	12.08	17.74	15.26	2.26
Ireland	2017	27.19	16.25	12.28	18.30	14.91	2.21
Italy	2005	20.73	14.09	10.75	18.00	9.99	1.93
Italy	2006	24.32	16.01	12.37	21.06	11.95	1.97
Italy	2007	26.81	19.52	13.63	23.91	13.18	1.97
Italy	2008	28.39	20.22	13.57	24.92	14.81	2.09
Italy	2009	27.96	19.72	13.75	24.14	14.22	2.03
Italy	2010	20.25	14.42	13.51	17.77	6.74	1.50
Italy	2011	28.85	19.77	16.48	25.64	12.37	1.75
Italy	2012	30.62	22.65	16.71	27.40	13.91	1.83
Italy	2013	30.46	23.68	20.39	27.35	10.07	1.49
Italy	2014	30.98	22.56	18.44	26.42	12.54	1.68
Italy	2015	31.73	21.48	19.73	26.51	12.00	1.61
Italy	2016	21.86	15.36	11.53	18.37	10.33	1.90
Italy	2017	24.09	17.87	14.69	20.58	9.40	1.64
Latvia	2005	41.84	33.26	23.21	34.42	18.63	1.80
Latvia	2006	45.59	37.67	19.58	36.46	26.01	2.33
Latvia	2007	42.74	34.81	28.51	35.23	14.22	1.50
Latvia	2008	44.32	36.07	24.33	36.07	19.99	1.82
Latvia	2009	45.00	36.17	24.45	35.77	20.55	1.84
Latvia	2010	42.19	33.62	24.54	33.94	17.64	1.72
Latvia	2011	43.98	33.04	23.41	34.05	20.57	1.88
Latvia	2012	37.54	29.80	21.68	30.68	15.86	1.73
Latvia	2013	47.99	37.86	26.79	37.78	21.20	1.79
Latvia	2014	47.90	39.58	29.69	39.37	18.21	1.61
Latvia	2015	48.75	40.59	30.31	39.26	18.44	1.61
Latvia	2016	47.17	38.19	28.67	37.41	18.51	1.65
Latvia	2017	49.52	41.71	32.63	40.77	16.89	1.52
Lithuania	2005	44.51	33.19	21.56	33.23	22.95	2.06
Lithuania	2006	43.98	30.71	21.42	31.00	22.56	2.05
Lithuania	2007	45.45	29.43	19.83	29.33	25.62	2.29
Lithuania	2008	41.61	27.01	18.60	27.58	23.01	2.24
Lithuania	2009	35.33	24.98	16.65	25.30	18.68	2.12
Lithuania	2010	34.15	23.96	16.70	24.22	17.45	2.04
Lithuania	2011	35.80	25.30	13.16	25.47	22.65	2.72
Lithuania	2012	38.06	26.66	15.04	26.82	23.02	2.53
Lithuania	2013	41.86	25.19	15.90	26.21	25.96	2.63
Lithuania	2014	36.36	26.71	15.42	25.64	20.95	2.36
Lithuania	2015	50.41	30.09	22.05	30.03	28.36	2.29
Lithuania	2016	42.66	29.22	18.62	27.96	24.04	2.29
Lithuania	2017	36.06	31.15	20.38	28.78	15.69	1.77
Luxembourg	2005	31.57	22.67	21.98	25.36	9.59	1.44
Luxembourg	2006	31.53	24.19	19.77	26.09	11.76	1.59
Luxembourg	2007	32.54	22.61	15.87	25.53	16.67	2.05
Luxembourg	2008	25.99	20.59	11.31	21.22	14.68	2.30
Luxembourg	2009	28.70	20.30	16.87	22.34	11.83	1.70
Luxembourg	2010	30.25	19.80	15.33	22.55	14.93	1.97
Luxembourg	2011	26.43	18.64	13.62	19.98	12.81	1.94
Luxembourg	2012	28.31	18.37	12.87	20.40	15.45	2.20
Luxembourg	2013	33.61	26.31	16.80	26.52	16.81	2.00

Rubio Valverde JR, *et al. J Epidemiol Community Health* 2021; 75:712–720. doi: 10.1136/jech-2020-216141

Country	Year	Low	Medium	High	Total	Prevalence Difference	Prevalence Ratio
Luxembourg	2014	32.29	25.02	17.10	25.40	15.19	1.89
Luxembourg	2015	32.45	26.64	15.46	25.45	16.99	2.10
Luxembourg	2016	33.91	28.82	20.16	27.93	13.75	1.68
Luxembourg	2017	38.35	30.15	22.99	30.38	15.35	1.67
Malta	2008	14.71	10.07	7.69	13.45	7.02	1.91
Malta	2009	15.07	12.45	9.70	14.07	5.37	1.55
Malta	2010	15.15	12.95	11.16	14.30	3.99	1.36
Malta	2011	15.16	8.17	8.69	13.38	6.47	1.74
Malta	2012	12.58	4.87	5.88	10.77	6.69	2.14
Malta	2013	12.59	6.08	6.28	11.10	6.31	2.00
Malta	2014	11.76	6.93	6.50	10.28	5.26	1.81
Malta	2015	11.59	9.24	6.28	10.31	5.31	1.85
Malta	2016	16.46	10.69	7.65	13.93	8.80	2.15
Malta	2017	14.93	8.82	7.06	12.50	7.87	2.12
Netherlands	2005	27.30	18.73	13.25	19.92	14.05	2.06
Netherlands	2006	28.43	18.63	12.92	19.20	15.51	2.20
Netherlands	2007	25.52	19.44	11.68	19.01	13.84	2.18
Netherlands	2008	33.82	21.77	21.14	24.97	12.68	1.60
Netherlands	2009	36.63	25.66	19.71	26.14	16.92	1.86
Netherlands	2010	37.24	27.25	19.60	27.02	17.64	1.90
Netherlands	2011	37.39	22.32	17.13	23.89	20.26	2.18
Netherlands	2012	32.64	25.01	20.03	25.38	12.61	1.63
Netherlands	2013	34.85	28.73	22.61	27.91	12.24	1.54
Netherlands	2014	28.23	24.52	19.19	23.30	9.04	1.47
Netherlands	2015	30.61	28.71	22.84	26.90	7.77	1.34
Netherlands	2016	34.13	27.20	20.61	26.11	13.52	1.66
Netherlands	2017	33.55	29.00	23.39	27.53	10.16	1.43
Norway	2005	27.60	22.01	10.90	20.26	16.71	2.53
Norway	2006	29.03	20.47	11.25	20.14	17.78	2.58
Norway	2007	24.81	19.63	9.85	18.32	14.96	2.52
Norway	2008	20.79	14.06	5.98	13.13	14.80	3.47
Norway	2009	25.46	15.94	6.27	14.89	19.19	4.06
Norway	2010	23.48	15.81	7.38	14.73	16.10	3.18
Norway	2011	26.15	18.39	9.77	17.35	16.38	2.68
Norway	2012	19.09	13.63	5.98	11.58	13.11	3.19
Norway	2013	22.81	14.80	8.22	14.26	14.58	2.77
Norway	2014	22.20	14.32	8.04	12.68	14.16	2.76
Norway	2015	21.77	12.64	6.77	12.93	15.00	3.21
Norway	2016	23.66	12.22	7.42	13.07	16.24	3.19
Norway	2017	22.90	13.65	8.22	13.73	14.68	2.79
Poland	2005	27.78	20.36	11.95	20.88	15.82	2.32
Poland	2006	32.90	25.70	16.09	25.97	16.81	2.04
Poland	2007	37.77	26.64	17.78	27.65	19.98	2.12
Poland	2008	35.90	24.45	15.88	25.47	20.02	2.26
Poland	2009	35.52	25.87	16.91	26.37	18.61	2.10
Poland	2010	35.63	26.47	17.75	26.94	17.88	2.01
Poland	2011	34.05	25.79	17.23	26.15	16.82	1.98
Poland	2012	32.55	26.09	17.43	25.88	15.11	1.87
Poland	2013	32.27	26.46	18.42	26.26	13.86	1.75
Poland	2014	34.57	25.95	17.55	25.75	17.02	1.97

Rubio Valverde JR, *et al. J Epidemiol Community Health* 2021; 75:712–720. doi: 10.1136/jech-2020-216141

Country	Year	Low	Medium	High	Total	Prevalence Difference	Prevalence Ratio
Poland	2015	33.98	25.97	16.31	25.28	17.67	2.08
Poland	2016	31.80	23.48	14.29	23.07	17.51	2.23
Poland	2017	33.53	24.38	17.15	24.15	16.38	1.95
Portugal	2005	29.29	17.64	14.77	29.23	14.52	1.98
Portugal	2006	28.85	16.93	20.71	28.62	8.14	1.39
Portugal	2007	31.63	15.85	16.35	30.81	15.28	1.93
Portugal	2008	29.89	16.73	15.19	29.58	14.70	1.97
Portugal	2009	30.64	20.16	18.10	30.53	12.54	1.69
Portugal	2010	30.65	15.55	10.54	29.25	20.11	2.91
Portugal	2011	28.47	16.25	12.11	27.54	16.36	2.35
Portugal	2012	23.05	12.53	9.59	21.72	13.46	2.40
Portugal	2013	23.64	15.99	12.10	22.95	11.54	1.95
Portugal	2014	34.79	21.18	19.70	31.41	15.09	1.77
Portugal	2015	36.02	23.78	21.69	32.50	14.33	1.66
Portugal	2016	31.93	24.65	17.41	28.96	14.52	1.83
Portugal	2017	31.98	24.20	16.80	28.86	15.17	1.90
Romania	2007	23.77	18.73	16.58	20.57	7.19	1.43
Romania	2008	24.78	20.85	14.48	21.45	10.30	1.71
Romania	2009	24.82	22.73	14.58	22.68	10.25	1.70
Romania	2010	32.04	27.08	20.81	27.96	11.22	1.54
Romania	2011	31.78	28.16	23.63	28.43	8.15	1.34
Romania	2012	30.92	25.96	20.69	26.99	10.23	1.49
Romania	2013	30.25	25.31	19.42	26.31	10.83	1.56
Romania	2014	31.70	24.89	19.55	25.74	12.15	1.62
Romania	2015	28.19	24.71	19.12	25.30	9.07	1.47
Romania	2016	26.37	23.65	21.47	24.49	4.90	1.23
Romania	2017	31.87	24.00	21.48	25.60	10.39	1.48
Slovakia	2005	40.11	33.22	26.26	32.69	13.85	1.53
Slovakia	2006	45.40	34.70	30.24	34.45	15.17	1.50
Slovakia	2007	45.43	32.38	25.35	31.86	20.08	1.79
Slovakia	2008	54.05	40.01	34.43	39.80	19.61	1.57
Slovakia	2009	50.55	40.99	30.85	39.69	19.70	1.64
Slovakia	2010	49.48	41.22	31.86	40.44	17.62	1.55
Slovakia	2011	49.91	41.21	34.30	40.59	15.61	1.46
Slovakia	2012	49.52	38.34	31.01	37.83	18.51	1.60
Slovakia	2013	46.78	37.29	30.07	36.76	16.71	1.56
Slovakia	2014	46.44	36.38	30.70	36.04	15.74	1.51
Slovakia	2015	51.66	35.91	30.79	36.04	20.87	1.68
Slovakia	2016	46.38	33.62	28.21	33.54	18.17	1.64
Slovakia	2017	47.79	35.01	30.04	35.12	17.75	1.59
Slovenia	2005	38.00	29.08	17.22	30.02	20.78	2.21
Slovenia	2006	37.25	27.52	21.88	29.42	15.37	1.70
Slovenia	2007	36.26	24.60	19.67	26.11	16.59	1.84
Slovenia	2008	37.04	28.25	13.73	27.33	23.32	2.70
Slovenia	2009	34.82	26.36	15.78	26.16	19.05	2.21
Slovenia	2010	49.15	37.20	25.52	37.31	23.63	1.93
Slovenia	2011	49.09	37.44	29.86	38.00	19.23	1.64
Slovenia	2012	41.40	35.09	26.35	34.52	15.05	1.57
Slovenia	2013	41.35	33.63	20.81	32.44	20.54	1.99
Slovenia	2014	43.08	32.62	22.84	31.99	20.24	1.89

Country	Year	Low	Medium	High	Total	Prevalence Difference	Prevalence Ratio
Slovenia	2015	45.04	32.55	23.88	32.27	21.16	1.89
Slovenia	2016	43.45	31.71	21.91	30.95	21.54	1.98
Slovenia	2017	48.84	37.38	26.76	36.73	22.08	1.82
Spain	2005	25.81	17.73	14.32	22.52	11.49	1.80
Spain	2006	24.98	16.80	17.45	22.57	7.53	1.43
Spain	2007	26.10	18.12	16.46	22.94	9.64	1.59
Spain	2008	26.36	18.89	13.73	22.94	12.63	1.92
Spain	2009	27.38	22.73	15.16	24.63	12.22	1.81
Spain	2010	25.07	20.73	15.78	22.88	9.29	1.59
Spain	2011	23.75	18.04	14.87	21.39	8.89	1.60
Spain	2012	25.06	19.43	15.06	22.14	10.00	1.66
Spain	2013	26.24	19.02	18.00	23.38	8.24	1.46
Spain	2014	27.06	19.42	17.06	22.94	10.00	1.59
Spain	2015	27.94	21.74	14.56	23.21	13.38	1.92
Spain	2016	25.46	18.52	14.06	20.96	11.40	1.81
Spain	2017	20.98	14.23	12.32	17.26	8.66	1.70
Sweden	2005	29.12	22.73	13.78	21.73	15.34	2.11
Sweden	2006	26.14	17.86	13.92	18.99	12.22	1.88
Sweden	2007	22.78	17.79	14.26	17.84	8.52	1.60
Sweden	2008	18.60	15.41	12.03	14.92	6.56	1.55
Sweden	2009	18.93	13.43	9.47	13.59	9.46	2.00
Sweden	2010	15.66	12.75	9.65	12.31	6.01	1.62
Sweden	2011	17.78	14.23	8.51	13.12	9.27	2.09
Sweden	2012	19.36	13.53	10.44	13.33	8.92	1.85
Sweden	2013	27.02	19.28	12.21	18.15	14.81	2.21
Sweden	2014	17.78	8.24	4.82	8.66	12.96	3.69
Sweden	2015	16.92	9.06	5.60	9.54	11.32	3.02
Sweden	2016	16.21	9.64	5.84	10.09	10.37	2.78
Sweden	2017	12.17	9.81	6.66	9.31	5.52	1.83
Switzerland	2008	28.06	22.52	16.53	21.09	11.53	1.70
Switzerland	2009	30.45	22.63	17.12	21.59	13.33	1.78
Switzerland	2010	32.51	23.02	17.57	21.97	14.94	1.85
Switzerland	2011	29.76	22.97	17.54	21.55	12.22	1.70
Switzerland	2012	26.05	19.12	14.20	17.97	11.85	1.83
Switzerland	2013	37.97	28.17	24.22	27.44	13.75	1.57
Switzerland	2015	37.95	29.00	22.27	27.30	15.69	1.70
Switzerland	2016	35.22	32.29	23.98	29.24	11.24	1.47
UK	2005	34.38	24.09	16.65	22.63	17.73	2.06
UK	2006	29.20	21.37	16.30	21.42	12.90	1.79
UK	2007	30.74	21.56	18.30	21.88	12.44	1.68
UK	2008	29.84	19.76	15.93	20.85	13.91	1.87
UK	2009	31.62	19.85	16.34	22.09	15.28	1.94
UK	2010	32.50	20.84	16.17	21.68	16.33	2.01
UK	2011	31.99	21.20	16.84	22.00	15.15	1.90
UK	2012	39.62	19.64	13.91	21.78	25.70	2.85
UK	2013	37.16	21.48	12.61	21.65	24.56	2.95
UK	2014	31.47	22.36	15.41	23.56	16.06	2.04
UK	2015	30.62	21.41	15.62	22.84	15.00	1.96
UK	2016	31.23	23.26	17.36	23.54	13.88	1.80
UK	2017	35.12	28.74	20.67	23.70	14.46	1.70

EU Statistics on Income and Living Conditions (EU-SILC)

Females

Country	Year	Low	Medium	High	Total	Prevalence Difference	Prevalence Ratio
All countries	2005	31.39	24.12	20.67	26.45	10.72	1.51
All countries	2006	32.15	24.53	20.28	26.91	11.87	1.57
All countries	2007	34.23	24.87	20.93	27.93	13.29	1.67
All countries	2008	34.54	25.64	20.23	28.41	14.31	1.75
All countries	2009	35.34	26.28	20.40	28.74	14.94	1.76
All countries	2010	34.49	25.60	19.94	27.72	14.55	1.79
All countries	2011	36.53	25.89	21.12	28.63	15.41	1.77
All countries	2012	37.04	25.99	20.86	28.48	16.18	1.80
All countries	2013	37.55	27.58	21.54	29.39	16.01	1.79
All countries	2014	38.49	27.30	21.22	29.37	17.28	1.86
All countries	2015	34.05	24.92	18.84	26.24	15.21	1.84
All countries	2016	33.24	24.00	18.45	24.87	14.79	1.83
All countries	2017	33.31	24.44	19.33	25.08	13.98	1.75
Austria	2005	40.19	29.32	21.25	32.49	18.94	1.89
Austria	2006	39.04	28.82	21.44	30.89	17.60	1.82
Austria	2007	37.95	28.51	21.46	30.92	16.49	1.77
Austria	2008	38.88	31.35	21.47	32.34	17.41	1.81
Austria	2009	38.14	28.99	27.94	31.28	10.20	1.37
Austria	2010	38.96	29.83	26.16	31.71	12.80	1.49
Austria	2011	41.03	29.39	24.63	31.73	16.41	1.67
Austria	2012	40.88	25.99	22.90	29.09	17.98	1.79
Austria	2013	44.01	30.50	23.43	32.50	20.58	1.88
Austria	2014	49.63	32.97	25.86	35.44	23.76	1.92
Austria	2015	49.65	33.94	25.79	35.69	23.86	1.93
Austria	2016	52.32	35.01	27.79	37.13	24.53	1.88
Austria	2017	50.30	35.79	27.34	36.50	22.96	1.84
Belgium	2005	34.40	25.76	23.22	28.72	11.18	1.48
Belgium	2006	33.95	24.56	19.85	27.27	14.10	1.71
Belgium	2007	36.63	25.70	19.53	26.64	17.10	1.88
Belgium	2008	36.43	23.68	18.59	26.04	17.84	1.96
Belgium	2009	35.33	25.39	17.70	26.92	17.63	2.00
Belgium	2010	36.77	25.24	19.19	27.21	17.58	1.92
Belgium	2011	37.97	25.16	20.87	27.46	17.10	1.82
Belgium	2012	34.41	22.51	16.46	23.98	17.94	2.09
Belgium	2013	36.66	24.37	19.37	26.10	17.29	1.89
Belgium	2014	41.31	27.60	18.50	27.09	22.81	2.23
Belgium	2015	41.24	26.75	18.67	26.78	22.58	2.21
Belgium	2016	39.40	27.15	21.04	27.59	18.36	1.87
Belgium	2017	38.92	29.91	18.31	27.46	20.61	2.13
Bulgaria	2007	4.52	2.77	2.87	3.95	1.65	1.58
Bulgaria	2008	20.75	18.56	15.92	18.53	4.82	1.30
Bulgaria	2009	22.22	17.29	13.99	18.26	8.22	1.59
Bulgaria	2010	20.72	15.04	10.87	15.82	9.86	1.91
Bulgaria	2011	25.79	15.77	12.31	17.88	13.49	2.10

Country	Year	Low	Medium	High	Total	Prevalence Difference	Prevalence Ratio
Bulgaria	2012	24.37	16.78	16.88	18.92	7.49	1.44
Bulgaria	2013	23.02	16.82	14.50	18.04	8.52	1.59
Bulgaria	2014	24.36	16.39	13.62	17.65	10.74	1.79
Bulgaria	2015	27.58	17.66	14.28	19.01	13.30	1.93
Bulgaria	2016	23.25	14.97	10.99	15.93	12.25	2.11
Bulgaria	2017	26.89	17.14	13.31	18.32	13.58	2.02
Croatia	2010	38.15	29.59	18.37	31.57	19.79	2.08
Croatia	2011	37.04	24.08	16.85	28.35	20.19	2.20
Croatia	2012	32.40	20.78	12.25	24.36	20.15	2.64
Croatia	2013	38.57	28.37	22.35	31.35	16.22	1.73
Croatia	2014	40.49	28.56	19.93	31.40	20.57	2.03
Croatia	2015	46.64	35.08	25.27	37.58	21.36	1.85
Croatia	2016	42.06	31.89	24.05	34.06	18.00	1.75
Croatia	2017	43.56	33.27	24.95	34.93	18.61	1.75
Cyprus	2005	41.58	23.40	20.61	34.21	20.97	2.02
Cyprus	2006	32.80	18.77	20.23	27.44	12.57	1.62
Cyprus	2007	34.10	19.66	14.82	27.77	19.29	2.30
Cyprus	2008	31.10	18.80	19.44	25.16	11.67	1.60
Cyprus	2009	26.88	22.01	16.02	24.33	10.85	1.68
Cyprus	2010	29.61	25.94	16.83	26.27	12.78	1.76
Cyprus	2011	37.24	27.53	18.50	31.10	18.74	2.01
Cyprus	2012	31.38	23.64	15.49	26.63	15.88	2.03
Cyprus	2013	29.87	23.55	17.35	25.74	12.52	1.72
Cyprus	2014	27.84	19.42	17.15	23.02	10.69	1.62
Cyprus	2015	31.45	21.58	18.59	25.83	12.87	1.69
Cyprus	2016	22.92	15.04	10.98	18.51	11.94	2.09
Cyprus	2017	30.07	19.98	15.52	23.24	14.55	1.94
Czech R.	2005	40.59	28.33	26.75	30.24	13.84	1.52
Czech R.	2006	44.84	28.76	23.66	30.48	21.18	1.89
Czech R.	2007	34.27	23.85	16.68	25.00	17.59	2.05
Czech R.	2008	34.53	23.94	14.64	24.90	19.89	2.36
Czech R.	2009	38.20	24.09	17.39	25.55	20.81	2.20
Czech R.	2010	35.47	22.80	13.76	23.89	21.72	2.58
Czech R.	2011	33.10	24.17	15.61	24.71	17.49	2.12
Czech R.	2012	36.08	23.04	16.85	24.29	19.23	2.14
Czech R.	2013	34.61	23.74	15.86	24.39	18.75	2.18
Czech R.	2014	37.26	22.78	17.80	23.75	19.46	2.09
Czech R.	2015	36.61	24.12	16.28	24.22	20.33	2.25
Czech R.	2016	39.89	23.96	16.48	24.33	23.41	2.42
Czech R.	2017	47.16	27.06	19.31	27.45	27.85	2.44
Denmark	2005	23.05	18.91	15.42	19.12	7.63	1.50
Denmark	2006	28.82	18.41	15.77	21.31	13.05	1.83
Denmark	2007	28.47	19.70	15.49	21.39	12.98	1.84
Denmark	2008	35.07	27.07	21.13	27.62	13.94	1.66
Denmark	2009	38.72	24.86	24.82	28.44	13.90	1.56
Denmark	2010	35.89	26.70	25.90	27.99	9.99	1.39
Denmark	2011	40.58	28.09	27.70	30.29	12.87	1.46
Denmark	2012	41.14	32.34	28.06	31.69	13.08	1.47
Denmark	2013	40.26	31.66	27.54	31.05	12.73	1.46
Denmark	2014	44.56	33.22	28.80	31.58	15.75	1.55

Country	Year	Low	Medium	High	Total	Prevalence Difference	Prevalence Ratio
Denmark	2015	57.67	37.21	30.13	35.62	27.54	1.91
Denmark	2016	45.82	31.14	24.78	30.06	21.04	1.85
Denmark	2017	46.96	34.49	28.78	33.26	18.18	1.63
Estonia	2005	58.72	42.76	35.62	42.55	23.11	1.65
Estonia	2006	57.49	40.77	31.44	39.31	26.04	1.83
Estonia	2007	57.85	38.73	32.28	38.50	25.57	1.79
Estonia	2008	48.36	35.08	27.08	33.52	21.28	1.79
Estonia	2009	47.81	34.56	22.49	31.15	25.32	2.13
Estonia	2010	51.49	36.23	23.74	32.67	27.75	2.17
Estonia	2011	51.07	36.87	25.41	33.40	25.67	2.01
Estonia	2012	49.27	37.37	29.05	34.88	20.22	1.70
Estonia	2013	50.26	38.59	29.15	35.44	21.11	1.72
Estonia	2014	48.95	39.26	28.78	35.47	20.17	1.70
Estonia	2015	52.47	36.98	31.32	35.99	21.15	1.68
Estonia	2016	48.55	35.73	24.98	32.02	23.57	1.94
Estonia	2017	53.73	39.24	28.56	34.80	25.16	1.88
Finland	2005	47.18	45.38	34.31	41.64	12.87	1.38
Finland	2006	45.81	43.45	35.01	40.92	10.80	1.31
Finland	2007	42.17	35.06	26.55	32.91	15.62	1.59
Finland	2008	39.76	34.70	25.39	31.67	14.37	1.57
Finland	2009	34.16	33.84	26.05	31.91	8.11	1.31
Finland	2010	35.64	36.98	30.18	34.48	5.46	1.18
Finland	2011	39.05	36.96	26.87	33.81	12.18	1.45
Finland	2012	44.17	39.64	33.29	37.10	10.88	1.33
Finland	2013	53.33	47.26	40.26	44.21	13.07	1.32
Finland	2014	45.30	35.72	29.46	33.85	15.84	1.54
Finland	2015	53.83	39.94	30.64	36.26	23.18	1.76
Finland	2016	55.97	37.83	30.84	35.29	25.13	1.81
Finland	2017	48.07	40.29	28.76	35.76	19.30	1.67
France	2005	28.36	22.06	16.88	24.13	11.48	1.68
France	2006	30.42	23.05	17.44	25.26	12.98	1.74
France	2007	31.84	21.17	17.06	25.07	14.78	1.87
France	2008	32.42	21.72	17.03	25.01	15.39	1.90
France	2009	33.22	24.05	19.33	26.21	13.89	1.72
France	2010	34.79	23.97	20.63	27.04	14.15	1.69
France	2011	36.24	23.74	18.69	26.48	17.55	1.94
France	2012	35.85	24.03	19.44	26.30	16.41	1.84
France	2013	35.09	25.49	18.97	26.74	16.12	1.85
France	2014	36.01	25.75	17.53	26.58	18.49	2.05
France	2015	33.47	24.93	19.09	26.38	14.37	1.75
France	2016	36.67	25.16	20.47	26.91	16.20	1.79
France	2017	32.46	25.70	19.37	25.98	13.08	1.68
Germany	2005	46.41	39.43	35.93	39.45	10.48	1.29
Germany	2006	41.06	34.45	30.50	35.26	10.56	1.35
Germany	2007	43.20	33.50	30.06	35.15	13.13	1.44
Germany	2008	44.98	34.73	30.33	36.18	14.66	1.48
Germany	2009	45.40	34.75	27.32	35.60	18.08	1.66
Germany	2010	43.81	33.44	28.34	34.46	15.48	1.55
Germany	2011	46.38	32.05	27.98	34.16	18.39	1.66
Germany	2012	49.29	34.18	27.47	35.65	21.83	1.79

Country	Year	Low	Medium	High	Total	Prevalence Difference	Prevalence Ratio
Germany	2013	44.71	36.17	29.26	36.27	15.45	1.53
Germany	2014	51.49	36.75	31.29	37.99	20.21	1.65
Germany	2015	24.39	16.76	11.87	17.03	12.52	2.06
Germany	2016	26.06	16.92	12.79	17.47	13.27	2.04
Germany	2017	26.80	17.73	14.37	18.41	12.44	1.87
Greece	2005	22.67	15.23	10.61	20.36	12.06	2.14
Greece	2006	22.91	12.36	6.82	19.48	16.09	3.36
Greece	2007	24.86	12.20	10.16	20.63	14.71	2.45
Greece	2008	27.06	17.01	15.19	22.94	11.86	1.78
Greece	2009	23.48	17.46	13.37	20.24	10.12	1.76
Greece	2010	23.60	14.37	7.76	19.41	15.84	3.04
Greece	2011	25.23	17.28	8.50	21.31	16.73	2.97
Greece	2012	27.57	19.57	18.34	24.32	9.23	1.50
Greece	2013	28.04	20.43	14.94	24.13	13.10	1.88
Greece	2014	30.70	21.19	16.40	24.81	14.30	1.87
Greece	2015	30.97	21.05	17.51	25.29	13.46	1.77
Greece	2016	30.28	20.85	16.99	24.52	13.29	1.78
Greece	2017	29.96	20.39	15.97	23.65	13.99	1.88
Hungary	2005	50.13	35.59	29.78	40.35	20.35	1.68
Hungary	2006	44.73	33.68	26.26	35.91	18.47	1.70
Hungary	2007	42.79	31.70	25.31	34.47	17.48	1.69
Hungary	2008	45.87	31.34	23.44	34.52	22.43	1.96
Hungary	2009	44.07	31.54	22.86	33.66	21.21	1.93
Hungary	2010	45.48	29.46	22.55	32.89	22.94	2.02
Hungary	2011	44.78	28.48	20.34	31.50	24.44	2.20
Hungary	2012	42.42	26.17	17.23	29.05	25.18	2.46
Hungary	2013	45.00	27.37	17.85	30.06	27.15	2.52
Hungary	2014	43.60	27.30	16.58	29.19	27.02	2.63
Hungary	2015	43.64	28.17	19.34	29.74	24.30	2.26
Hungary	2016	43.70	28.19	18.54	29.69	25.16	2.36
Hungary	2017	40.75	25.51	20.68	27.96	20.08	1.97
Ireland	2005	29.83	21.34	17.17	24.81	12.67	1.74
Ireland	2006	31.71	20.86	17.12	25.28	14.59	1.85
Ireland	2007	28.60	16.48	15.91	22.45	12.69	1.80
Ireland	2008	31.08	17.65	15.22	23.98	15.86	2.04
Ireland	2009	29.94	19.71	13.60	23.26	16.34	2.20
Ireland	2010	27.05	15.95	15.54	20.75	11.51	1.74
Ireland	2011	25.55	14.23	13.02	18.74	12.53	1.96
Ireland	2012	29.86	14.14	13.54	19.28	16.32	2.21
Ireland	2013	27.38	17.14	13.97	19.71	13.41	1.96
Ireland	2014	30.59	18.47	15.44	20.99	15.15	1.98
Ireland	2015	31.65	17.81	15.18	20.18	16.47	2.08
Ireland	2016	28.66	16.73	13.85	18.76	14.80	2.07
Ireland	2017	30.65	17.94	12.88	19.30	17.77	2.38
Italy	2005	22.56	15.54	14.18	20.39	8.38	1.59
Italy	2006	29.32	19.54	14.22	25.73	15.10	2.06
Italy	2007	31.58	23.40	19.58	29.28	12.00	1.61
Italy	2008	33.22	24.82	17.75	29.99	15.47	1.87
Italy	2009	32.57	23.02	18.02	28.74	14.54	1.81
Italy	2010	22.92	18.34	12.43	21.09	10.49	1.84

Country	Year	Low	Medium	High	Total	Prevalence Difference	Prevalence Ratio
Italy	2011	33.24	23.60	19.75	29.89	13.49	1.68
Italy	2012	33.12	25.93	21.47	30.37	11.66	1.54
Italy	2013	34.43	27.72	19.49	30.72	14.94	1.77
Italy	2014	34.19	24.77	17.96	29.17	16.24	1.90
Italy	2015	33.20	24.72	20.75	28.84	12.46	1.60
Italy	2016	24.66	18.20	16.96	21.57	7.70	1.45
Italy	2017	26.04	19.27	18.54	22.97	7.50	1.40
Latvia	2005	48.62	36.58	32.03	38.14	16.59	1.52
Latvia	2006	53.18	41.14	33.25	40.68	19.92	1.60
Latvia	2007	45.82	38.53	29.70	38.18	16.11	1.54
Latvia	2008	53.87	38.38	31.53	38.81	22.34	1.71
Latvia	2009	49.34	35.98	28.20	36.06	21.15	1.75
Latvia	2010	48.27	35.84	28.39	35.38	19.88	1.70
Latvia	2011	49.60	37.13	26.91	35.99	22.69	1.84
Latvia	2012	42.07	31.74	23.57	31.29	18.50	1.78
Latvia	2013	51.30	42.41	32.53	40.85	18.76	1.58
Latvia	2014	50.38	41.37	31.91	39.60	18.47	1.58
Latvia	2015	49.68	43.47	33.15	40.62	16.52	1.50
Latvia	2016	55.99	41.34	29.78	38.81	26.21	1.88
Latvia	2017	58.67	45.08	36.92	43.52	21.76	1.59
Lithuania	2005	47.94	39.63	27.68	38.67	20.26	1.73
Lithuania	2006	41.06	37.20	21.48	35.23	19.58	1.91
Lithuania	2007	49.35	33.26	20.65	32.19	28.71	2.39
Lithuania	2008	37.65	32.97	19.87	30.66	17.79	1.90
Lithuania	2009	39.05	28.78	17.76	27.53	21.29	2.20
Lithuania	2010	39.87	27.24	13.41	25.12	26.46	2.97
Lithuania	2011	41.59	29.64	14.95	26.63	26.65	2.78
Lithuania	2012	41.30	28.28	16.80	26.58	24.51	2.46
Lithuania	2013	47.90	29.88	15.76	26.75	32.15	3.04
Lithuania	2014	46.42	29.21	17.66	27.30	28.76	2.63
Lithuania	2015	56.65	33.44	20.04	31.26	36.61	2.83
Lithuania	2016	51.15	33.70	21.34	30.89	29.80	2.40
Lithuania	2017	53.82	33.15	21.18	30.62	32.64	2.54
Luxembourg	2005	31.10	23.21	18.37	27.16	12.73	1.69
Luxembourg	2006	34.33	25.49	21.66	29.24	12.69	1.59
Luxembourg	2007	29.40	24.21	24.12	26.18	5.26	1.22
Luxembourg	2008	29.57	23.83	19.73	25.75	9.84	1.50
Luxembourg	2009	28.50	22.07	14.51	24.56	13.99	1.96
Luxembourg	2010	25.58	22.20	18.66	23.82	6.92	1.37
Luxembourg	2011	27.45	20.32	12.78	22.76	14.67	2.15
Luxembourg	2012	26.74	23.41	13.72	23.77	13.02	1.95
Luxembourg	2013	34.00	26.12	15.31	27.91	18.68	2.22
Luxembourg	2014	36.17	25.69	17.49	28.56	18.68	2.07
Luxembourg	2015	38.27	30.20	21.58	32.84	16.69	1.77
Luxembourg	2016	41.30	32.61	26.38	34.47	14.91	1.57
Luxembourg	2017	40.97	30.36	27.12	32.86	13.85	1.51
Malta	2008	13.35	6.42	7.80	13.46	5.55	1.71
Malta	2009	15.71	13.91	8.77	15.64	6.94	1.79
Malta	2010	16.84	11.53	8.26	16.32	8.59	2.04
Malta	2011	15.24	9.30	12.05	14.67	3.19	1.26

Country	Year	Low	Medium	High	Total	Prevalence Difference	Prevalence Ratio
Malta	2012	13.78	10.32	10.13	12.99	3.66	1.36
Malta	2013	13.57	12.52	9.27	13.08	4.31	1.47
Malta	2014	11.93	11.01	7.84	11.36	4.09	1.52
Malta	2015	12.69	6.70	6.61	11.18	6.08	1.92
Malta	2016	15.39	10.74	9.76	13.75	5.63	1.58
Malta	2017	16.35	12.19	11.47	14.52	4.88	1.43
Netherlands	2005	28.19	26.11	23.07	26.72	5.12	1.22
Netherlands	2006	30.85	28.15	21.49	27.37	9.36	1.44
Netherlands	2007	30.56	23.83	22.09	25.46	8.47	1.38
Netherlands	2008	38.06	31.37	23.72	32.38	14.35	1.60
Netherlands	2009	38.84	30.97	24.68	32.09	14.15	1.57
Netherlands	2010	39.63	29.58	22.81	31.22	16.82	1.74
Netherlands	2011	39.44	35.04	27.36	33.74	12.07	1.44
Netherlands	2012	39.30	34.11	27.94	33.89	11.35	1.41
Netherlands	2013	42.99	37.96	33.20	37.50	9.79	1.29
Netherlands	2014	41.17	37.19	26.35	34.55	14.82	1.56
Netherlands	2015	44.82	39.80	27.30	35.84	17.51	1.64
Netherlands	2016	41.98	35.12	29.03	34.59	12.95	1.45
Netherlands	2017	39.90	38.09	29.03	35.43	10.87	1.37
Norway	2005	39.25	25.52	17.68	26.70	21.57	2.22
Norway	2006	36.79	23.85	18.91	26.43	17.88	1.95
Norway	2007	34.72	23.16	18.30	24.54	16.42	1.90
Norway	2008	32.53	19.34	15.66	21.09	16.87	2.08
Norway	2009	30.73	20.31	14.94	21.15	15.79	2.06
Norway	2010	28.76	21.28	15.31	20.40	13.44	1.88
Norway	2011	41.88	24.75	17.50	25.68	24.38	2.39
Norway	2012	28.48	17.82	12.87	17.21	15.60	2.21
Norway	2013	34.04	23.63	14.40	22.14	19.64	2.36
Norway	2014	33.02	23.67	16.24	21.49	16.78	2.03
Norway	2015	29.94	21.62	13.74	19.82	16.20	2.18
Norway	2016	33.95	23.52	16.32	22.04	17.63	2.08
Norway	2017	30.98	23.22	14.83	20.95	16.14	2.09
Poland	2005	25.65	18.72	10.45	19.40	15.20	2.45
Poland	2006	32.69	25.30	21.24	25.96	11.44	1.54
Poland	2007	34.68	27.28	21.98	27.93	12.70	1.58
Poland	2008	29.69	25.78	19.64	25.89	10.05	1.51
Poland	2009	32.17	26.42	21.29	27.20	10.88	1.51
Poland	2010	33.01	27.65	22.26	27.69	10.74	1.48
Poland	2011	31.01	25.78	19.93	25.87	11.08	1.56
Poland	2012	33.15	26.08	20.33	26.51	12.82	1.63
Poland	2013	34.92	26.61	19.64	26.73	15.28	1.78
Poland	2014	37.49	26.95	19.71	26.97	17.78	1.90
Poland	2015	35.37	27.29	19.55	26.74	15.83	1.81
Poland	2016	36.36	24.78	16.75	24.44	19.62	2.17
Poland	2017	35.35	24.83	18.47	24.99	16.88	1.91
Portugal	2005	37.22	26.06	24.67	37.79	12.54	1.51
Portugal	2006	37.20	25.50	18.50	36.86	18.70	2.01
Portugal	2007	37.63	25.67	24.02	37.22	13.61	1.57
Portugal	2008	38.78	25.49	14.35	36.88	24.43	2.70
Portugal	2009	40.86	27.53	19.31	39.18	21.55	2.12

Country	Year	Low	Medium	High	Total	Prevalence Difference	Prevalence Ratio
Portugal	2010	40.37	26.81	16.19	38.37	24.18	2.49
Portugal	2011	38.05	21.19	14.80	35.48	23.25	2.57
Portugal	2012	31.01	19.10	15.73	29.59	15.29	1.97
Portugal	2013	32.27	25.23	18.81	30.23	13.46	1.72
Portugal	2014	45.31	27.89	26.59	40.87	18.72	1.70
Portugal	2015	45.32	35.29	27.39	40.91	17.94	1.66
Portugal	2016	40.50	32.95	25.34	36.50	15.16	1.60
Portugal	2017	40.75	30.99	25.68	36.87	15.07	1.59
Romania	2007	26.55	23.66	17.30	24.81	9.25	1.53
Romania	2008	27.18	23.49	13.69	24.98	13.49	1.99
Romania	2009	31.25	25.14	15.98	27.55	15.27	1.96
Romania	2010	38.89	33.28	23.11	35.24	15.78	1.68
Romania	2011	38.90	34.79	26.51	36.08	12.39	1.47
Romania	2012	36.05	34.18	20.32	34.55	15.74	1.77
Romania	2013	38.17	32.37	26.09	34.48	12.08	1.46
Romania	2014	36.20	31.84	20.81	32.53	15.39	1.74
Romania	2015	34.02	30.27	22.13	31.29	11.90	1.54
Romania	2016	33.59	30.89	23.94	31.54	9.65	1.40
Romania	2017	38.14	31.30	22.84	33.03	15.30	1.67
Slovakia	2005	40.91	34.69	25.87	35.03	15.04	1.58
Slovakia	2006	47.50	38.28	38.00	39.77	9.49	1.25
Slovakia	2007	47.68	35.51	28.56	36.43	19.12	1.67
Slovakia	2008	55.41	43.01	37.47	43.79	17.94	1.48
Slovakia	2009	54.26	43.07	36.71	43.38	17.55	1.48
Slovakia	2010	53.93	43.50	36.05	43.97	17.88	1.50
Slovakia	2011	54.66	43.24	36.77	43.75	17.89	1.49
Slovakia	2012	55.15	41.61	35.92	42.14	19.24	1.54
Slovakia	2013	52.14	39.69	34.06	40.50	18.08	1.53
Slovakia	2014	52.16	38.83	30.61	38.84	21.55	1.70
Slovakia	2015	56.79	37.88	31.82	38.88	24.97	1.78
Slovakia	2016	49.67	35.12	27.51	35.67	22.16	1.81
Slovakia	2017	55.55	38.78	29.32	38.72	26.23	1.89
Slovenia	2005	40.16	27.31	19.33	31.66	20.82	2.08
Slovenia	2006	38.34	25.39	16.43	28.80	21.91	2.33
Slovenia	2007	36.78	26.53	20.32	28.24	16.46	1.81
Slovenia	2008	40.14	27.38	20.37	29.47	19.77	1.97
Slovenia	2009	40.08	28.07	19.98	29.86	20.10	2.01
Slovenia	2010	53.75	38.53	28.11	40.18	25.64	1.91
Slovenia	2011	50.06	38.52	30.34	40.15	19.71	1.65
Slovenia	2012	46.46	37.54	28.27	37.76	18.20	1.64
Slovenia	2013	46.47	31.88	25.50	33.00	20.97	1.82
Slovenia	2014	42.93	32.76	22.35	32.23	20.58	1.92
Slovenia	2015	47.52	36.00	27.95	35.80	19.57	1.70
Slovenia	2016	42.31	36.14	27.30	35.26	15.01	1.55
Slovenia	2017	51.28	39.78	28.56	38.83	22.71	1.80
Spain	2005	29.49	21.34	20.26	28.31	9.23	1.46
Spain	2006	29.06	25.05	21.96	28.30	7.09	1.32
Spain	2007	30.78	21.95	20.09	28.57	10.70	1.53
Spain	2008	30.05	20.40	17.77	27.88	12.27	1.69
Spain	2009	31.90	23.33	19.17	29.54	12.73	1.66

Rubio Valverde JR, *et al. J Epidemiol Community Health* 2021; 75:712–720. doi: 10.1136/jech-2020-216141

Country	Year	Low	Medium	High	Total	Prevalence Difference	Prevalence Ratio
Spain	2010	30.23	20.89	16.69	27.32	13.54	1.81
Spain	2011	26.47	18.36	18.46	24.47	8.01	1.43
Spain	2012	26.15	17.71	15.77	23.44	10.39	1.66
Spain	2013	31.87	19.54	16.68	27.42	15.20	1.91
Spain	2014	31.73	20.76	16.72	26.02	15.01	1.90
Spain	2015	32.10	22.93	17.99	26.72	14.12	1.78
Spain	2016	28.80	22.47	16.32	23.91	12.48	1.76
Spain	2017	23.71	15.87	13.22	19.39	10.49	1.79
Sweden	2005	39.37	28.87	24.95	27.73	14.41	1.58
Sweden	2006	35.92	24.14	18.66	23.86	17.26	1.93
Sweden	2007	31.44	24.93	20.77	24.07	10.67	1.51
Sweden	2008	27.54	22.02	14.01	20.48	13.53	1.97
Sweden	2009	25.77	22.16	13.80	19.31	11.97	1.87
Sweden	2010	29.92	19.78	13.58	18.56	16.35	2.20
Sweden	2011	28.68	21.76	13.66	19.12	15.01	2.10
Sweden	2012	34.78	20.54	13.95	18.73	20.83	2.49
Sweden	2013	39.77	25.46	18.71	23.25	21.06	2.13
Sweden	2014	26.91	15.77	9.76	14.47	17.16	2.76
Sweden	2015	24.90	17.59	10.57	16.42	14.34	2.36
Sweden	2016	19.91	16.00	11.10	14.95	8.81	1.79
Sweden	2017	23.17	17.25	11.40	15.59	11.77	2.03
Switzerland	2008	31.54	26.73	24.39	27.46	7.16	1.29
Switzerland	2009	34.82	27.19	24.78	28.29	10.04	1.41
Switzerland	2010	32.29	26.58	26.21	27.53	6.09	1.23
Switzerland	2011	33.40	24.80	23.23	26.16	10.18	1.44
Switzerland	2012	27.21	21.35	21.11	22.65	6.09	1.29
Switzerland	2013	40.18	33.53	32.72	34.37	7.46	1.23
Switzerland	2015	39.23	34.65	31.58	34.51	7.65	1.24
Switzerland	2016	42.25	35.94	32.46	35.60	9.79	1.30
UK	2005	31.85	22.98	21.69	24.35	10.17	1.47
UK	2006	32.24	22.89	21.19	25.06	11.06	1.52
UK	2007	33.10	21.32	18.23	22.93	14.87	1.82
UK	2008	29.39	21.04	18.05	22.29	11.34	1.63
UK	2009	31.75	22.50	16.68	23.64	15.07	1.90
UK	2010	32.08	23.28	16.97	23.14	15.11	1.89
UK	2011	36.07	23.59	19.77	24.86	16.30	1.82
UK	2012	38.03	21.79	18.23	24.80	19.80	2.09
UK	2013	37.43	22.13	18.70	23.86	18.73	2.00
UK	2014	33.11	22.12	20.38	25.62	12.73	1.62
UK	2015	33.66	24.77	19.22	26.18	14.44	1.75
UK	2016	35.93	27.85	20.11	26.91	15.82	1.79
UK	2017	40.13	30.50	25.22	27.98	14.90	1.59

Rubio Valverde JR, *et al. J Epidemiol Community Health* 2021; 75:712–720. doi: 10.1136/jech-2020-216141

European Social Survey (ESS)

Males

Country	Year	Low	Medium	High	Total	Prevalence Difference	Prevalence Ratio
All countries	2002	25.28	27.19	19.01	24.70	6.27	1.33
All countries	2004	28.38	27.18	21.43	26.45	6.95	1.32
All countries	2006	30.03	27.67	17.84	25.97	12.19	1.68
All countries	2008	27.35	27.22	18.47	24.92	8.88	1.48
All countries	2010	26.42	26.27	18.88	25.19	7.54	1.40
All countries	2012	27.26	27.52	19.55	25.33	7.72	1.39
All countries	2014	28.63	28.37	16.87	26.48	11.76	1.70
All countries	2016	27.04	26.16	18.21	24.57	8.83	1.49
Austria	2002	19.89	27.59	20.14	24.42	-0.25	0.99
Austria	2004	27.61	20.96	15.33	22.53	12.27	1.80
Austria	2006	38.86	30.56	15.04	28.50	23.82	2.58
Austria	2014	35.02	21.69	19.26	23.88	15.76	1.82
Austria	2016	31.52	19.53	12.04	18.84	19.47	2.62
Belgium	2002	32.38	21.00	19.84	26.06	12.53	1.63
Belgium	2004	23.01	27.55	17.61	22.32	5.40	1.31
Belgium	2006	25.31	19.87	13.68	20.22	11.62	1.85
Belgium	2008	30.63	23.60	18.18	24.77	12.45	1.68
Belgium	2010	27.18	28.27	19.87	25.13	7.31	1.37
Belgium	2012	39.04	21.14	15.17	24.43	23.86	2.57
Belgium	2014	35.91	22.73	11.31	27.05	24.61	3.18
Belgium	2016	34.84	27.72	14.46	26.84	20.37	2.41
Bulgaria	2006	22.26	20.81	8.93	18.86	13.33	2.49
Bulgaria	2008	25.28	14.82	10.17	17.54	15.11	2.49
Bulgaria	2010	19.67	11.60	6.85	13.01	12.82	2.87
Bulgaria	2012	25.05	14.33	12.16	16.62	12.88	2.06
Cyprus	2006	6.60	4.63	4.65	7.91	1.95	1.42
Cyprus	2008	28.75	12.48	13.38	17.83	15.38	2.15
Cyprus	2010	19.46	17.55	2.03	15.89	17.42	9.57
Cyprus	2012	25.04	13.53	21.80	19.39	3.25	1.15
Czech R.	2002	31.50	36.01	26.22	34.93	5.29	1.20
Czech R.	2004	47.59	33.40	32.32	34.18	15.27	1.47
Czech R.	2008	33.09	32.93	34.58	32.41	-1.49	0.96
Czech R.	2010	42.44	32.42	25.95	32.53	16.49	1.64
Czech R.	2012	37.59	23.72	15.65	23.76	21.94	2.40
Czech R.	2014	48.35	28.68	29.07	30.54	19.28	1.66
Czech R.	2016	50.79	30.98	24.03	29.51	26.76	2.11
Denmark	2002	24.21	27.30	22.50	24.66	1.72	1.08
Denmark	2004	30.88	22.58	13.31	22.02	17.57	2.32
Denmark	2006	28.28	23.86	18.64	23.69	9.63	1.52
Denmark	2008	38.86	22.94	18.59	25.72	20.27	2.09
Denmark	2010	35.87	22.39	25.13	25.43	10.75	1.43
Denmark	2012	37.90	21.61	11.24	22.59	26.66	3.37
Denmark	2014	40.48	22.46	19.77	26.43	20.71	2.05
Estonia	2004	38.33	32.13	28.28	32.89	10.05	1.36
Estonia	2006	38.06	29.72	18.35	28.82	19.70	2.07
Estonia	2008	27.60	28.21	17.47	26.48	10.14	1.58
Estonia	2010	43.47	30.79	22.71	29.54	20.76	1.91

Country	Year	Low	Medium	High	Total	Prevalence Difference	Prevalence Ratio
Estonia	2012	49.92	27.24	26.60	29.57	23.32	1.88
Estonia	2014	51.26	28.60	23.51	29.10	27.76	2.18
Estonia	2016	51.73	36.40	20.26	34.56	31.47	2.55
Finland	2002	35.96	24.56	28.45	31.21	7.51	1.26
Finland	2004	46.07	27.49	23.59	33.27	22.48	1.95
Finland	2006	38.81	30.45	24.51	32.00	14.30	1.58
Finland	2008	40.71	38.77	24.50	34.55	16.20	1.66
Finland	2010	41.45	40.45	21.24	36.24	20.21	1.95
Finland	2012	38.99	33.70	26.80	33.16	12.19	1.45
Finland	2014	35.41	31.60	16.65	30.40	18.76	2.13
Finland	2016	45.33	32.40	23.24	32.91	22.09	1.95
France	2002	27.93	20.17	20.20	23.05	7.73	1.38
France	2004	28.51	20.42	24.69	24.12	3.82	1.15
France	2006	28.47	25.40	19.73	26.06	8.74	1.44
France	2008	30.85	25.31	16.58	26.07	14.27	1.86
France	2010	26.25	18.42	19.02	21.48	7.24	1.38
France	2012	30.62	28.34	19.93	26.28	10.70	1.54
France	2014	32.96	21.02	19.06	25.03	13.90	1.73
France	2016	37.97	27.31	17.93	28.37	20.04	2.12
Germany	2002	36.25	30.45	22.96	30.20	13.29	1.58
Germany	2004	33.94	30.81	23.79	29.27	10.15	1.43
Germany	2006	37.53	30.18	21.05	29.12	16.48	1.78
Germany	2008	44.07	30.76	26.55	30.83	17.52	1.66
Germany	2010	35.21	32.84	24.74	31.79	10.47	1.42
Germany	2012	51.91	36.57	27.89	35.59	24.01	1.86
Germany	2014	31.07	34.40	21.90	31.45	9.17	1.42
Germany	2016	44.16	34.01	26.42	32.64	17.74	1.67
Greece	2002	20.47	17.80	15.11	18.71	5.36	1.35
Greece	2004	14.08	9.16	9.63	12.63	4.45	1.46
Greece	2008	12.71	14.06	8.06	12.76	4.65	1.58
Greece	2010	13.85	8.04	2.17	11.24	11.68	6.39
Hungary	2002	50.50	32.79	26.23	38.46	24.27	1.93
Hungary	2004	37.05	32.08	26.58	32.29	10.47	1.39
Hungary	2006	40.74	35.51	13.49	34.30	27.25	3.02
Hungary	2008	49.32	31.73	16.05	35.35	33.27	3.07
Hungary	2010	46.89	28.84	23.98	31.34	22.91	1.96
Hungary	2012	43.92	28.21	12.82	29.53	31.10	3.43
Hungary	2014	50.17	25.26	26.79	31.87	23.39	1.87
Hungary	2016	33.28	22.13	22.91	23.04	10.37	1.45
Ireland	2002	23.32	16.91	8.12	18.81	15.20	2.87
Ireland	2004	18.57	18.17	12.68	18.30	5.89	1.46
Ireland	2006	24.33	19.71	17.45	21.59	6.88	1.39
Ireland	2008	26.37	19.84	17.61	21.40	8.77	1.50
Ireland	2010	22.31	12.09	15.09	17.51	7.22	1.48
Ireland	2012	23.88	19.92	9.66	19.51	14.22	2.47
Ireland	2014	24.68	12.33	10.36	17.85	14.32	2.38
Ireland	2016	24.97	18.18	8.71	17.79	16.26	2.87
Italy	2002	13.52	14.23	8.67	14.29	4.85	1.56
Italy	2012	19.28	16.06	15.45	17.57	3.84	1.25

Country	Year	Low	Medium	High	Total	Prevalence Difference	Prevalence Ratio
Italy	2016	15.64	10.12	8.01	13.04	7.63	1.95
Latvia	2008	53.03	37.36	32.61	39.74	20.41	1.63
Lithuania	2010	58.45	45.77	31.24	44.11	27.21	1.87
Lithuania	2012	29.67	29.75	23.79	29.46	5.88	1.25
Lithuania	2014	53.17	36.65	23.94	36.81	29.24	2.22
Lithuania	2016	47.24	35.00	24.92	34.77	22.32	1.90
Netherlands	2002	32.58	21.74	15.91	23.46	16.67	2.05
Netherlands	2004	31.30	24.44	21.10	24.71	10.20	1.48
Netherlands	2006	29.33	19.92	16.48	22.09	12.85	1.78
Netherlands	2008	23.62	23.98	18.24	22.13	5.38	1.30
Netherlands	2010	31.32	25.68	12.48	23.53	18.84	2.51
Netherlands	2012	29.74	21.65	17.09	22.40	12.65	1.74
Netherlands	2014	36.89	30.22	20.38	29.72	16.50	1.81
Netherlands	2016	40.05	28.40	17.20	27.58	22.85	2.33
Norway	2002	32.50	26.58	15.17	23.79	17.32	2.14
Norway	2004	36.22	25.48	23.34	27.34	12.88	1.55
Norway	2006	26.97	27.32	18.74	24.70	8.23	1.44
Norway	2008	37.12	27.67	16.19	26.59	20.93	2.29
Norway	2010	27.76	31.59	14.49	26.34	13.26	1.92
Norway	2012	29.77	27.12	17.02	24.45	12.75	1.75
Norway	2014	32.90	24.44	21.15	24.81	11.75	1.56
Norway	2016	36.88	28.66	17.66	26.44	19.22	2.09
Poland	2002	43.22	31.92	15.52	33.61	27.70	2.78
Poland	2004	46.68	33.87	21.65	36.68	25.03	2.16
Poland	2006	42.15	33.25	20.43	35.38	21.71	2.06
Poland	2008	35.93	32.51	27.44	31.65	8.49	1.31
Poland	2010	34.69	30.91	24.39	31.62	10.30	1.42
Poland	2012	35.47	30.83	15.95	30.32	19.52	2.22
Poland	2014	33.79	28.69	12.39	30.49	21.41	2.73
Poland	2016	30.24	26.85	18.15	26.85	12.09	1.67
Portugal	2002	16.84	0.00	0.00	14.99	16.84	
Portugal	2004	14.55	7.78	12.50	14.83	2.04	1.16
Portugal	2006	18.63	15.93	7.84	17.44	10.79	2.38
Portugal	2008	19.64	9.23	12.21	18.36	7.43	1.61
Portugal	2010	12.98	7.55	8.05	12.73	4.93	1.61
Portugal	2012	15.24	4.76	14.34	14.57	0.90	1.06
Portugal	2014	24.05	5.69	0.00	21.68	24.05	
Portugal	2016	18.68	10.76	6.45	17.23	12.23	2.90
Romania	2008	18.93	17.44	14.60	18.19	4.33	1.30
Slovakia	2004	41.90	27.06	26.26	31.00	15.64	1.60
Slovakia	2006	34.16	29.03	28.03	28.67	6.13	1.22
Slovakia	2008	28.31	20.93	18.35	21.59	9.96	1.54
Slovakia	2010	38.79	31.76	24.48	31.21	14.31	1.58
Slovakia	2012	29.04	25.62	24.04	26.72	5.01	1.21
Slovenia	2002	44.83	39.56	30.70	39.70	14.12	1.46
Slovenia	2004	45.33	39.49	27.57	40.42	17.76	1.64
Slovenia	2006	48.31	36.08	22.06	38.14	26.25	2.19
Slovenia	2008	38.20	36.92	22.44	35.92	15.76	1.70
Slovenia	2010	38.83	34.49	18.92	33.46	19.91	2.05
Slovenia	2012	30.57	31.00	21.52	29.49	9.05	1.42

Slovenia	2014	41.68	32.10	34.50	34.45	7.18	1.21
Slovenia	2016	47.87	30.32	22.62	31.03	25.25	2.12
Spain	2002	21.05	11.68	4.81	17.80	16.24	4.38
Spain	2004	16.87	9.43	8.21	14.40	8.65	2.05
Spain	2006	17.94	4.91	8.49	14.36	9.45	2.11
Spain	2008	13.98	12.87	12.93	13.02	1.05	1.08
Spain	2010	18.90	13.77	7.57	15.14	11.33	2.50
Spain	2012	15.38	13.68	6.95	13.06	8.43	2.21
Spain	2014	16.85	6.85	9.13	15.21	7.72	1.85
Spain	2016	17.77	12.37	8.15	13.08	9.62	2.18
Sweden	2002	28.04	30.28	21.76	27.18	6.28	1.29
Sweden	2004	28.43	24.64	19.64	26.26	8.79	1.45
Sweden	2006	37.28	29.05	21.27	27.97	16.00	1.75
Sweden	2008	21.81	29.94	24.10	26.11	-2.29	0.90
Sweden	2010	28.50	31.43	16.17	26.79	12.33	1.76
Sweden	2012	44.82	26.17	18.14	26.48	26.68	2.47
Sweden	2014	26.32	29.25	16.66	27.07	9.66	1.58
Sweden	2016	31.18	30.69	13.07	28.79	18.11	2.39
UK	2002	27.75	30.98	22.44	25.05	5.32	1.24
UK	2004	33.93	14.48	26.07	30.10	7.86	1.30
UK	2006	33.74	23.54	16.78	26.29	16.95	2.01
UK	2008	33.91	16.78	15.94	23.69	17.97	2.13
UK	2010	29.94	20.70	24.23	26.48	5.71	1.24
UK	2012	27.58	28.50	19.60	27.46	7.98	1.41
UK	2014	32.26	30.47	13.11	26.23	19.15	2.46
UK	2016	32.64	25.24	20.32	26.93	12.32	1.61

European Social Survey (ESS)

Females

Country	Year	Low	Medium	High	Total	Prevalence Difference	Prevalence Ratio
All countries	2002	29.04	27.67	21.40	27.28	7.64	1.36
All countries	2004	31.65	28.77	20.47	29.09	11.18	1.55
All countries	2006	32.58	28.77	21.98	29.00	10.60	1.48
All countries	2008	30.07	27.81	22.92	27.42	7.15	1.31
All countries	2010	32.24	27.85	22.74	28.67	9.50	1.42
All countries	2012	30.94	25.52	22.49	27.27	8.45	1.38
All countries	2014	33.20	30.01	23.80	30.55	9.39	1.39
All countries	2016	28.86	27.90	19.84	26.62	9.03	1.46
Austria	2002	28.73	26.00	20.74	25.97	7.98	1.38
Austria	2004	38.16	26.59	26.77	27.99	11.39	1.43
Austria	2006	38.93	23.55	11.42	29.82	27.52	3.41
Austria	2014	23.02	21.79	16.86	22.76	6.16	1.37
Austria	2016	34.56	19.16	9.80	21.71	24.76	3.53
Belgium	2002	32.07	20.40	16.63	25.62	15.44	1.93
Belgium	2004	27.88	21.83	13.88	24.01	14.01	2.01
Belgium	2006	35.36	23.03	16.12	24.50	19.23	2.19
Belgium	2008	33.33	23.26	21.76	26.34	11.57	1.53
Belgium	2010	40.02	22.83	18.94	26.02	21.08	2.11
Belgium	2012	41.25	26.26	24.85	29.32	16.40	1.66

Country	Year	Low	Medium	High	Total	Prevalence Difference	Prevalence Ratio
Belgium	2014	40.57	28.48	29.40	30.83	11.17	1.38
Belgium	2016	38.55	34.82	27.16	32.45	11.39	1.42
Bulgaria	2006	37.08	21.96	17.06	26.74	20.02	2.17
Bulgaria	2008	28.03	18.16	13.92	20.98	14.11	2.01
Bulgaria	2010	26.52	12.85	12.19	17.69	14.32	2.17
Bulgaria	2012	31.45	14.34	8.80	18.11	22.65	3.57
Cyprus	2006	13.03	8.46	7.46	13.25	5.57	1.75
Cyprus	2008	26.95	21.92	14.78	25.14	12.17	1.82
Cyprus	2010	36.45	21.20	10.26	26.55	26.20	3.55
Cyprus	2012	32.74	14.83	20.38	25.60	12.36	1.61
Czech R.	2002	49.02	35.03	31.49	39.64	17.53	1.56
Czech R.	2004	56.76	33.46	18.15	37.48	38.61	3.13
Czech R.	2008	60.83	38.16	24.73	40.65	36.10	2.46
Czech R.	2010	39.18	33.12	28.19	35.31	11.00	1.39
Czech R.	2012	48.25	27.36	25.40	29.96	22.84	1.90
Czech R.	2014	46.11	31.44	49.26	36.13	-3.15	0.94
Czech R.	2016	42.43	35.48	27.62	36.87	14.81	1.54
Denmark	2002	39.08	29.51	13.74	28.38	25.34	2.84
Denmark	2004	39.92	26.46	15.22	26.05	24.70	2.62
Denmark	2006	29.09	31.95	22.61	30.02	6.48	1.29
Denmark	2008	39.07	33.02	25.03	33.43	14.04	1.56
Denmark	2010	33.96	28.67	27.97	28.60	6.00	1.21
Denmark	2012	41.04	26.31	20.41	28.85	20.64	2.01
Denmark	2014	44.91	34.38	14.94	32.01	29.97	3.01
Estonia	2004	51.69	26.80	21.69	28.89	30.00	2.38
Estonia	2006	39.73	27.78	19.60	28.54	20.13	2.03
Estonia	2008	35.60	25.42	20.32	24.99	15.28	1.75
Estonia	2010	43.25	28.95	18.19	26.53	25.06	2.38
Estonia	2012	41.75	28.49	23.08	27.45	18.66	1.81
Estonia	2014	49.37	28.04	16.28	27.65	33.09	3.03
Estonia	2016	50.37	37.82	21.49	32.29	28.88	2.34
Finland	2002	38.52	33.43	22.53	32.56	16.00	1.71
Finland	2004	37.68	36.21	24.36	34.34	13.32	1.55
Finland	2006	40.44	37.43	24.31	33.62	16.13	1.66
Finland	2008	40.32	35.88	27.79	34.13	12.53	1.45
Finland	2010	48.22	33.56	26.89	33.63	21.33	1.79
Finland	2012	54.80	30.72	27.88	32.40	26.92	1.97
Finland	2014	52.01	36.15	32.62	36.55	19.39	1.59
Finland	2016	41.98	35.21	27.83	33.88	14.15	1.51
France	2002	33.43	27.49	19.27	28.82	14.16	1.74
France	2004	26.60	17.60	20.92	24.53	5.68	1.27
France	2006	29.17	21.25	26.55	25.11	2.62	1.10
France	2008	28.35	23.70	13.57	24.48	14.78	2.09
France	2010	29.84	27.10	13.60	26.98	16.24	2.19
France	2012	33.51	20.45	23.84	25.56	9.66	1.41
France	2014	34.82	27.00	8.78	29.42	26.04	3.97
France	2016	31.71	27.14	21.89	26.04	9.83	1.45
Germany	2002	44.23	28.98	18.73	31.67	25.50	2.36
Germany	2004	41.01	29.67	22.90	32.71	18.11	1.79
Germany	2006	34.03	29.96	24.92	30.54	9.11	1.37

Country	Year	Low	Medium	High	Total	Prevalence Difference	Prevalence Ratio
Germany	2008	36.97	28.31	27.04	29.05	9.93	1.37
Germany	2010	37.74	30.28	27.16	31.43	10.58	1.39
Germany	2012	40.20	31.02	29.01	32.97	11.18	1.39
Germany	2014	39.36	34.63	26.35	36.20	13.02	1.49
Germany	2016	46.81	37.54	27.95	38.72	18.87	1.68
Greece	2002	29.82	15.61	10.46	26.52	19.35	2.85
Greece	2004	23.47	14.61	8.78	21.35	14.69	2.67
Greece	2008	24.53	22.42	11.03	21.58	13.50	2.22
Greece	2010	16.17	14.62	21.85	16.84	-5.68	0.74
Hungary	2002	48.42	29.57	19.19	36.57	29.23	2.52
Hungary	2004	54.09	29.42	17.70	34.94	36.39	3.06
Hungary	2006	43.17	32.67	12.79	33.42	30.37	3.37
Hungary	2008	48.31	32.70	22.81	34.20	25.50	2.12
Hungary	2010	48.82	34.22	24.71	37.06	24.11	1.98
Hungary	2012	43.41	22.74	28.69	28.60	14.73	1.51
Hungary	2014	42.64	28.03	22.55	34.60	20.09	1.89
Hungary	2016	38.00	20.69	14.88	23.30	23.12	2.55
Ireland	2002	16.88	12.74	13.45	16.32	3.43	1.25
Ireland	2004	23.13	11.27	17.28	18.15	5.85	1.34
Ireland	2006	17.27	20.82	14.20	16.16	3.07	1.22
Ireland	2008	22.34	14.99	12.69	15.89	9.66	1.76
Ireland	2010	19.63	10.22	9.83	15.26	9.79	2.00
Ireland	2012	24.52	11.24	12.86	17.24	11.66	1.91
Ireland	2014	21.47	15.21	16.54	17.80	4.93	1.30
Ireland	2016	34.72	19.06	12.77	21.63	21.95	2.72
Italy	2002	13.44	3.58	2.64	12.21	10.80	5.09
Italy	2012	20.64	16.12	21.50	20.30	-0.86	0.96
Italy	2016	15.91	5.89	8.09	13.26	7.82	1.97
Latvia	2008	58.12	45.04	37.62	45.16	20.50	1.54
Lithuania	2010	65.80	52.00	45.25	50.89	20.55	1.45
Lithuania	2012	39.43	39.48	30.94	37.19	8.49	1.27
Lithuania	2014	59.79	40.63	24.14	39.25	35.65	2.48
Lithuania	2016	51.60	39.83	27.86	37.24	23.74	1.85
Netherlands	2002	31.45	34.04	26.05	30.77	5.41	1.21
Netherlands	2004	33.80	35.56	26.19	31.19	7.60	1.29
Netherlands	2006	35.67	34.35	25.06	32.57	10.61	1.42
Netherlands	2008	35.30	30.23	25.36	31.43	9.93	1.39
Netherlands	2010	41.72	31.35	21.39	32.28	20.33	1.95
Netherlands	2012	40.90	28.88	24.70	30.73	16.21	1.66
Netherlands	2014	40.53	31.05	25.62	34.02	14.91	1.58
Netherlands	2016	39.46	26.97	23.35	29.89	16.11	1.69
Norway	2002	37.35	29.82	21.15	29.72	16.20	1.77
Norway	2004	36.28	36.95	24.15	33.39	12.13	1.50
Norway	2006	34.41	33.34	18.21	29.37	16.20	1.89
Norway	2008	45.09	35.96	18.26	32.27	26.83	2.47
Norway	2010	41.80	31.50	26.04	30.83	15.76	1.61
Norway	2012	37.80	32.66	23.79	28.76	14.01	1.59
Norway	2014	42.08	36.93	22.32	35.73	19.76	1.89
Norway	2016	49.57	28.94	26.73	31.56	22.84	1.85
Poland	2002	46.52	38.39	23.96	37.18	22.56	1.94

Rubio Valverde JR, *et al. J Epidemiol Community Health* 2021; 75:712–720. doi: 10.1136/jech-2020-216141

Country	Year	Low	Medium	High	Total	Prevalence Difference	Prevalence Ratio
Poland	2004	41.09	37.05	23.79	38.13	17.30	1.73
Poland	2006	52.93	34.69	12.14	38.06	40.79	4.36
Poland	2008	56.78	32.34	33.63	35.73	23.15	1.69
Poland	2010	38.86	31.62	25.37	34.14	13.49	1.53
Poland	2012	38.62	27.68	29.63	32.97	8.99	1.30
Poland	2014	38.51	28.49	20.56	33.75	17.95	1.87
Poland	2016	37.67	26.57	21.16	30.39	16.52	1.78
Portugal	2002	18.72	3.52	0.00	17.62	18.72	
Portugal	2004	25.25	11.55	6.66	24.65	18.59	3.79
Portugal	2006	29.76	20.88	3.63	26.80	26.12	8.19
Portugal	2008	19.80	13.19	5.35	18.40	14.45	3.70
Portugal	2010	20.85	11.67	7.39	19.37	13.46	2.82
Portugal	2012	22.12	9.62	13.79	20.06	8.33	1.60
Portugal	2014	25.56	8.48	8.22	23.69	17.34	3.11
Portugal	2016	28.60	27.78	18.98	27.10	9.62	1.51
Romania	2008	26.06	19.87	6.00	22.81	20.07	4.35
Slovakia	2004	46.67	24.52	14.32	31.80	32.35	3.26
Slovakia	2006	43.29	28.29	14.06	31.06	29.23	3.08
Slovakia	2008	36.18	27.02	17.67	28.46	18.51	2.05
Slovakia	2010	44.54	28.11	21.85	31.57	22.69	2.04
Slovakia	2012	40.42	29.76	13.73	30.71	26.70	2.94
Slovenia	2002	42.71	39.72	26.40	40.18	16.32	1.62
Slovenia	2004	50.17	36.64	35.64	41.12	14.53	1.41
Slovenia	2006	52.45	32.36	21.36	38.64	31.09	2.46
Slovenia	2008	45.53	37.86	22.32	35.14	23.21	2.04
Slovenia	2010	44.73	30.38	16.68	34.10	28.05	2.68
Slovenia	2012	33.19	29.04	17.48	28.03	15.71	1.90
Slovenia	2014	48.03	38.70	17.46	41.97	30.57	2.75
Slovenia	2016	50.12	30.01	21.51	31.24	28.60	2.33
Spain	2002	22.04	4.89	3.88	20.48	18.16	5.67
Spain	2004	20.69	29.15	4.19	20.30	16.50	4.93
Spain	2006	25.41	19.42	4.34	22.51	21.06	5.85
Spain	2008	22.65	11.30	13.40	19.35	9.26	1.69
Spain	2010	22.72	17.27	14.38	21.83	8.34	1.58
Spain	2012	23.66	13.70	15.05	21.16	8.61	1.57
Spain	2014	20.51	13.22	3.10	18.94	17.42	6.63
Spain	2016	19.20	17.21	12.27	15.23	6.93	1.57
Sweden	2002	36.04	31.40	29.01	32.61	7.02	1.24
Sweden	2004	33.58	34.76	25.06	32.14	8.53	1.34
Sweden	2006	37.80	31.29	26.71	32.82	11.09	1.42
Sweden	2008	25.73	36.08	26.61	30.41	-0.89	0.97
Sweden	2010	28.26	31.50	23.52	28.22	4.74	1.20
Sweden	2012	34.07	32.33	24.48	29.99	9.58	1.39
Sweden	2014	56.29	35.10	28.78	36.66	27.51	1.96
Sweden	2016	25.73	33.83	28.75	31.94	-3.02	0.89
UK	2002	29.58	24.87	25.11	27.89	4.47	1.18
UK	2004	30.21	26.83	19.40	27.06	10.81	1.56

Rubio Valverde JR, *et al. J Epidemiol Community Health* 2021; 75:712–720. doi: 10.1136/jech-2020-216141

Online Resource 7

Table S7.1 Change in Global Activity Limitation (GALI) prevalence for each level of the phrasing correction for the pooled sample of 26 European Countries, (European Union Statistics on Income and Living Conditions 2005-2017)

	Sex	
**GALI Comparability**	**Males (n= 1,885,712)**	**Females (n= 2,192,091)**
Comparable [95% CI]	**ref**	**ref**
Partly Comparable [95% CI]	**-2.65** [-3.31 to 2.16]	**-1.87** [-2.32 to -1.40]
Not Comparable [95% CI]	**-1,15** [-1.82 to -0.05]	**-1.26** [-1.90 to -0.06]

Estimates are obtained from sex stratified logistic models using microdata as dependent variable a dichotomous GALI indicator:
logit(GALI) = b0 + b1(age) + b2(age)(age) + b3(education) +b4(year) + b5(year)*education + b6(country) + b7(GALI Comparability)

The inclusion of the GALI comparability variable in EU-SILC decreases the rate of change over 1 year of both prevalence and absolute educational inequalities. The average effect of the inclusion of the comparability variable in GALI prevalence is statistically significant reduction of 2.65 %-points for males and of 1.87%-points for females for partly comparable relative to the standard phrasing of the question. For not comparable questions, there is a statistically significant decrease of 1.15% in prevalence for males and of 1.26% for females .

Online Resource 8

Figure S8.1 Global Activity Limitation Indicator (GALI) disability prevalence change over 1 year (ages 30-79), for 26 countries and pooled sample, sex and survey (European Union Statistics on Income and Living Conditions 2005-2017; European Social Survey 2002-2016)

Males **– EU-SILC (Low Educated)**

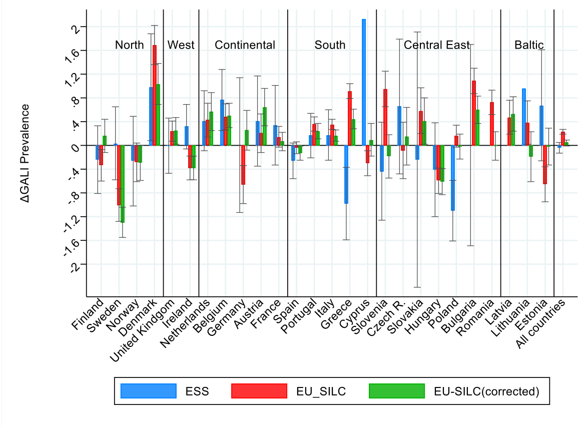

Males **– ESS(High Educated)**

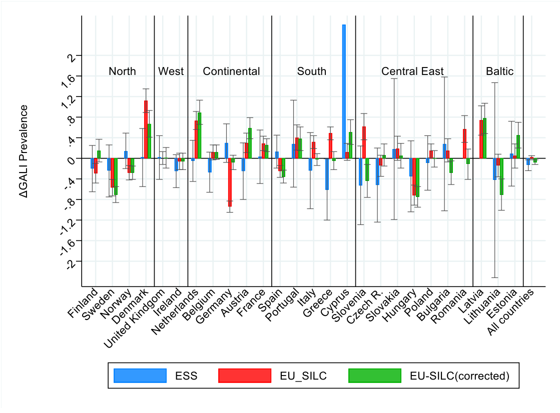

Females **– EU-SILC (High Educated)**

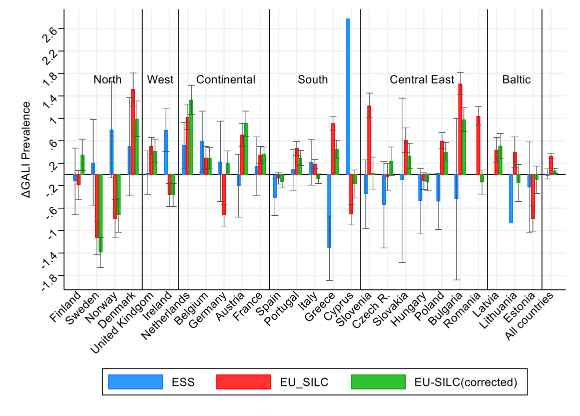

Females **– ESS(High Educated)**

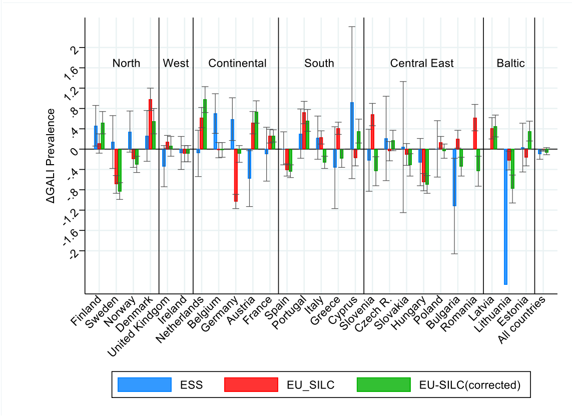

Estimates are obtained from country, sex stratified logistic models using EU-SILC and ESS microdata as dependent variable a dichotomous GALI indicator, stratified by sex, survey and country:

ESS and EU_SILC country stratified models
logit(GALI) = b0 + b1(age)*(age) + b2(education) +b3(year) + b4(year)*education

EU-SILC Corrected
logit(GALI)= b0 + b1(age)*(age) + b2(education) +b3(year) + b4(year)*education + b7(GALI Comparability)

We compute the average marginal effects for an increase of 1 year for each education level using the STATA command *margins,dydx.* These correspond to the annual change in prevalence presented. The annual average change in prevalence difference is estimated by subtracting the average marginal effects of the low and the high educated

For low educated men, countries where both surveys show a statistically significant increase in disability prevalence include the Netherlands, Belgium and Germany, while Spain and Hungary show a statistically significant decrease in prevalence. Countries where the trend for low educated men in both surveys is not statistically different from zero include Finland, the UK, France, Italy and Slovakia. For many countries, EU-SILC detects a significant trend for this group, while the ESS estimates are often not statistically significant. Women also show substantial heterogeneity between surveys and countries.

Online Resource 9

Table S9.1 Global Activity Limitation Indicator (GALI) disability prevalence change over 1 year, prevalence difference (low-high) and t-test results for comparison between surveys (ages 30-79) for 26 countries, by sex and survey

**Males**	**Low Educated**			**High Educated**			**Prevalence Difference**		
Country	*EU-SILC*	*ESS*	*Difference*	*EU-SILC*	*ESS*	*Difference*	*EU-SILC*	*ESS*	*Difference*
Finland	0.16	-0.24	0.40	0.15	-0.20	0.36	0.01	-0.04	0.04
	(0.26)	(0.41)	(0.38)	(0.17)	(0.38)	(0.32)	(0.97)	(0.92)	(0.93)
Sweden	**-1.30**	0.03	**-1.33**	**-0.71**	-0.24	-0.47	**-0.59**	0.28	**-0.86**
	(0.00)	(0.91)	(0.00)	(0.00)	(0.35)	(0.04)	(0.00)	(0.49)	(0.04)
Norway	**-0.29**	-0.26	-0.03	**-0.28**	0.14	**-0.42**	-0.01	-0.41	0.39
	(0.05)	(0.50)	(0.94)	(0.00)	(0.42)	(0.02)	(0.94)	(0.34)	(0.38)
Denmark	**1.03**	**0.98**	0.06	**0.67**	0.02	0.65	**0.36**	0.96	-0.60
	(0.00)	(0.03)	(0.92)	(0.00)	(0.96)	(0.09)	(0.04)	(0.08)	(0.27)
UK	**0.25**	-0.01	0.25	0.01	0.02	-0.01	**0.24**	-0.02	0.26
	(0.03)	(0.98)	(0.55)	(0.92)	(0.94)	(0.98)	(0.03)	(0.94)	(0.52)
Ireland	**-0.38**	0.32	**-0.70**	-0.06	-0.25	0.19	**-0.32**	**0.57**	**-0.88**
	(0.00)	(0.10)	(0.02)	(0.46)	(0.12)	(0.42)	(0.01)	(0.02)	(0.02)
Netherlands	**0.57**	0.41	0.16	**0.89**	-0.05	**0.95**	-0.32	0.47	-0.79
	(0.00)	(0.11)	(0.76)	(0.00)	(0.80)	(0.01)	(0.06)	(0.16)	(0.14)
Belgium	**0.50**	**0.77**	-0.26	0.12	-0.27	0.39	**0.38**	**1.04**	-0.66
	(0.00)	(0.00)	(0.47)	(0.08)	(0.18)	(0.11)	(0.00)	(0.00)	(0.12)
Germany	0.26	0.00	0.25	-0.08	0.30	-0.37	0.33	-0.29	0.63
	(0.13)	(1.00)	(0.69)	(0.29)	(0.12)	(0.17)	(0.06)	(0.63)	(0.34)
Austria	**0.64**	0.41	0.24	**0.59**	-0.25	**0.84**	0.06	0.66	-0.60
	(0.00)	(0.29)	(0.72)	(0.00)	(0.38)	(0.05)	(0.74)	(0.17)	(0.38)
France	0.07	0.34	-0.27	**0.26**	0.03	0.23	-0.19	0.31	-0.50
	(0.38)	(0.32)	(0.45)	(0.00)	(0.92)	(0.43)	(0.06)	(0.47)	(0.28)
Spain	-0.13	-0.26	0.13	**-0.36**	0.13	-0.49	**0.23**	-0.39	0.62
	(0.04)	(0.09)	(0.67)	(0.00)	(0.42)	(0.15)	(0.00)	(0.08)	(0.09)
Portugal	**0.24**	0.17	0.07	**0.38**	0.28	0.10	-0.15	-0.12	-0.03
	(0.00)	(0.39)	(0.79)	(0.00)	(0.51)	(0.83)	(0.24)	(0.81)	(0.95)
Italy	**0.16**	0.17	-0.01	-0.02	-0.24	0.22	**0.18**	0.42	-0.23
	(0.00)	(0.42)	(0.99)	(0.72)	(0.52)	(0.75)	(0.00)	(0.34)	(0.76)
Greece	**0.44**	**-0.98**	**1.42**	-0.05	**-0.61**	0.56	**0.49**	-0.37	0.86
	(0.00)	(0.00)	(0.00)	(0.61)	(0.05)	(0.28)	(0.00)	(0.39)	(0.07)
Cyprus	0.09	**2.13**	**-2.04**	**0.51**	**2.60**	**-2.09**	**-0.42**	-0.47	0.05
	(0.51)	(0.01)	(0.01)	(0.00)	(0.01)	(0.00)	(0.00)	(0.72)	(0.95)
Slovenia	-0.18	-0.44	0.26	**-0.44**	-0.53	0.08	0.26	0.09	0.17
	(0.32)	(0.30)	(0.70)	(0.01)	(0.18)	(0.88)	(0.20)	(0.88)	(0.81)
Czech R.	0.15	0.66	-0.50	0.06	-0.52	0.58	0.09	1.18	-1.09
	(0.54)	(0.26)	(0.56)	(0.57)	(0.15)	(0.13)	(0.73)	(0.09)	(0.23)
Slovakia	**0.41**	-0.24	0.66	0.05	0.18	-0.13	0.36	-0.42	0.78
	(0.04)	(0.82)	(0.49)	(0.67)	(0.80)	(0.82)	(0.11)	(0.74)	(0.47)
Hungary	**-0.61**	-0.41	-0.20	**-0.75**	-0.35	-0.40	0.14	-0.06	0.20
	(0.00)	(0.31)	(0.69)	(0.00)	(0.32)	(0.37)	(0.34)	(0.91)	(0.76)
Poland	-0.02	**-1.10**	**1.08**	-0.01	-0.09	0.08	-0.01	**-1.01**	1.00
	(0.87)	(0.00)	(0.07)	(0.91)	(0.74)	(0.85)	(0.94)	(0.01)	(0.12)
Bulgaria	**0.60**	0.00	0.60	**-0.28**	0.28	-0.56	**0.88**	-0.28	1.16

	(0.00)	(1.00)	(0.27)	(0.02)	(0.67)	(0.29)	(0.00)	(0.80)	(0.11)
Romania	-0.01			-0.11			0.10		
	(0.93)			(0.45)			(0.54)		
Latvia	**0.53**			**0.78**			-0.25		
	(0.00)			(0.00)			(0.22)		
Lithuania	-0.19	0.96	-1.15	**-0.71**	-0.42	-0.28	**0.52**	1.38	-0.87
	(0.38)	(0.49)	(0.25)	(0.00)	(0.66)	(0.70)	(0.02)	(0.41)	(0.40)
Estonia	-0.02	0.67	-0.69	**0.45**	0.09	0.36	**-0.47**	0.58	-1.05
	(0.90)	(0.16)	(0.24)	(0.00)	(0.78)	(0.44)	(0.01)	(0.31)	(0.11)

Females

	Low Educated			***High Educated***			***Prevalence Difference***		
Country	*EU-SILC*	*ESS*	*Difference*	*EU-SILC*	*ESS*	*Difference*	*EU-SILC*	*ESS*	*Difference*
Finland	**0.35**	-0.12	0.47	**0.52**	**0.46**	0.06	-0.16	-0.58	0.41
	(0.01)	(0.70)	(0.30)	(0.00)	(0.03)	(0.87)	(0.26)	(0.11)	(0.38)
Sweden	**-1.39**	0.21	**-1.60**	**-0.83**	0.14	**-0.97**	**-0.56**	0.07	-0.63
	(0.00)	(0.59)	(0.00)	(0.00)	(0.59)	(0.00)	(0.00)	(0.88)	(0.17)
Norway	**-0.72**	**0.80**	**-1.52**	**-0.30**	0.34	**-0.64**	**-0.42**	0.46	-0.88
	(0.00)	(0.07)	(0.00)	(0.00)	(0.09)	(0.01)	(0.02)	(0.35)	(0.08)
Denmark	**0.99**	0.50	0.49	**0.55**	0.26	0.29	**0.44**	0.24	0.20
	(0.00)	(0.26)	(0.32)	(0.00)	(0.30)	(0.45)	(0.01)	(0.65)	(0.69)
UK	**0.42**	0.03	0.39	0.06	-0.34	0.39	**0.37**	0.37	0.00
	(0.00)	(0.87)	(0.30)	(0.57)	(0.10)	(0.26)	(0.00)	(0.20)	(1.00)
Ireland	**-0.37**	**0.79**	**-1.16**	-0.09	-0.08	-0.02	**-0.28**	**0.87**	**-1.14**
	(0.00)	(0.00)	(0.00)	(0.25)	(0.65)	(0.94)	(0.04)	(0.00)	(0.00)
Netherlands	**1.33**	**0.52**	**0.81**	**0.98**	-0.08	**1.06**	**0.35**	0.60	-0.25
	(0.00)	(0.01)	(0.05)	(0.00)	(0.72)	(0.01)	(0.02)	(0.06)	(0.61)
Belgium	**0.29**	**0.59**	-0.29	-0.02	**0.70**	**-0.72**	**0.31**	-0.12	0.42
	(0.00)	(0.03)	(0.40)	(0.83)	(0.00)	(0.01)	(0.01)	(0.73)	(0.31)
Germany	**0.21**	0.23	-0.02	-0.09	**0.59**	**-0.68**	**0.30**	-0.36	0.66
	(0.05)	(0.52)	(0.96)	(0.28)	(0.01)	(0.04)	(0.01)	(0.39)	(0.18)
Austria	**0.91**	-0.20	**1.11**	**0.73**	**-0.58**	**1.31**	0.17	0.38	-0.20
	(0.00)	(0.48)	(0.01)	(0.00)	(0.04)	(0.00)	(0.20)	(0.34)	(0.71)
France	**0.37**	0.15	0.21	**0.26**	-0.10	0.36	0.11	0.25	-0.14
	(0.00)	(0.57)	(0.47)	(0.00)	(0.72)	(0.20)	(0.23)	(0.51)	(0.73)
Spain	**-0.13**	**-0.41**	0.28	**-0.44**	0.01	-0.45	**0.31**	-0.42	**0.73**
	(0.04)	(0.01)	(0.38)	(0.00)	(0.94)	(0.19)	(0.00)	(0.07)	(0.05)
Portugal	**0.30**	0.09	0.21	**0.56**	0.30	0.26	**-0.27**	-0.22	-0.05
	(0.00)	(0.64)	(0.38)	(0.00)	(0.22)	(0.47)	(0.02)	(0.49)	(0.89)
Italy	-0.08	0.22	-0.30	**-0.26**	0.22	-0.48	**0.18**	-0.01	0.19
	(0.08)	(0.29)	(0.57)	(0.00)	(0.30)	(0.50)	(0.01)	(0.99)	(0.81)
Greece	**0.45**	**-1.31**	**1.76**	**-0.18**	-0.36	0.18	**0.63**	-0.95	**1.58**
	(0.00)	(0.00)	(0.00)	(0.04)	(0.38)	(0.70)	(0.00)	(0.06)	(0.00)
Cyprus	-0.17	**2.78**	**-2.95**	**0.35**	0.92	-0.56	**-0.53**	1.86	**-2.38**
	(0.17)	(0.00)	(0.00)	(0.00)	(0.23)	(0.39)	(0.00)	(0.10)	(0.00)
Slovenia	0.02	-0.35	0.37	**-0.43**	-0.22	-0.21	**0.46**	-0.12	0.58
	(0.86)	(0.27)	(0.46)	(0.00)	(0.47)	(0.67)	(0.00)	(0.78)	(0.29)
Czech R.	0.24	-0.54	0.78	0.17	0.21	-0.03	0.06	-0.75	0.81
	(0.07)	(0.17)	(0.12)	(0.09)	(0.62)	(0.93)	(0.69)	(0.19)	(0.18)

	Low Educated			***High Educated***			***Prevalence Difference***		
Country	*EU-SILC*	*ESS*	*Difference*	*EU-SILC*	*ESS*	*Difference*	*EU-SILC*	*ESS*	*Difference*
Slovakia	**0.33**	-0.10	0.43	**-0.31**	0.04	-0.35	**0.64**	-0.14	0.78
	(0.00)	(0.89)	(0.40)	(0.01)	(0.95)	(0.49)	(0.00)	(0.89)	(0.27)
Hungary	-0.14	-0.47	0.33	**-0.70**	-0.26	-0.44	**0.56**	-0.22	0.77
	(0.06)	(0.11)	(0.34)	(0.00)	(0.28)	(0.27)	(0.00)	(0.57)	(0.14)
Poland	**0.40**	-0.48	0.88	-0.04	0.01	-0.05	**0.44**	-0.48	0.93
	(0.00)	(0.06)	(0.07)	(0.58)	(0.97)	(0.91)	(0.00)	(0.20)	(0.08)
Bulgaria	**0.98**	-0.44	**1.42**	**-0.34**	**-1.12**	**0.78**	**1.33**	0.68	0.65
	(0.00)	(0.55)	(0.00)	(0.00)	(0.02)	(0.05)	(0.00)	(0.44)	(0.26)
Romania	-0.14			-**0.43**			0.30		
	(0.21)			(0.00)			(0.06)		
Latvia	**0.51**			**0.45**			0.05		
	(0.00)			(0.00)			(0.73)		
Lithuania	-0.15	-0.87	0.72	**-0.78**	**-2.67**	**1.88**	**0.63**	1.79	-1.16
	(0.37)	(0.39)	(0.32)	(0.00)	(0.00)	(0.00)	(0.00)	(0.16)	(0.12)
Estonia	-0.10	-0.23	0.13	**0.35**	**0.03**	0.32	**-0.45**	-0.26	-0.19
	(0.45)	(0.58)	(0.76)	(0.00)	(0.01)	(0.35)	(0.00)	(0.59)	(0.60)

Estimates are obtained from country, sex stratified logistic models using EU-SILC and ESS microdata as dependent variable a dichotomous GALI indicator, stratified by sex, survey and country:

ESS and EU_SILC country stratified models
Logit(GALI) = b0 + b1(age)*(age) + b2(education) +b3(year) + b4(year)*education

EU-SILC Corrected
Logit(GALI)= b0 + b1(age)*(age) + b2(education) +b3(year) + b4(year)*education + b7(GALI Comparability)

We compute the average marginal effects for an increase of 1 year for each education level using the STATA command *margins,dydx.* These correspond to the annual change in prevalence presented. The annual average change in prevalence difference is estimated by subtracting the average marginal effects of the low and the high educated

Rubio Valverde JR, *et al. J Epidemiol Community Health* 2021; 75:712–720. doi: 10.1136/jech-2020-216141

Online Resource 10

Figure S10.1 Slope index of inequality and Relative Index of inequality of the Global Activity Limitation Indicator (GALI) for 26 countries and pooled sample, sex and survey (European Union Statistics on Income and Living Conditions 2005-2017; European Social Survey 2002-2016)

EU-SILC – Males (SII)

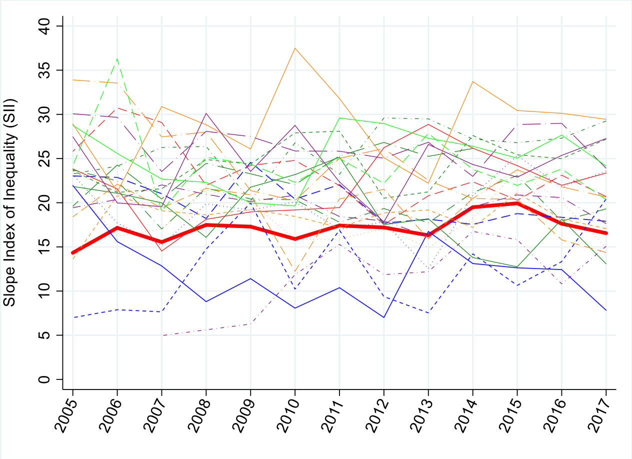

ESS- Males (SII)

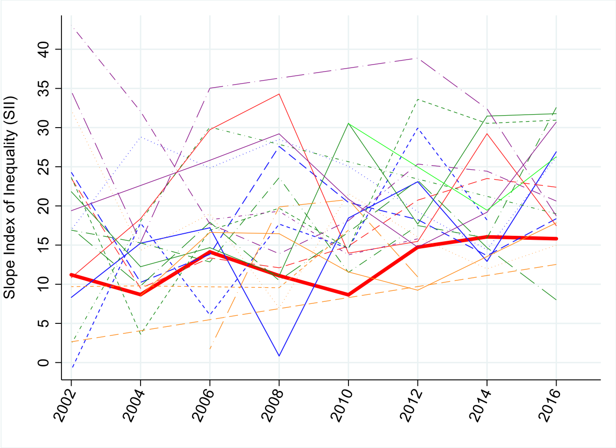

EU-SILC – Males (SII)

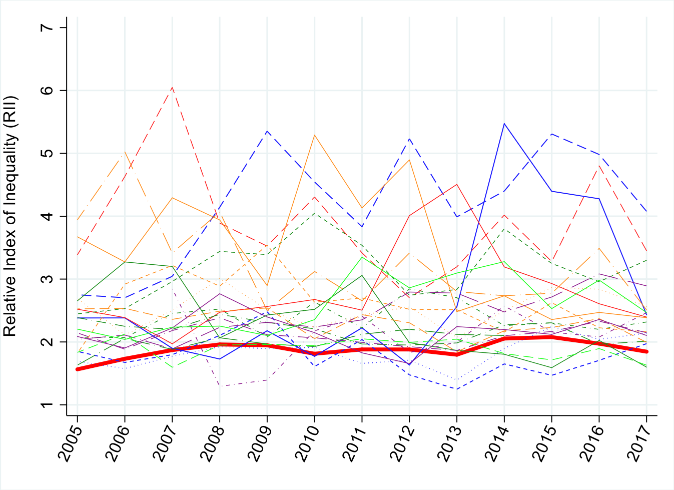

ESS- Males (SII)

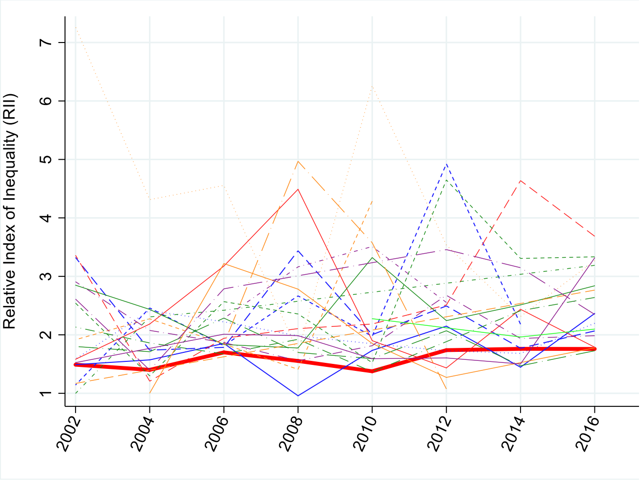

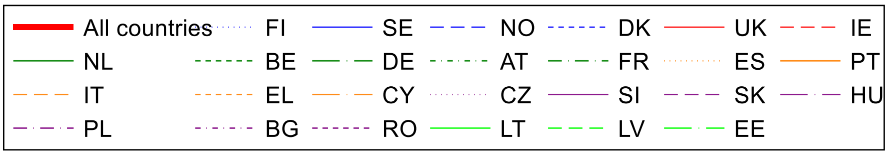

Females- EU SILC (SII)

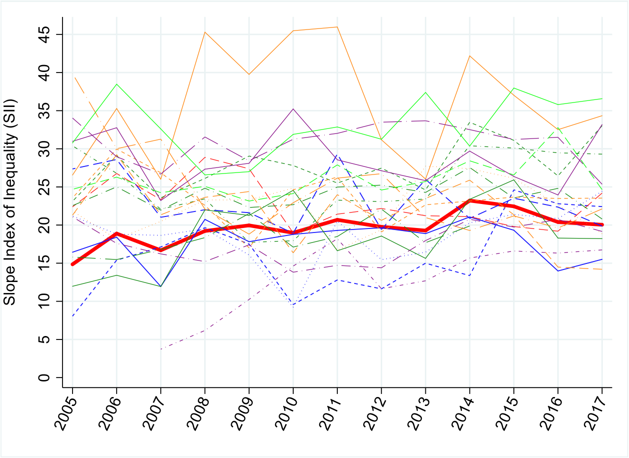

Females – ESS (SII)

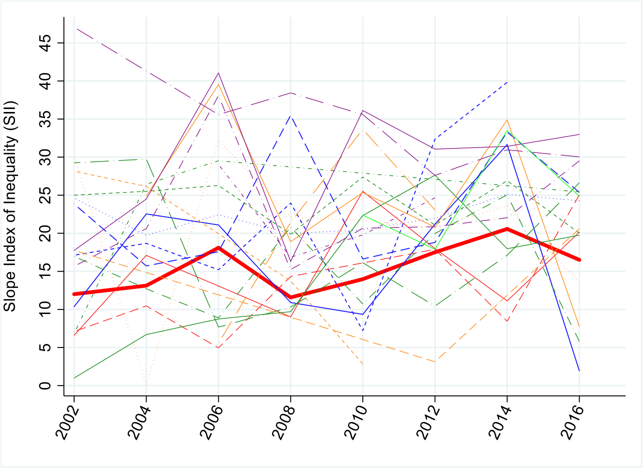

Females- EU SILC (RII)

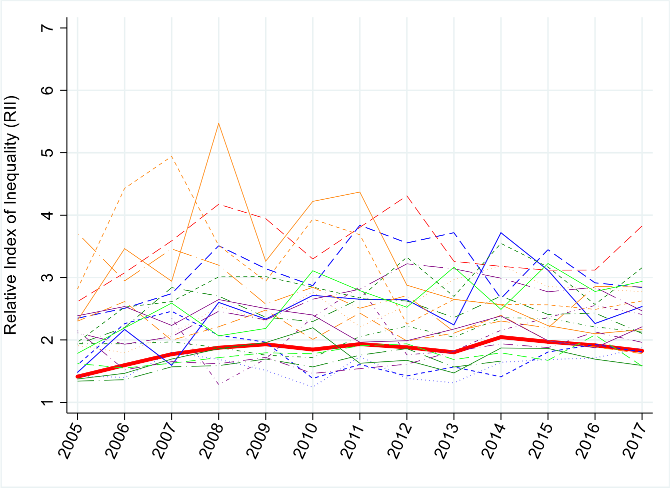

Females – ESS (RII)

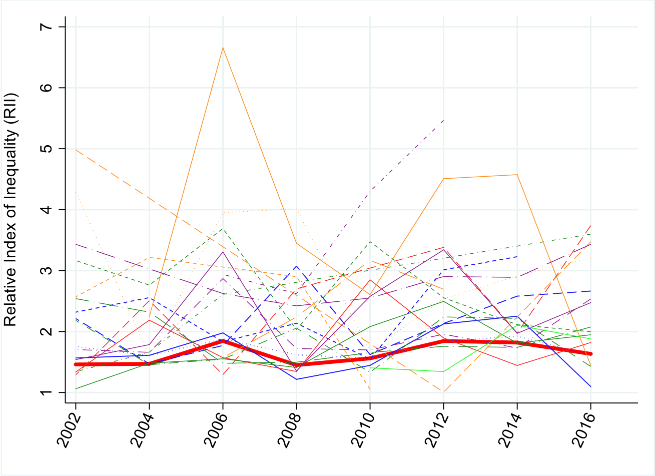

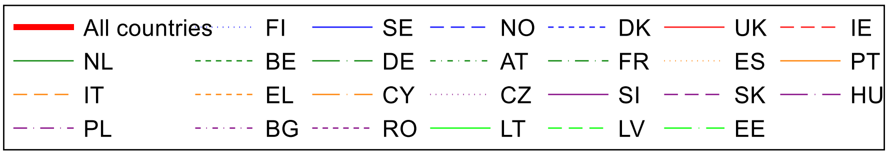

Table S10.2 Global Activity Limitation Indicator (GALI) educational inequalities (prevalence difference, ratio, slope index of inequality and relative index of inequality) change in 1 year (ages 30-79) for the pooled sample of 26 European Countries from , by education, sex and survey (European Union Statistics on Income and Living Conditions 2005-2017; European Social Survey 2002-2016) and T-tests between survey estimates

**Males**	**EU- SILC (2005-2017)** [a] **n= 1,885,712**			**ESS (2002-2016)** [b] **n=110,603**		*Difference between surveys (2)-(1b)* [e]
**Δ in Inequality in 1 year**	*(1)*	*(2)* [c]	*Average Inequality* [d]	*(1b)*	*Average Inequality* [d]	
*Prev. difference** [95% CI]*	**0.28** [0.21 to 0.34]	**0.11** [0.03 to 0.17]	12.05 [11.26 to 12.84]	-0,03 [-0,27 to 0,21]	9.92 [9.25 to 11.95]	0.14 [-0.14 to 0.41]
*Prev. ratio** [95% CI]*	**0.026** [0.017 to 0.028]	**0.010** [0.004 to 0.015]	1.650 [1.591 to 1.710]	-0,003 [-0,015 to 0,004]	1.532 [1.421 to 1.701]	-0.013 [-0.002 to 0.004]
*SII *** [95% CI]*	**0.21** [0.01 to 0.41]		17.09 [16.13 to 18.01]	0.39 [-0.03 to 0.82]	12.47 [9.13 to 15.80]	-0.18 [-0.56 to 0.2026]
*RII *** [95% CI]*	**0.020** [0.002 to 0.039]		1.876 [1.806 to 1.949]	0.021 [-0.002 to 0.045]	1.587 [1.390 to 1.812]	-0.001 [-0.028 to 0.055]

**Females**	**EU- SILC (2005-2017)** [a] **n= 2,192,091**			**ESS (2002-2016)** [b] **n=128,401**		*Difference between surveys (2)-(1b)* [e]
**Δ Inequality in 1 year**	*(1)*	*(2)* [c]	*Average Inequality* [d]	*(1b)*	*Average Inequality* [d]	
*Prev. difference** [95% CI]*	**0.26** [0.19 to 0.32]	**0.12** [0.04 to 0.18]	12.53 [11.76 to 13.36]	0.02 [-0,21 to 0,23]	9.19 [7.13 to 11.05]	0.10 [-0.19 to 0.38]
*Prev. ratio ** [95% CI]*	**0,016** [0,011 to 0,019]	**0,009** [0,004 to 0,012]	1.563 [1.522 to 1.612]	0,001 [-0,013 to 0,015]	1.421 [1.30 to 1.54]	0.008 [-0.010 to 0.025]
*SII *** [95% CI]*	**0.40** [0.15 to 0.65]		19.57 [18.57 to 20.56]	0.34 [-0.17 to 0.86]	15.53 [12.03 to 19.03]	0.06 [-0.40 to 0.527]
*RII *** [95% CI]*	**0.028** [0.007 to 0.049]		1.831 [1.767 to 1.897]	0.014 [-0.016 to 0.044]	1.645 [1.454 to 1.861]	0.014 [-0.018 to 0.465]

*Estimates are obtained from sex stratified linear probability models using microdata as dependent variable a dichotomous GALI indicator:

(1) and (1b) logit(GALI) = b0 + b1(age) + b2(age)(age) + b3(education) +b4(year) + b5(year)*education + b6(country)

(2) logit(GALI) = b0 + b1(age) + b2(age)(age) + b3(education) +b4(year) + b5(year)*education + b6(country) + b7(GALI Comparability)

**Slope Index of Inequality (SII) and Relative Index of Inequality (RII) are obtained through generalized linear modals that include education (low, medium, high) and age in 5 year age categories(30-34; 35-39;…) as covariates and are stratified by year. Trends are obtained through linear regression of RII/SII as independent variable and year as independent variable using robust standard error.

[a] European Union Statistics on Income and Living Conditions (EU-SILC) annual microdata between 2005 and 2017. Member countries in EU-SILC use variants of the GALI question over time.

[b] European Social Survey (ESS) biannual microdata between 2002 and 2016.ESS uses the same version of the GALI question for all countries and years. This version omits the 6 month time frame of the standard GALI question.

[c] GALI comparability estimates include baseline model plus a 3 level categorical variable relative to standard question phrasing (comparable, partially comparable, not comparable)

GALI question change estimates include baseline model and a binary variable that takes the value of 1 if question changed prior to last year. Both GALI variables were obtained for 2005-2012 from the EUROSTAT document "Overview of the implementation of the GALI question in EU-SILC", and can be obtained from:
https://circabc.europa.eu/webdav/CircaBC/ESTAT/health/Library/working_group_2012/documents/Item%209.1%20HLY%20annex%202%20-%20overview%20tables%20and%20notes.pdf

[d] Average age-standardized GALI prevalence over the corresponding period for each survey using the EU 2013 standard population for all countries included in the sample. [e] Two sample t-test between the EU-SILC and ESS coefficients **. Significant at the 95% level in bold**

BMJ Publishing Group Limited (BMJ) disclaims all liability and responsibility arising from any reliance placed on this supplemental material which has been supplied by the author(s)

Online Resource 11

Table S11.1- Robustness analyses - Global Activity Limitation Indicator (GALI) prevalence change (%) in 1 year (ages 30-79) for the pooled sample of 26 European Countries from , by education and survey (European Union Statistics on Income and Living Conditions 2005-2017; European Social Survey 2002-2016) -Males

Males **EU-SILC (2005-2017)**

**Education level**	***Main***		*With % education[*]*		*Post 2008[**]*		*Probability weight only[***]*	
	(1)	*(2)[c]*	*(1)*	*(2)[c]*	*(1)*	*(2)[c]*	*(1)*	*(2)[c]*
Low	**0.13**	0.04	**0.13**	0.05	**0.12**	0.05	**0.22**	**0.05**
[95% CI]	[0.08 to 0.17]	[-0.01 to 0.10]	[0.08 to 0.18]	[-0.00 to 0.10]	[0.06 to 0.18]	[0.00 to 0.09]	[0.18 to 0.26]	[0.01 to 0.08]
Medium	**-0.06**	-0.02	**-0.06**	-0.00	0.01	**-0.05**	**0.05**	**-0.08**
[95% CI]	[-0.09 to -0.02]	[-0.06 to 0.02]	[-0.09 to -0.02]	[-0.06 to 0.02]	[-0.03 to 0.05]	[-0.10 to -0.02]	[0.02 to 0.08]	[-0.10 to -0.06]
High	**-0.15**	**-0.07**	**-0.15**	**-0.07**	**0.05**	-0.03	**0.05**	**-0.06**
[95% CI]	[-0.19 to -0.11]	[-0.12 to -0.02]	[-0.19 to -0.11]	[-0.12 to -0.02]	[0.01 to 0.10]	[-0.01 to -0.02]	[0.01 to 0.09]	[-0.09. -0.03]
n	1,809,402		1,809,402		1,413,759		1,809,402	

Males **ESS (2002-2016)** **EU-SILC (2005-2017)**

**Education level**	***Main***		*With % education[*]*		*Probability weight only[***]*		*Excl. Romania and Latvia[****]*	
		(1b)		*(1b)*		*(1b)*	*(1)*	*(2)[c]*
Low		-0.02		-0.01		0.05	**0.12**	0.06
[95% CI]		[-0.19 to 0.16]		[-0.20 to 0.17]		[-0.07 to 0.18]	[0.07 to 0.17]	[-0.01 to 0.11]
Medium		0.11		0.11		**-0.09**	-0.07	**-0.02**
[95% CI]		[-0.01 to 0.23]		[-0.11 to 0.24]		[-0.17 to 0.01]	[-0.10 to -0.03]	[-0.06 to 0.03]
High		0.01		0.01		-0.10	-**0.15**	**-0.07**
[95% CI]		[-0.14 to 0.15]		[-0.15 to 0.18]		[-0.21 to 0.01]	[-0.19 to -0.11]	[-0.12 to -0.02]
n	110,603		110,603		110,603		1,698,405	

Estimates are obtained from sex stratified logistic models using microdata as dependent variable a dichotomous GALI indicator:
(1) and (1b) logit(GALI) = b0 + b1(age) + b2(age)(age) + b3(education) +b4(year) + b5(year)*education + b6(country)
(2) logit(GALI) = b0 + b1(age) + b2(age)(age) + b3(education) +b4(year) + b5(year)*education + b6(country) + b7(GALI Comparability)
a European Union Statistics on Income and Living Conditions (EU-SILC) annual microdata between 2005 and 2017. Member countries in EU-SILC use variants of the GALI question over time.
b European Social Survey (ESS) biannual microdata between 2002 and 2016.ESS uses the same version of the GALI question for all countries and years. This version omits the 6 month time frame of the standard GALI question.
c GALI comparability estimates include baseline model plus a 3 level categorical variable relative to standard question phrasing (comparable, partially comparable, not comparable) for EU-SILC only.
* Models include the percentage of education measured for each country, survey, sex and year as part of the regressions.
**Models exclude information prior to 2008
***Models use the normalized probability weights provided by EU-SILC and ESS in the regression analyses. Main estimates use product of population and probability weighs in analyses.

****Models exclude Romania and Latvia

Table S11.2- Robustness analyses - Global Activity Limitation Indicator (GALI) prevalence change (%) in 1 year (ages 30-79) for the pooled sample of 26 European Countries from , by education and survey (European Union Statistics on Income and Living Conditions 2005-2017; European Social Survey 2002-2016) - Females

Females **EU-SILC (2005-2017)**

**Education level**	***Main***		*With % education*[*]		*Post 2008*[**]		*Probability weight only*[***]	
	(1)	*(2)*[c]	*(1)*	*(2)*[c]	*(1)*	*(2)*[c]	*(1)*	*(2)*[c]
Low	**0.10**	0.00	**0.12**	0.02	**0.17**	0.06	**0.33**	**0.10**
[95% CI]	[0.06 to 0.14]	[-0.05 to 0.05]	[0.08 to 0.16]	[-0.03 to 0.07]	[0.12 to 0.23]	[-0.01 to 0.13]	[0.30 to 0.37]	[0.06 to 0.14]
Medium	**-0.13**	**-0.09**	**-0.13**	**-0.09**	0.02	**-0.04**	**0.10**	**-0.07**
[95% CI]	[-0.17 to -0.09]	[-0.14 to -0.05]	[0.17 to -0.09]	[-0.13 to -0.04]	[-0.01 to 0.05]	[-0.01 to -0.07]	[0.07 to 0.13]	[-0.10 to 0.04]
High	**-0.16**	**-0.13**	**-0.18**	**-0.14**	0.03	-0.01	0.02	**-0.13**
[95% CI]	[-0.21 to -0.10]	[-0.18 to -0.07]	[-0.23 to 0.16]	[-0.20 to -0.08]	[-0.01 to 0.08]	[-0.06 to 0.03]	[-0.03 to 0.06]	[-0.17 to -0.08]
n	2,192,091		2,192,091		1,611,428		2,192,091	

Females **ESS (2002-2016)**

**Education level**	***Main***		*With % education*[*]		*Probability weight only*[***]		*Excl. Romania and Latvia*[****]	
		(1b)		*(1b)*		*(1b)*		
Low		0.02		0.03		0,06	**0.10**	**0.03**
[95% CI]		[-0.13 to 0,.7]		[-0.13 to 0.19]		[-0,06 to 0,18]	[0.06 to 0.014]	[-0.03 to 0.07]
Medium		**0.17**		**0.17**		0,03	**-0.14**	**-0.10**
[95% CI]		[0.05 to 0.29]		[0.04 to 0.29]		[-0,06 to -0,11]	[-0.17 to -0.11]	[-0.15 to -0.06]
High		-0.00		-0.00		-0.03	**-0.14**	**-0.11**
[95% CI]		[-0.18 to 0.18]		[-0.17 to 0.17]		[-0.14 to 0,09]	[-0.18 to -0.10]	[-0.16 to -0.06]
n	128,401		128,401		83,464		1,927,708	

Estimates are obtained from sex stratified logistic models using microdata as dependent variable a dichotomous GALI indicator:

(1) and (1b) logit(GALI) = b0 + b1(age) + b2(age)(age) + b3(education) +b4(year) + b5(year)*education + b6(country)

(2) logit(GALI) = b0 + b1(age) + b2(age)(age) + b3(education) +b4(year) + b5(year)*education + b6(country) + b7(GALI Comparability)

a European Union Statistics on Income and Living Conditions (EU-SILC) annual microdata between 2005 and 2017. Member countries in EU-SILC use variants of the GALI question over time.

b European Social Survey (ESS) biannual microdata between 2002 and 2016.ESS uses the same version of the GALI question for all countries and years. This version omits the 6 month time frame of the standard GALI question.

c GALI comparability estimates include baseline model plus a 3 level categorical variable relative to standard question phrasing (comparable, partially comparable, not comparable) for EU-SILC only.

* Models include the percentage of education measured for each country, survey, sex and year as part of the regressions.

**Models exclude information prior to 2008

***Models use the normalized probability weights provided by EU-SILC and ESS in the regression analyses. Main estimates use product of population and probability weighs in analyses.

****Models exclude Romania and Latvia

Table S11.3- Robustness analyses - Global Activity Limitation Indicator (GALI) educational inequalities (prevalence difference and ratio) change in 1 year (ages 30-79) for the pooled sample of 26 European Countries from , by education, sex and survey (European Union Statistics on Income and Living Conditions 2005-2017; European Social Survey 2002-2016) - Males

Males	EU-SILC (2005-2017)							
	Main		*With % education**		*Post 2008***		*Probability weight only****	
**Inequality**	*(1)*	*(2)c*	*(1)*	*(2)c*	*(1)*	*(2)c*	*(1)*	*(2)c*
Prev. Diff [95% CI]	**0.28** [0.21 to 0.34]	**0.11** [0.03 to 0.17]	**0.30** [0.22 to 0.36]	**0.16** [0.09 to 0.23]	0.07 [-0.01 to 0.14]	**0.08** [0.01 to 0.14]	**0.17** [0.12 to 0.27]	**0.11** [0.07 to 0.15]
Prev Ratio [95% CI]	**0.026** [0.017 to 0.028]	**0.010** [0.004 to 0.015]	**0.017** [0.003 to 0.011]	**0.011** [0.007 to 0.015]	0.001 [-0.004 to 0.006]	**0.005** [0.001 to 0.009]	**0.007** [0.003 to 0.011]	**0.008** [0.004 to 0.011]
n	1,885,712		1,885,712		1,413,759		1,809,402	

Males	ESS (2002-2016)						EU-SILC (2005-2017)	
**Education level**	***Main***		*With % education**		*Probability weight only****		*Excl. Romania and Latvia*****	
		(1b)		*(1b)*		*(1b)*	*(1)*	*(2)c*
Prev. Diff [95% CI]		-0.03 [-0.27 to 0.21]		-0.03 [-0.27 to 0.21]		0.16 [0.00 to 0.32]	**0.28** [0.21 to 0.35]	**0.13** [0.06 to 0.19]
Prev Ratio [95% CI]		-0.003 [-0.015 to 0.004]		-0.002 [-0.021 to 0.027]		0.014 [0.000 to 0.027]	**0.016** [0.003 to 0.011]	**0.009** [0.004 to 0.013]
n	110,603		110,603		110,603		1,698,405	

Estimates are obtained from sex stratified logistic models using microdata as dependent variable a dichotomous GALI indicator:
(1) and (1b) logit(GALI) = b0 + b1(age) + b2(age)(age) + b3(education) +b4(year) + b5(year)*education + b6(country)
(2) logit(GALI) = b0 + b1(age) + b2(age)(age) + b3(education) +b4(year) + b5(year)*education + b6(country) + b7(GALI Comparability)
a European Union Statistics on Income and Living Conditions (EU-SILC) annual microdata between 2005 and 2017. Member countries in EU-SILC use variants of the GALI question over time.
b European Social Survey (ESS) biannual microdata between 2002 and 2016.ESS uses the same version of the GALI question for all countries and years. This version omits the 6 month time frame of the standard GALI question.
c GALI comparability estimates include baseline model plus a 3 level categorical variable relative to standard question phrasing (comparable, partially comparable, not comparable) for EU-SILC only.
* Models include the percentage of education measured for each country, survey, sex and year as part of the regressions.
**Models exclude information prior to 2008
***Models use the normalized probability weights provided by EU-SILC and ESS in the regression analyses. Main estimates use product of population and probability weighs in analyses.

****Models exclude Romania and Latvia

BMJ Publishing Group Limited (BMJ) disclaims all liability and responsibility arising from any reliance placed on this supplemental material which has been supplied by the author(s)

Table S11.4- Robustness analyses - Global Activity Limitation Indicator (GALI) educational inequalities (prevalence difference and ratio) change in 1 year (ages 30-79) for the pooled sample of 26 European Countries from , by education, sex and survey (European Union Statistics on Income and Living Conditions 2005-2017; European Social Survey 2002-2016) - Females

Females	**EU-SILC (2005-2017)[a]**							
	Main		*With % education*[*]		*Post 2008*[**]		*Probability weight only*[***]	
**Inequality**	*(1)*	*(2)[c]*	*(1)*	*(2)[c]*	*(1)*	*(2)[c]*	*(1)*	*(2)[c]*
Prev. Diff [95% CI]	**0.26** [0.19 to 0.32]	**0.12** [0.04 to 0.18]	**0.31** [0.26 to 0.36]	**0.23** [0.17 to 0.28]	**0.13** [0.07 to 0.19]	**0.08** [0.02 to 0.13]	**0.31** [0.26 to 0.36]	**0.23** [0.17 to 0.28]
Prev Ratio [95% CI]	**0.016** [0.011 to 0.019]	**0.009** [0.004 to 0.012]	**0.013** [0.009 to 0.015]	**0.012** [0.009 to 0.015]	**0.004** [0.001 to 0.007]	**0.003** [0.001 to 0.006]	**0.013** [0.009 to 0.015]	**0.012** [0.009 to 0.015]
n	2,192,091		2,192,091		1,611,428		2,192,091	

Females	**ESS (2002-2016)[b]**						**EU-SILC (2005-2017)[c]**	
	Main		*With % education*[*]		*Probability weight only*[***]		*Excl. Romania and Latvia*[****]	
**Education level**		*(1)*		*(1)*		*(1)*		
Prev. Diff [95% CI]		0.02 [-0.21 to 0.23]		0.03 [-0.20 to 0.27]		0.08 [-0.08 to 0.24]	**0.24** [0.18 to 0.30]	**0.13** [0.07 to 0.19]
Prev Ratio [95% CI]		0.001 [-0.013 to 0.015]		0.002 [-0.013 to 0.016]		0.005 [-0.006 to 0.015]	**0.012** [0.009 to 0.015]	**0.004** [0.001 to 0.007]
n	110,603		110,603		110,603		1,927,708	

Estimates are obtained from sex stratified logistic models using microdata as dependent variable a dichotomous GALI indicator:

(1) and (1b) logit(GALI) = b0 + b1(age) + b2(age)(age) + b3(education) +b4(year) + b5(year)*education + b6(country)

(2) logit(GALI) = b0 + b1(age) + b2(age)(age) + b3(education) +b4(year) + b5(year)*education + b6(country) + b7(GALI Comparability)

a European Union Statistics on Income and Living Conditions (EU-SILC) annual microdata between 2005 and 2017. Member countries in EU-SILC use variants of the GALI question over time.

b European Social Survey (ESS) biannual microdata between 2002 and 2016.ESS uses the same version of the GALI question for all countries and years. This version omits the 6 month time frame of the standard GALI question.

c GALI comparability estimates include baseline model plus a 3 level categorical variable relative to standard question phrasing (comparable, partially comparable, not comparable) for EU-SILC only.

* Models include the percentage of education measured for each country, survey, sex and year as part of the regressions.

**Models exclude information prior to 2008

***Models use the normalized probability weights provided by EU-SILC and ESS in the regression analyses. Main estimates use product of population and probability weighs in analyses.

****Models exclude Romania and Latvia

Rubio Valverde JR, *et al. J Epidemiol Community Health* 2021; 75:712–720. doi: 10.1136/jech-2020-216141
